# SPECTRA: A Conceptual Framework to Bridge Praxis and Remap Relational Violence in India Using a Complex Trauma Lens

**DOI:** 10.3390/bs16050814

**Published:** 2026-05-19

**Authors:** Maitrayee Sen, Snigdhaa Rajvanshi, Stuti Khandelwal, Simantini Ghosh

**Affiliations:** Department of Psychology, Ashoka University, Sonipat 131029, India

**Keywords:** domestic violence, intimate partner violence, complex trauma, continuum of violence, continuous trauma

## Abstract

Domestic Violence affects 1 in 3 women worldwide. Empirical evidence from India suggests that women and girls experience a continuum of violence and discrimination from prenatal stages till death in families that largely continue to operate within a dominantly patriarchal framework. However, the literature on domestic violence in India suffers from problems pertaining to reductive and episodic framing, focusing on short-term prevalence, and frames the impact on survivors largely in terms of clinical constructs such as anxiety, depression, and PTSD. This work argues for a broader, thematic framing of domestic and familial violence and contends that the psychological sequelae of this kind of chronic and systemic discrimination and violence cannot be captured using rigid clinical constructs that dominate psychological literature. We propose a conceptual framework, i.e., SPECTRA (Socially and Psychologically Embedded Continuous Trauma in Relational Architecture), which is partially aligned with the propositions of complex trauma. However, we also critique the origin of complex trauma within hegemonic psychiatry and highlight the need for creating a culturally adapted expansion—to shift the emphasis from an individually rooted, diagnostic framework to a culturally contextualized continuous trauma framework. We utilize seven illustrative case studies to define the tenets of the SPECTRA model.

## 1. Introduction

Violence against women (VAW) remains one of the most pervasive forms of gender-based inequality globally, with intimate partner violence (IPV) representing its most extensively studied manifestation. IPV is defined as physical, sexual, or psychological harm inflicted by a current or former intimate partner and has been associated with profound physical, psychological, and social consequences (Campbell, 2002; Bacchus et al., 2018; Dokkedahl et al., 2022; Meyer et al., 2023). Global estimates suggest that approximately 27–30% of women have experienced physical or sexual IPV in their lifetime, with prevalence rates in South Asia exceeding global averages (World Health Organization, 2021; Coll et al., 2020). In India, large-scale national surveys indicate that approximately one in three women experience spousal violence, with most perpetrators being known family members, including intimate partners and marital relatives (IIPS & ICF, 2021; UN Women, 2022), and independent research groups suggest incidence is higher (41%; Kalokhe et al., 2017).

However, the conceptualization of domestic violence (DV) in India extends beyond intimate partner violence alone. Women are embedded within extended kinship networks, mostly cohabiting with marital families, where violence may be perpetrated by not only spouses but also in-laws and other relatives (Visaria, 2008; Gangoli & Rew, 2011; Raghavan & Iyengar, 2020). Furthermore, gendered discrimination, witnessing violence on mothers and experiencing violence frequently precede marriage, occurring within natal families and shaping developmental trajectories long before intimate partnerships are formed. Cumulatively, these exposures form deeply embedded patriarchal relational practices in which violence is not episodic but continuous, evolving across the lifespan (Hossain & Heise, 2017). They also increasingly embed relational scripts built around norms built around obedience, subservice and self-sacrifice. These form attitudes and beliefs that lead women to undermine their own agency, safety and ability to seek any form of redress for relational violence in their future or offer them any mechanism of stopping the imposed guilt on them from damaging their confidence and self-esteem. Sociocultural attitudes, beliefs, and internalized norms that women owe their devotion, allegiance and obedience to their husbands and marital families (Pew Research Center, 2022) add to their chances of violent victimization. Despite this entrenched structural continuity, dominant frameworks in psychology and public health continue to conceptualize domestic violence primarily through episodic (e.g., 12-month prevalence), event-based (e.g., dowry violence, wife-beating), and clinically oriented models.

This limitation has profound implications for both measurement and psychological theorization. While feminist scholarship across many disciplines, sociology, and public health research have extensively documented the structural and socio-cultural dimensions of domestic violence in India, mainstream academic psychology has largely approached its impact through individualized clinical constructs such as depression, anxiety, and post-traumatic stress disorder (PTSD), without adequately theorizing the developmental and relational embedding of trauma (Vindhya, 2007; Sabri et al., 2022).

The result is a theoretical and conceptual vacuum in psychological frameworks capable of capturing the continuous, relational, and socially embedded nature of domestic violence in patriarchal contexts.

This article addresses a key limitation in existing trauma frameworks by examining how domestic violence is experienced as a continuous, relationally embedded process rather than as a series of discrete events. It develops **the SPECTRA model** (Socially and Psychologically Embedded Continuous Trauma in Relational Architectures) as a theory-building conceptual framework grounded in narrative analysis of survivors’ accounts in India. The study is qualitative in design and draws on a set of illustrative cases rather than representative sampling. These narratives are used as theoretically informative material to develop and refine the model by identifying recurring relational and psychological processes across participants’ experiences.

### 1.1. Literature Review

We begin with a formulation of a theoretical gap by a strategic review of extant literature on DV in India, describing its pitfalls and limitations (Section 1.2.1). We then highlight the lack of a suitable psychological framework despite considerable indirect evidence that psychological processes are quite important in how women experience and negotiate violence in intimate relationships and marriages, and its necessity (Section 1.2.2). We follow this up with a discussion on the origins of theorization on complex trauma and its congruence with sequelae of DV but also explain why the existing complex trauma framework cannot be used as is in DV research in India (Section 1.2.3). Finally, we launch our proposed framework SPECTRA (Section 1.3).

### 1.2. Theoretical and Conceptual Gaps

#### 1.2.1. Episodic Framing, Definitional and Measurement Pitfalls

One of the most significant limitations of domestic violence research lies in its episodic framing. Epidemiological studies and national surveys, including the National Family Health Survey (NFHS), predominantly measure violence within restricted temporal windows, such as past-year or past six-month prevalence (Kalokhe et al., 2017; Mittal et al., 2023). While these approaches provide important prevalence estimates, they fragment what is fundamentally a longitudinal and developmental phenomenon. Lifetime prevalence rates consistently exceed past-year estimates, underscoring the cumulative nature of violence exposure (Kalokhe et al., 2017).

**Figure 1:**
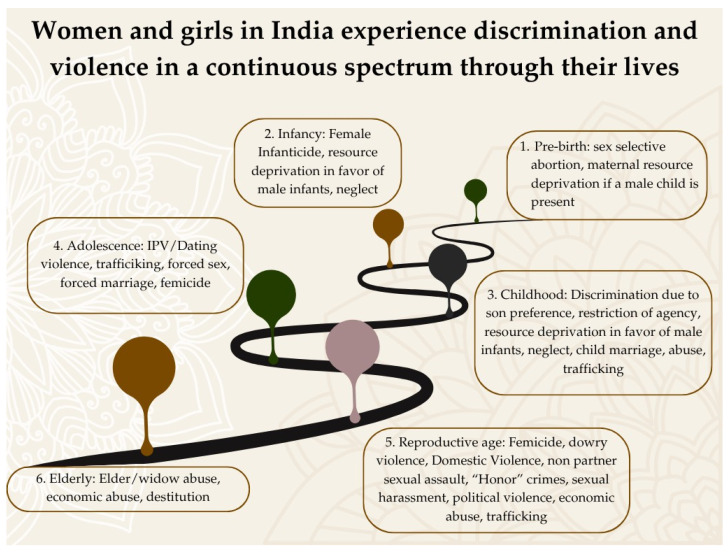
A Life-Course Continuum of Gendered Discrimination and Violence in India. This figure synthesizes insights from a theory-oriented review of existing literature to illustrate how gender-based discrimination and violence are distributed across the life course in the Indian context. Beginning in the pre-birth period and extending through infancy, childhood, adolescence, reproductive years, and older age, the figure highlights the cumulative and interconnected nature of gendered harms, including resource deprivation, restricted autonomy, and multiple forms of interpersonal and structural violence.

Such measurement frameworks obscure the developmental continuity through which violence is normalized and internalized. Gender socialization processes, including early exposure to gender hierarchy, restricted autonomy, and normalized coercion, shape expectations and relational dynamics long before formal partnerships emerge (Chattopadhyay, 2018; Kosakowska-Berezecka et al., 2022). These early experiences likely establish cognitive, emotional, and behavioral scaffolds underlying how women interpret, negotiate, and respond to violence later in life. Current frameworks for DV do not account for such developmental embedding, when it comes to violent victimization, later in life, nor can they accommodate the fact that relational violence affects Indian women across all ages that has been recognized in the literature pertaining to VAW (Heise, 2011) (Figure 1).

Rather than representing discrete or isolated events, the continuum emphasizes how early experiences of gendered inequality shape subsequent vulnerabilities, contributing to a patterned and ongoing trajectory of relational and structural constraint. This synthesis informed the conceptual framing of the study and the development of the SPECTRA model.

Furthermore, measurement tools frequently lack cultural contextualization and are constructed based on disparate definitions of DV that may not have much relevance in India (Kalokhe et al., 2015). Large scale surveys mostly rely on measuring the most easily accessible reported data on physical and sexual violence against women, whereas several other trajectories of abuse, based on hierarchies of power and control are left unaddressed. The data also suffers from chronic underreporting, i.e., less than 10% women in India formally report instances of DV and 70% do not disclose it at all, even to friends or family (NFHS-5, 2019–21). Many psychometric instruments commonly built on “Gold-standard” measures of IPV used in India are adapted from Western cultures without adequate modification, limiting their ability to capture culturally specific forms of coercion, including control over mobility, financial autonomy, reproductive decisions, and social participation (Kalokhe et al., 2016; Rathod et al., 2011). Methodological concerns, including variability in reporting based on interviewer characteristics, further undermine the reliability of large-scale prevalence estimates collected through the National Family and Health Surveys (NFHS-5; Singh et al., 2023). These limitations collectively contribute to an incomplete and fragmented understanding of domestic violence as a continuous relational phenomenon.

#### 1.2.2. The Psychological Vacuum in Theorization

Despite extensive documentation of domestic violence across disciplines, academic psychology in India has not developed a comprehensive theoretical framework to understand its psychological sequelae rooted in a feminist, relational epistemological stance (Vindhya, 2020). While sociological, anthropological, and feminist scholarship have examined domestic violence through structural, legal, and cultural lenses (Ahmed-Ghosh, 2004; Agnes & D’Mello, 2015), psychological research has largely remained confined to diagnostic frameworks that emphasize clinical pathology.

Most psychological studies examine domestic violence primarily as a risk factor for psychiatric disorders such as depression, anxiety, PTSD, and suicidality (Patel et al., 2021; Gilmoor et al., 2019). While these constructs are important, they represent only a narrow subset of psychological consequences. Diagnostic frameworks inherently prioritize symptom identification and clinical classification, often overlooking broader alterations in identity, relational functioning, and meaning-making that may not meet diagnostic thresholds, alongside how psychological trauma is inevitably linked to sociocultural factors (Davar, 2002; Burstow, 2003).

This clinical emphasis also reflects serious structural biases in knowledge production. Historically, psychiatric epidemiology in India underestimated psychological distress among women due to their underrepresentation in clinical samples, reinforcing erroneous assumptions about gender and mental health (Vindhya, 2007). Subsequent feminist analyses demonstrated that community samples revealed higher levels of psychological distress among women who had several social access barriers before psychological service could be available to them, highlighting the limitations of clinical sampling frameworks (Davar, 1999).

Importantly, diagnostic frameworks locate trauma primarily within individuals, obscuring the relational and structural conditions that produce psychological harm. This individualization of trauma risks reinforcing survivor responsibility for recovery while minimizing the role of patriarchal systems, economic dependence, and social control in sustaining violence (Sabri et al., 2022, 2024). Consequently, psychology lacks a conceptual framework capable of integrating developmental, relational, and socio-cultural dimensions of trauma within patriarchal family systems commonly encountered in India.

#### 1.2.3. Complex Trauma: Promise and Limits

Complex trauma theory, first articulated by Herman (1992a, 1992b), offers a critical departure from episodic models of trauma by conceptualizing trauma as chronic, relational, and inescapable. Unlike PTSD, which was originally developed to describe responses to discrete traumatic events, complex trauma emphasizes prolonged exposure to interpersonal violence within conditions of captivity and coercive control (Herman, 1992a, 1992b; Cloitre, 2020; van der Kolk, 2014).

Complex trauma theory identifies multiple domains of psychological alteration, including expanded and interacting signatures of both posttraumatic sequelae and depressive presentations leading to persistent dysphoria, somatization, alterations in consciousness, and problems related to affect regulation, self-perception, relational functioning, and systems of meaning (Herman, 1992a, 1992b; Luxenberg et al., 2001; van der Kolk et al., 2005). These domains reflect pervasive alterations in identity and relational experience rather than isolated symptom clusters. Importantly, these features closely align with the relational dynamics of domestic violence, where survivors remain embedded within coercive relational environments.

However, despite its conceptual strengths, complex trauma theory has also been incorporated into psychiatric diagnostic frameworks, including complex PTSD (cPTSD) within ICD-11 (Brewin et al., 2017). Critics have argued that diagnostic incorporation risks reproducing the same individualizing tendencies that obscure structural and relational causes of trauma (Burstow, 2003; Davar, 2002). By framing trauma primarily as individual pathology, diagnostic models may inadvertently depoliticize experiences rooted in systemic gender inequality.

Furthermore, complex trauma theory was largely developed within Western clinical contexts and, plausibly, cannot account for culturally specific relational and familial structures characteristic of collectivist societies such as India. In these contexts, trauma often unfolds within extended kinship networks, patriarchal authority structures, and intergenerational relational systems, necessitating culturally grounded conceptual expansion.

### 1.3. The SPECTRA Model

To address the conceptual limitations outlined above, we propose the **SPECTRA model (Socially and Psychologically Embedded Continuous Trauma in Relational Architectures)**. This framework understands domestic violence as a continuous and relationally embedded process. It does not locate trauma only in discrete events or individual psychopathology. Instead, it situates trauma within **relational architectures**. These are historically and culturally structured systems that organize power, obligation, and social regulation within intimate and family relationships. This life-course continuity (Figure 1) requires a framework that can account for how these relational structures produce and sustain trauma over time.

This framework is derived from seven illustrative cases and is intended as a theory-building model rather than a finalized or generalizable structure. The SPECTRA model organizes recurring patterns across survivors’ narratives into four interrelated domains. These domains are not stages. They are analytically distinct processes that operate together over time. As a set, they show how trauma becomes developmentally embedded, relationally sustained, psychologically internalized, and actively negotiated. Table 1 provides a structured overview of these domains. Supplementary Table S1 presents further analytic detail.

The first domain, developmental psychic embedding, refers to the early internalization of relational norms and asymmetries of power. These shape how individuals understand obligation, adjustment, and relational endurance. This domain places later experiences of violence within a longer developmental trajectory.

The second domain, relational captivity, captures conditions in which autonomy becomes constrained within ongoing relationships. The focus is not on isolated acts of violence. Instead, it is on sustained patterns of constraint. These patterns are organized within relational systems.

The third domain, psychological and somatic adaptations, describes how prolonged relational constraint becomes internalized. This occurs at the level of affect, identity, and embodied experience. Within this framework, these changes are understood as contextually shaped responses. They are not treated as discrete diagnostic symptoms.

The fourth domain, negotiation and adaptive repair, focuses on how individuals respond to and navigate these conditions. It includes efforts to recalibrate relationships and restore a sense of self. It also includes attempts to create liveable conditions within structural limits.

Taken together, these domains conceptualize domestic violence as a continuous relational trauma process. Developmental conditions, relational structures, internal adaptations, and negotiated responses remain interconnected. The SPECTRA model shifts the analytic focus away from episodic events. It instead emphasizes the processes through which trauma is produced, sustained, and navigated over time. For a detailed formulation of the model domain, see Supplementary File S8.

**Figure 2:**
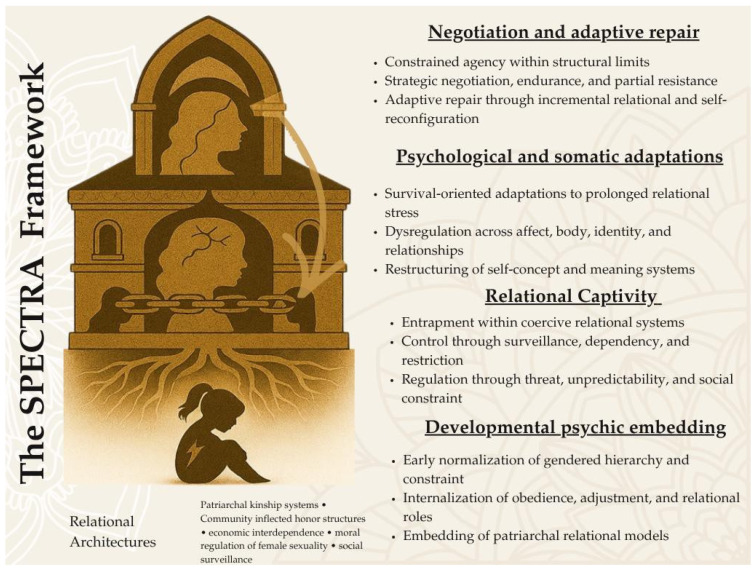
The SPECTRA (Socially and Psychologically Embedded Continuous Trauma in Relational Architecture) Framework: From Developmental Embedding to Adaptive Repair. The framework is grounded in narrative analysis of participants’ lived experiences and reflects an iterative analytic process linking inductive coding, thematic consolidation, and abductive theoretical engagement (see Tables S3A–D). Rather than representing discrete stages, the domains are interrelated and may overlap across the lifespan. The model emphasizes relational and socio-cultural structures as constitutive of trauma, moving beyond event-based and symptom-centered frameworks.

Figure 2 presents the SPECTRA framework as a process-oriented model of domestic violence, illustrating how women’s experiences unfold across four interrelated domains: developmental psychic embedding, relational captivity, psychological and somatic adaptations, and negotiation and adaptive repair. The model traces a developmental progression from early socialization within gendered relational hierarchies to entrapment within coercive relational systems, followed by adaptive changes across affective, somatic, relational, and meaning-making processes, and finally to forms of constrained agency expressed through negotiation and partial repair.

**Table 1:** The SPECTRA Framework: Domains, Core Processes, Mechanisms, Cultural Context, and Conceptualization. This table summarizes the SPECTRA framework as a process-oriented model of continuous, relational, and developmentally embedded trauma. It presents four interrelated domains—developmental psychic embedding, relational captivity, psychological and somatic adaptations, and negotiation and adaptive repair—along with their core psychological processes, underlying mechanisms, culturally specific contexts, and distinctions from clinical trauma frameworks. The table provides a conceptual synthesis derived from the analytic integration of narrative data and theoretical engagement. (See also Supplementary File S8, Table S1).

SPECTRA Domain	Core Psychological Process	Mechanisms of Operation	Cultural Specificities	Distinction from Clinical Trauma Frameworks
Developmental Psychic Embedding	Early embedding of gendered relational hierarchies through developmental socialization within family systems	Repeated exposure to gendered hierarchy and asymmetrical power; internalization of obedience, adjustment, and relational roles; early normalization of constraint through familial interactions and observation	Patrilineal kinship systems; son preference; moralization of female sacrifice; early restriction of autonomy and mobility; reinforcement of obedience and hierarchical gender roles	Trauma is developmentally embedded rather than triggered by discrete traumatic events
Relational Captivity	Entrapment within coercive, hierarchical, and unpredictable relational systems	Economic dependency and restricted mobility; social isolation and disconnection from natal support systems; surveillance and behavioral regulation within extended family structures; control over sexuality, reproduction, and financial decision-making; regulation through threat, unpredictability, and emotional manipulation	Joint-family systems; stigma around divorce; community and caste surveillance; marital expectations of endurance and silence	Captivity is sustained by relational systems rather than solely by individual perpetrators
Psychological and Somatic Adaptations	Survival-oriented adaptations to prolonged relational constraint affecting affect, body, identity, and meaning systems	Affect dysregulation and emotional instability; somatic expression of distress; dissociation and altered consciousness; internalized shame, guilt, and damaged self-concept; relational withdrawal and mistrust; restructuring of beliefs and meaning systems	Cultural silence around mental health; stigma surrounding emotional distress; somatic idioms of distress and normalization of endurance	Clinical models describe symptom clusters (e.g., PTSD, cPTSD, DESNOS); SPECTRA interprets these as adaptive responses to ongoing relational captivity
Negotiation and Adaptive Repair	Constrained agency expressed through strategic negotiation, endurance, and incremental reconstruction of self and relational roles	Strategic silence and compliance; negotiation within structural constraints; partial or staged exit (e.g., separation); boundary-setting and relational recalibration; rebuilding agency through work and social networks	Dependence on natal family support; culturally embedded coping strategies; negotiated autonomy within relational systems rather than exit	Emphasizes constrained agency and adaptive navigation rather than purely pathological or deficit-based responses

#### Distinction

SPECTRA is not proposed as a competing diagnosis but as a culturally grounded conceptual expansion for analyzing continuous relational trauma in patriarchal family systems. There are 3 key differences between the SPECTRA framework and clinical trauma constructs such as PTSD (DSM5, American Psychiatric Association, 2013), PTSD (ICD-11), C-PTSD (ICD11; Brewin et al., 2017; Cloitre, 2020), DESNOS (Luxenberg et al., 2001; van der Kolk et al., 2005) and DTD (Ford et al., 2013; van der Kolk et al., 2019). For a detailed comparison please see Supplementary File S8.

**Diagnostic vs. Conceptual**
  All other constructs are diagnostic or proto-diagnostic. SPECTRA is explicitly non-diagnostic.
**Event-Based vs. Continuous**
  DSM-5 and ICD-11 PTSD require event thresholds. SPECTRA conceptualizes normalized, developmentally layered trauma.
**Intrapsychic vs. Structural**
  Existing constructs locate pathology primarily within the individual. SPECTRA locates trauma within relational architectures and structural inequality.

## 2. Materials and Methods

### 2.1. Study Design and Rationale

This study employed a qualitative, theory-building design to address conceptual limitations in the psychological understanding of domestic violence (DV) in India. Specifically, the study aimed to develop a culturally grounded conceptual framework that captures DV as a continuous, relational, and developmental process, rather than as discrete or event-based occurrences.

The study combined two complementary components:

(a) *A structured, theory-oriented review* of existing literature (see Supplementary Methods);
(b) *In-depth narrative interviews* analyzed using a multi-level narrative analytic framework.

The literature review was conducted to identify gaps in existing psychological and socio-cultural models of DV, particularly their emphasis on episodic, diagnostic, or decontextualized accounts. The narrative interviews provided empirical material to examine how experiences of violence, constraint, and adaptation unfold across time, relationships, and socio-cultural contexts.

The analytic approach was iterative and abductive. Initial coding and theme development were grounded in participants’ narratives, followed by engagement with theoretical frameworks, particularly from complex trauma literature, as sensitizing constructs to refine interpretation. The resulting SPECTRA framework emerged through this interaction between empirical material and theoretical engagement.

This study is based on a small set of illustrative cases and is intended as a theory-building contribution rather than a representative or generalizable account.

### 2.2. Participants and Sampling

Seven urban, educated Indian women (aged 28–60 years) participated in the study. Participants represented diverse relational contexts, including arranged and love marriages, separation, divorce, and non-marital intimate relationships. Five participants were based in Kolkata, one in Haryana, and one in Hyderabad.

Participants were recruited through a combination of convenience and snowball sampling using social media outreach and personal networks. Inclusion criteria required participants to have experienced at least one significant intimate relationship (defined as a relationship of at least six months’ duration or involving cohabitation). Experience of violence was not required for inclusion. This allowed the study to capture a broader range of relational experiences, including forms of constraint, control, and abuse, that may not be explicitly recognized as violence. All participants provided informed consent prior to participation. Ethical approval was obtained from the Institutional Review Board at Ashoka University.

### 2.3. Data Collection

Data were collected through in-depth online interviews using a narrative-oriented, semi-structured interview guide. The design prioritized eliciting extended, temporally organized accounts rather than brief, categorical responses.

Interviews explored participants’ experiences across multiple domains, including:

- Natal family environments
- Intimate and marital relationships
- Work–life negotiations and gendered labor
- Psychological and emotional experiences across the lifespan
- Processes of meaning-making and interpretation

Open-ended prompts were combined with flexible probing to examine developmental trajectories, relational dynamics, and socio-cultural contexts. Some probes were informed by complex trauma literature but were used as sensitizing tools rather than diagnostic categories.

Interviews were audio-recorded with consent. Participants were informed of confidentiality, their right to withdraw, and the voluntary nature of participation. Pseudonyms were used and identifying details were removed. Interviewers were trained in qualitative interviewing, trauma sensitivity, and psychological first aid. Participants were debriefed following the interview and provided with referral resources where needed.

Reflexive field notes and analytic memos were maintained during and after interviews to document contextual observations and emerging analytic insights.

### 2.4. Analytic Framework: Narrative Analysis

We employed a multi-level narrative analytic approach (Bamberg, 2021), which treats narratives as meaning-making practices through which individuals construct identity, negotiate relational roles, and situate themselves within broader cultural contexts.

Analysis proceeded across three interconnected levels:

- Thematic level: identification of recurring experiential patterns, developmental trajectories, and continuity across life stages;
- Positioning level: examination of how participants positioned themselves and others, including constructions of agency, responsibility, and self-worth;
- Socio-cultural level: analysis of broader cultural discourses (e.g., adjustment, duty, respectability) as structuring forces shaping relational dynamics.

This framework enabled the examination of domestic violence not as discrete events, but as a continuous relational process unfolding across time.

### 2.5. Data Analysis and Conceptual Development

Data analysis followed an iterative, multi-stage process integrating inductive coding and abductive theoretical engagement.

#### 2.5.1. Open Coding and Pattern Identification

Interview transcripts were coded line-by-line to capture experiences, meanings, and relational processes. Codes reflected both explicit content and implicit meanings and were iteratively compared across cases to identify recurrent patterns. For details of the coding process please see Supplementary File S9 (Supplementary Methods).

Codes were grouped into subthemes and subsequently consolidated into five higher-order thematic domains:

(1) Experiences in the natal family;
(2) Relational dynamics within marriage and extended family structures;
(3) Coercive and unpredictable relational contexts resembling prolonged captivity;
(4) Psychological and somatic adaptations;
(5) Response and negotiation processes.

For details of the coding process please see Supplementary File S9 (Supplementary Methods).

#### 2.5.2. Analytic Transition to Conceptual Domains

A key analytic step involved reorganizing these descriptive themes into higher-order conceptual domains that capture underlying relational and developmental processes.

Themes that reflected overlapping mechanisms were analytically consolidated. In particular, themes relating to relational dynamics (Theme 3.2) and captivity-like conditions (Theme 3.3) were merged into a single domain of relational captivity, based on their shared emphasis on sustained constraint within relational systems. Themes 3.2 and 3.3 were analytically distinct but conceptually contiguous and were therefore integrated within a single SPECTRA domain of Relational Captivity. Theme 3.2 was derived primarily through inductive, open coding of participants’ accounts, capturing the lived structure of marital environments, including shifts from natal to marital homes, gendered role expectations, and the double burden of work and domestic life, and kinds of violence exposure. In contrast, Theme 3.3 was generated through higher-order, axial and abductive analytic processes, identifying patterned strategies of coercive control—such as threat, unpredictability, isolation, and emotional manipulation—that organized these environments over time. Drawing on multi-level coding (thematic, positional, processual, and interactional; see Supplementary File S9, Supplementary Table S3B), this theme elucidates the mechanisms through which control is enacted and internalized. Together, these themes describe complementary dimensions of the same relational phenomenon: Theme 3.2 delineates the conditions and contexts of constraint, while Theme 3.3 explicates the processes through which these conditions are stabilized into enduring systems of domination. Their integration within a single domain reflects this convergence and supports the conceptualization of domestic violence as a continuous, relationally embedded condition of captivity rather than a set of discrete acts.

Other themes were elevated into conceptual domains by focusing on the processes they represented. Experiences in the natal family were interpreted as reflecting early internalization of relational norms (developmental embedding), while psychological and somatic responses were conceptualized as adaptive responses to prolonged relational constraint. Patterns of response and resilience were interpreted as processes of negotiation and adaptive repair.

Through this process, five descriptive themes were reorganized into four interrelated conceptual domains:

- Developmental embedding
- Relational captivity
- Psychological and somatic adaptations
- Negotiation and adaptive repair

#### 2.5.3. Abductive Theoretical Engagement

Following inductive theme development, theoretical frameworks—particularly from complex trauma literature—were used as sensitizing constructs to refine interpretation. These constructs were not imposed as predefined categories but were used to examine points of convergence, divergence, and cultural specificity within participants’ narratives.

#### 2.5.4. Analytic Traceability

The progression from codes to subthemes, themes, and conceptual domains involved iterative clustering and abstraction. A detailed mapping of this process is provided in the Supplementary File S9 (Table S3A–D), which illustrates the stepwise analytic pathway from narrative data to the SPECTRA framework.

Throughout the Results, participant narratives were treated as analytic evidence rather than standalone testimony. Quotation boxes in numbered tables were therefore used selectively to substantiate interpretive claims, with all excerpts embedded within authorial analysis linking lived experience to thematic processes and the construction of the SPECTRA framework. Because Theme 3.3 is analytically organized around complex interactional, processual, and coercive relational mechanisms rather than discrete descriptive codes alone, multiple excerpts are used to make visible the unfolding dynamics of control, unpredictability, and captivity-like processes across cases. Similarly, Theme 3.5 draws on higher-order thematic, positional, and processual coding to capture shifting negotiations of agency, legitimacy, and adaptive repair over time; accordingly, multiple excerpts are included to illustrate these layered and temporally unfolding response processes. By contrast, Themes 3.1 and 3.2 rely more substantially on open and descriptive coding of developmental and relational contexts and therefore use fewer excerpts, retaining only those quotations that are analytically indispensable to demonstrating key conceptual transitions. No excerpts are included in Theme 3.4, as this section centers participants’ lived distress, bodily suffering, and psychological sequelae; to maintain a trauma-informed and ethically reflexive presentation, highly affect-laden quotations have been intentionally minimized to avoid sensationalizing narratives or introducing unnecessarily triggering material. For transparency, the corresponding interpretive elements—domain, central mechanism, and analytic relevance—for all boxed and in-text illustrative excerpts are systematically tabulated in Supplementary File S10, Table S22.

### 2.6. Rigor and Analytic Transparency

Methodological rigor was enhanced through:

- Independent coding by three researchers;
- Analytic memoing and reflexive journaling;
- Structured consensus discussions to resolve discrepancies;
- Cross-case comparison;
- Triangulation with field notes;
- Maintenance of an audit trail documenting coding and analytic decisions.

### 2.7. Researcher Positionality and Reflexivity

The research team comprises urban, educated Indian women with training in psychology, neuroscience, and qualitative methods. Our caste, class, and educational positions shaped both access to participants and interpretation of their narratives.

While we share cultural familiarity with participants’ contexts, we also occupy positions of relative structural privilege. Reflexive practices were used throughout the study to examine how these positions influenced analytic decisions.

The corresponding author’s prior engagement with relational trauma informed the conceptual orientation of the study. To mitigate interpretive bias, primary coding was conducted independently by multiple researchers, followed by reflexive group discussions and documentation of alternative interpretations where relevant. A more granular description of methods, ethical safeguards are provided in Supplementary File S9.

## 3. Results

**Figure 3:**
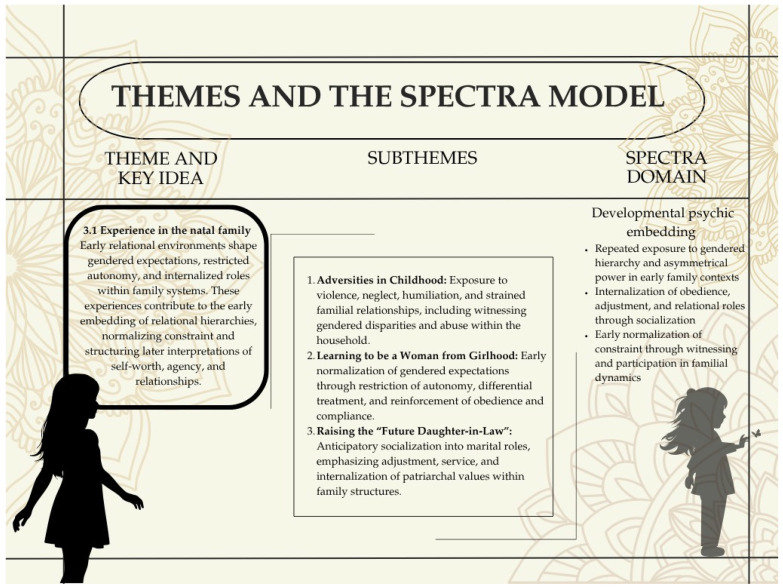
Developmental Psychic Embedding: Early Socialization and Internalization of Relational Hierarchies. This figure illustrates how experiences within natal families contribute to the early embedding of gendered relational hierarchies. Subthemes capture adversities in childhood, normalization of gendered expectations, and anticipatory socialization into marital roles, which together shape later interpretations of agency, self-worth, and relationships.

**Figure 4:**
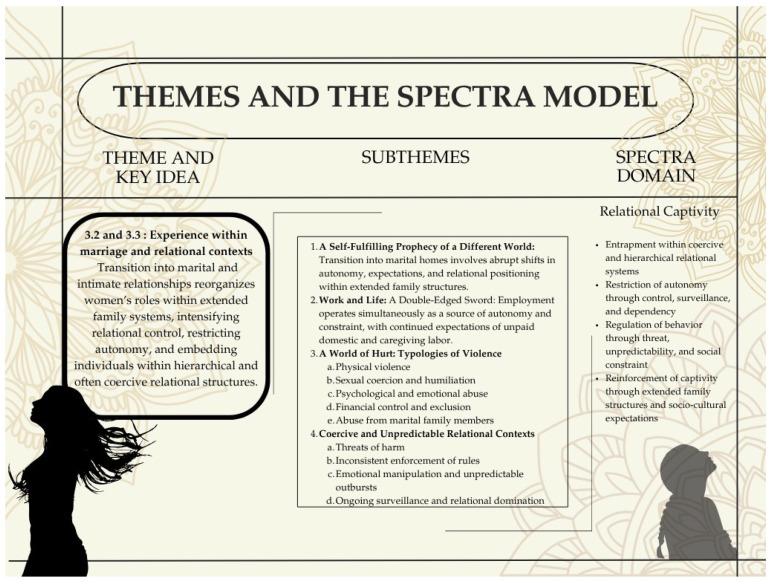
Relational Captivity: Control, Constraint, and Coercive Relational Systems. This figure shows how experiences within marital and extended family contexts consolidate into conditions of relational captivity. Subthemes reflect intensified control, multiple forms of violence, and environments characterized by threat, unpredictability, and restricted autonomy, highlighting the merger of relational dynamics and captivity-like conditions.

**Figure 5:**
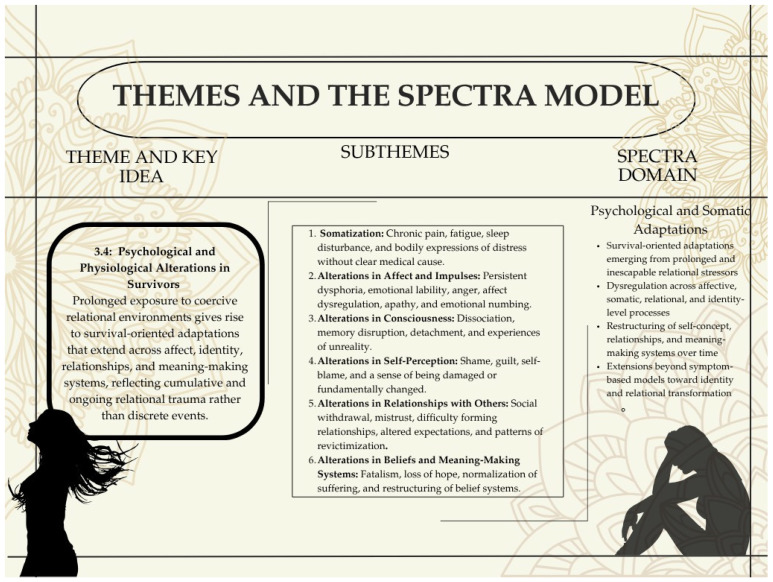
Psychological and Somatic Adaptations: Identity, Relational, and Meaning-System Changes. This figure depicts the range of survival-oriented adaptations emerging from prolonged relational constraint. Subthemes capture alterations across affect, somatic experience, consciousness, self-perception, relationships, and belief systems, reflecting cumulative and ongoing relational trauma.

**Figure 6:**
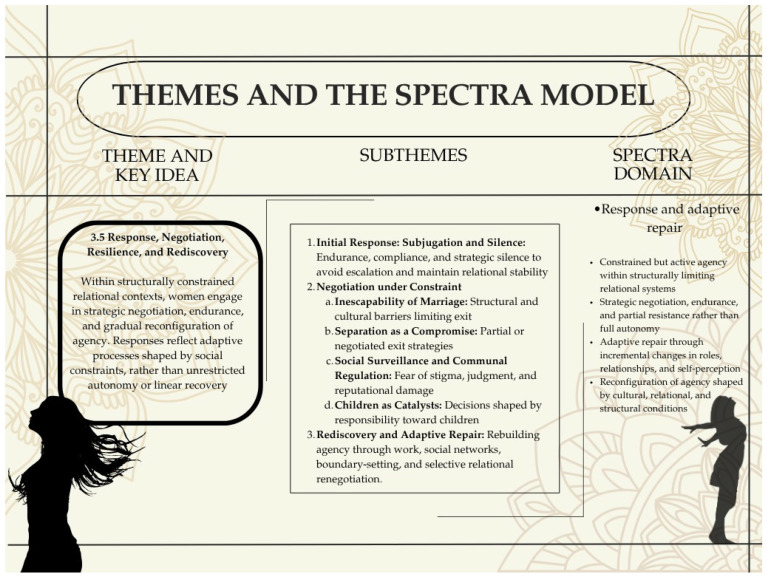
Negotiation and Adaptive Repair: Constrained Agency and Incremental Reconstruction. This figure illustrates how participants respond to relational constraint through processes of endurance, negotiation, and adaptive repair. Subthemes reflect initial subjugation, strategic negotiation under structural limits, and gradual reconfiguration of agency through relational and contextual shifts.

This section presents findings from narratives of ever-partnered, educated, urban Indian women, examining how they experience, understand, and negotiate violence within families and intimate relationships. The analysis draws on inductive coding and abductive engagement with theory, generating over 80 codes that were organized into subthemes and five overarching initial themes (Figure S1, Supplementary File S9). These were iteratively refined through abductive engagement with extant literature and intercoder agreements). This in turn led to five final themes that informed the four domains of the SPECTRA framework (Figure 2). We begin with brief case anchors to orient the reader to participants’ backgrounds and trajectories, followed by thematic sections (Themes 3.1–3.5), each structured around subthemes and supported by selected narrative excerpts, tables, and figures. Themes 3.1 and 3.2, 3.3 examine experiences in natal and marital contexts (Figure 3 and Figure 4), Theme 3.3 addresses coercive and captivity-like relational conditions (Figure 4), and Themes 3.4 and 3.5 focus on psychological and physiological adaptations (Figure 5) and processes of response and negotiation (Figure 6), respectively. Detailed case histories are provided in the Supplement. This section focuses on presenting empirical patterns and their immediate interpretation. Although thematic findings are linked to SPECTRA domains in the text and figures, the abductive analytic process is not detailed here; its logic is described in the Methods section and Supplement (Supplementary File S9 and Table S3A–D).

*Case Anchors*

Case Study 1: Ray is a 29-year-old PhD scholar who grew up in a violent and emotionally unstable household marked by fear, paternal aggression, and neglect, alongside later medical neglect around severe endometriosis.

In adulthood, she has moved through repeated intimate relationships marked by coercion, emotional abuse, and escalating vulnerability, with patterns of attachment and harm reinforcing each other across partners.

She remains in an unstable relationship she has struggled to leave, while continuing to experience significant psychological distress (For details, see Supplementary File S1).

Case Study 2: Damini is a 31-year-old PhD scholar in an inter-religious marriage who grew up in a conflict-ridden household, lost her father early, and took on financial responsibility from adolescence while caring for a mother with cancer.

Her trajectory includes sustained relational strain within her family of origin as well as significant conflict and distress within her marriage, alongside prolonged suppression of her own emotional needs.

A recent mental health crisis led her to seek psychiatric care for the first time, resulting in a PTSD diagnosis, and she is now engaged in therapy while attempting to stabilize both her mental health and her relationship (For details, see Supplementary File S2).

Case Study 3: Renu is a 31-year-old woman from Hyderabad who grew up in a supportive family but entered an arranged marriage that became severely abusive within two years.

Her marriage involved intense sexual violence, emotional abuse, and enforced isolation within a tightly controlled domestic environment.

With strong family support, she exited the marriage and is now rebuilding her life while managing trauma through therapy (For details, see Supplementary File S3).

Case Study 4: Priya is a 50-year-old teacher who spent years in an arranged marriage marked by coercive control, emotional abuse, and sexual coercion.

Her experience reflects chronic psychological domination structured through fear, guilt, and social pressure that constrained her ability to leave.

Now divorced and financially independent, she has rebuilt autonomy and no longer maintains contact with her former partner (For details, see Supplementary File S4).

Case Study 5: Meera is a 56-year-old teacher in a long-term marriage shaped by emotional neglect, a verbally abusive mother-in-law, and an often-absent husband.

Her experience reflects diffuse, chronic relational strain embedded in extended family dynamics rather than direct or acute partner violence.

She remains in the marriage but has disengaged emotionally, maintaining stability without expectations of change (For details, see Supplementary File S5).

Case Study 6: Maya is a 59-year-old school teacher who spent 17 years in a love marriage marked by sustained conflict and episodes of abuse.

Her experience reflects long-term relational conflict with normalized patterns of harm, where responsibility and blame were often shared or blurred over time.

Following separation, she achieved financial independence and now lives on her own while continuing to manage the emotional impact of the relationship (For details, see Supplementary File S6).

Case Study 7: Rhea is a 59-year-old teacher who entered an arranged marriage into a restrictive and conservative household that tightly controlled her mobility and autonomy.

Her marriage involved patriarchal control, emotional abuse, and sexual coercion within a highly regulated family structure.

Over time, she gained independence through employment, and the relationship has shifted into a more stable, though historically unequal, arrangement (For details, see Supplementary File S7).

### 3.1. Experience in the Natal Family

A scrutiny of these cases demonstrates that two of these women have experienced acts of abuse from their natal family as a child. Three of them have also witnessed their mother being subjected to violence or maltreated as a child themselves.

#### 3.1.1. Adversities in Childhood

Participants’ accounts indicate that childhood environments were shaped by both overt adversity and more subtle, enduring constraints on autonomy and expression. These early conditions appeared to structure later relational expectations and participants’ sense of agency.

Ray’s narrative reflects sustained exposure to violence and emotional instability within the natal family. She describes her childhood as *“quite terrifying,”* marked by her father’s extreme anger and unpredictable aggression. Although she initially turned to her mother for comfort, this relationship weakened over time, particularly after the birth of her brother, leaving a persistent absence of emotional safety and trust (See Supplementary File S1).

Damini’s experience similarly reflects cumulative adversity shaped by loss, instability, and premature responsibility. She describes strained familial relationships, financial hardship following her father’s death, and ongoing health and legal crises within the household. By age 17, she had assumed financial responsibility for the family, marking an early and developmentally significant transition into adult roles See Supplementary File S2. Table 2 shows some of their recollections of their childhood experiences. 

In contrast, four participants described childhoods as *“ideal”* or *“idyllic”*, emphasizing closeness and protection (See Supplementary Files S3–S7). Yet these accounts also revealed highly regulated environments in which opportunities—particularly education and career choices—were framed as permissions rather than entitlements. Across these narratives, autonomy remained constrained through prioritization of brothers’ education, restricted decision-making, and expectations of compliance.

Taken together, whether marked by overt adversity or regulated stability, participants’ childhood environments were structured by uneven power, constrained autonomy, and early expectations of adjustment. These conditions formed an important developmental substrate for how later relational harm was interpreted and endured.

**Table 2:** Illustrative excerpts from participants’ narratives capturing early experiences of childhood adversity within natal family environments (Also, see Table S4, Supplementary File S10).

Participant	Excerpt
Ray	*“My dad was the first one to abuse me psychologically, physically, in many ways since I was a small child”. “He used to hit me and my mother all the time in front of people, and demean us, always shout and criticise and never say anything nice”.*
Damini	*“My father died when I was 17, and my mother went into severe depression—she stopped speaking, stopped functioning, and turned hostile towards me, even saying I should have died instead. I was still in college, and we were suddenly facing major financial problems.”*

#### 3.1.2. Learning to Be a Woman from Girlhood: Normalization of Gendered Norms During Early Socialization

Participants’ accounts show that gendered expectations were normalized early within family and community contexts through everyday restrictions on mobility, peer interaction, and relational autonomy. These controls were often framed as protection, propriety, or necessity, making them appear natural within participants’ developmental environments.

Maya’s account illustrates how such regulation operated through both family control and wider community surveillance:

*“No, we were not allowed [to date]. My mother did not allow me to walk out, except with one of my best friends, only to her place. Nowhere else. Even in college we weren’t allowed to go out for movies or anything. You can go with your brothers and sisters, but not with friends.”*

This excerpt illustrates the Developmental Psychic Embedding domain by showing how restricted mobility and relational autonomy were normalized as part of gendered upbringing. The central mechanism here is early internalization of surveillance and constrained agency, where movement, friendship, and relational exploration were regulated differently for girls than for male family members. Analytically, this matters because such restrictions form an early relational script in which control is experienced as normative care, laying the developmental groundwork for later acceptance of control and adjustment within intimate relationships. For transparency, the corresponding interpretive elements—domain, central mechanism, and analytic relevance—for all boxed and in-text illustrative excerpts are systematically tabulated in Supplementary Table S21.

Several participants further described these restrictions as extending beyond the household into broader community monitoring, where reputation and family honor were collectively enforced. These early experiences positioned marriage as an expected and inevitable transition, with little space for autonomous relational decision-making prior to it.

Together, these narratives suggest that gendered norms were embedded not only through explicit familial rules but also through the structuring of everyday life itself—making restriction, supervision, and anticipatory compliance appear ordinary rather than coercive.

#### 3.1.3. Raising the Perfect Future Daughter-in-Law, Rather than a Daughter—The Tyranny of “Adjustment”

Participants’ narratives indicate that gendered socialization extended beyond restriction to active preparation for future marital roles. From an early age, they were shaped into what families considered ideal future daughters-in-law, with emphasis on adjustment, endurance, and prioritizing others over oneself.

Across accounts, “adjustment” emerged as a central expectation structuring behavior, emotional expression, and responses to difficulty. Conveyed through everyday instruction, observation of older women, and repeated reinforcement within family interactions, it was framed not as a choice but as an essential quality of a “good” woman, closely tied to family reputation and marital success.

These expectations also shaped aspirations and life choices, often constraining educational and occupational trajectories in anticipation of marriage (see Table 3). Rhea’s account shows how career aspirations were actively redirected to align with gendered respectability and class norms, while Priya’s narrative reflects how limited ambition was normalized, with marriage positioned as a sufficient life outcome.

In several cases, this involved suppression of personal preferences, emotional self-management, and anticipatory accommodation to future marital households. Participants described learning to avoid confrontation, tolerate discomfort, and maintain harmony even at personal cost.

Importantly, this socialization was not always experienced as overt coercion in childhood. Rather, compliance, sacrifice, and endurance were gradually internalized as markers of maturity and virtue, producing a framework in which distress was expected to be managed through further adjustment rather than resistance.

Together, these accounts suggest that participants were actively shaped in anticipation of future marital roles. The normalization of “adjustment” functioned as a key developmental mechanism through which gendered expectations were internalized, shaping later responses to relational adversity.

**Table 3:** Illustrative excerpts on gendered shaping of aspirations and adjustment expectations (Also see Supplementary File S10 and Table S5).

Participant	Excerpt
Rhea	*“I wanted to become a nurse, inspired by Florence Nightingale, but my family objected—it wasn’t considered a respectable profession for girls in our family. I was steered into teaching instead, and daughters from middle class homes were not encouraged to enter the family business.”* *“We were brought up that way only that when girls get married, you have to adjust yourself with your in laws and you have to set yourself in the ways of the family and accept whatever they say”*
Priya	*“Ambition…not really. I wasn’t much ambitious, I would say. You know normal typical Indian—if I’d get a job, fine. Otherwise, get married and get settled”*

Theme 3.1 maps onto the SPECTRA domain of Developmental Psychic Embedding by showing how early familial environments shape the internal conditions through which later relational experiences are interpreted and negotiated. Findings from this theme indicate that childhoods—whether marked by overt adversity or more subtle restriction and gendered socialization—were structured by expectations of compliance, adjustment, and constrained autonomy. These experiences were developmentally embedded rather than episodic, shaping participants’ sense of self, relational expectations, and thresholds for recognizing harm.

As elaborated in the analytic trace (Table S3A, Supplementary Methods), these patterns are reflected across thematic, positional, and process-level codes, indicating that later experiences within intimate relationships are not discrete ruptures but unfold along developmental trajectories established in childhood. This domain therefore captures how early relational adaptations become internalized and carried forward, forming the developmental substrate for later relational captivity.

### 3.2. Experience Within Marriage and/or Relationships: Relational Control Within Extended Family Structures

Participants’ narratives show that experiences within marriage and intimate relationships were shaped by deeply embedded patriarchal norms enacted not only by partners but also through extended family structures. While physical and sexual violence were more readily recognized, participants described a broader range of experiences—including coercion, emotional abuse, economic restriction, and constraints on autonomy—that were often normalized and not always identified as violence.

These patterns did not emerge in isolation within marital homes but were continuous with earlier socialization, where expectations of adjustment and compliance had already been established, albeit in less explicit forms. Within marital families, however, these norms became more rigidly enforced, often by multiple family members, structuring women’s everyday lives as newly incorporated daughters-in-law.

#### 3.2.1. A Self-Fulfilling Prophecy of a Fundamentally Different World in the Marital Home

All married participants described their marital homes as fundamentally different from the environments in which they had grown up, often experiencing them as more restrictive and regressive. For several participants whose childhoods had been highly sheltered and closely regulated, marriage marked their first sustained encounter with coercion, hostility, and the harsher realities of adult relational life. This transition was often experienced with shock, disbelief, and a profound sense of being unprepared for the intensity of control and emotional demands they encountered. Expectations around who they were, what they could do, and how they should live shifted abruptly after marriage, affecting work, social connections, and everyday autonomy.

Rhea’s narrative illustrates this transition sharply. Prior to marriage, she was employed, socially engaged, and pursuing multiple interests, including teaching and music. Following marriage, however, she entered a highly conservative household where she was not permitted to work, and her life became confined to domestic roles for over a decade. This shift extended beyond employment to her social world and sense of self. Her accounts of restricted communication with her family—through monitored phone calls, difficulty sending letters, and lack of privacy—reflect a broader pattern of isolation and control within the marital home (See Table 4, Supplementary File S7).

Priya similarly described a life marked by total control, with no independent social life and a pronounced disconnection from her past. Across these cases, marriage marked not a continuation of earlier life trajectories, but a rupture that redefined identity, autonomy, and relational boundaries.

Restrictions around employment emerged as a key mechanism of control. Several participants described being prevented from continuing their jobs after marriage, often without prior awareness. Renu, for instance, was required to resign from her position and relinquish her academic work, effectively confining her to the home (See Supplementary File S3). In contrast, Meera was allowed to work because her income was needed; however, this did not translate into autonomy. She remained embedded in a volatile domestic environment, managing an unpredictable and at times abusive mother-in-law, navigating social ostracism, and sustaining a strained marital relationship while effectively functioning as a single working parent. Her account reflects how employment could coexist with, rather than mitigate, relational constraint.

Damini’s circumstances differed in that she lived separately from her in-laws and was employed at the time of marriage. However, she continued to face emotional hostility from her marital relatives, particularly in response to her court marriage, which violated family expectations. Their exclusion of her mother from the wedding reception led to a prolonged relational rupture, highlighting how control could operate even in the absence of co-residence.

Taken together, these accounts suggest that marriage often enacted a self-fulfilling transition into a more controlled and restrictive world, where earlier norms of adjustment and compliance were intensified and enforced within extended family structures. These shifts were not always framed as violence, but were experienced through sustained restrictions on autonomy, mobility, work, and social connection.

**Table 4:** Illustrative excerpt on fundamentally different marital home and life (Also, see Supplementary File S10, Table S6).

Participant	Excerpt
Rhea	*“I had just started my career—teaching, doing my master’s, even singing Hindustani classical music—and I was very happy then. But after my arranged marriage into a conservative family, everything changed. I was not allowed to work, and my career stopped. For fifteen years, I remained a housewife.”*

#### 3.2.2. Work and Life: A Double-Edged Sword

Across participants, marital homes were particularly different in how women’s roles were reorganized around domestic labor and caregiving. Even when women were educated and employed, their identities as wives and daughters-in-law remained closely tied to managing the household, often without recognition or support.

For those who continued working or returned to work after marriage (Meera, Rhea, Maya, Priya), employment did not reduce these expectations. Instead, it was layered onto existing responsibilities, creating a persistent double burden. Meera’s account captures this tension: although her employment was enabled by the family’s need for income, she remained solely responsible for caregiving, including managing an unpredictable and at times abusive mother-in-law, while navigating ongoing conflict with a husband who did not acknowledge any need for shared responsibility (See Supplementary Files S4–S7).

This pattern recurred across cases in different forms. Women described managing childcare, household work, and emotional care largely on their own, even when husbands were absent, disengaged, or emotionally unavailable. There was little expectation—either from partners or from the family system—that these responsibilities would be shared. Instead, the capacity to manage everything simultaneously was treated as an expected and unremarkable part of being a wife.

In some cases, work offered mobility or continuity with earlier identities. Yet it did not fundamentally alter the structure of everyday life. Domestic labor continued to dominate time and attention, with participants describing days shaped by constant task management across household and professional domains.

Importantly, this reflects a broader social pattern particularly pronounced in South Asian contexts. Participants described a setting in which employment did not redistribute expectations within the home; women were expected to work while continuing to bear the full burden of unpaid labor, while men’s roles remained largely unchanged.

Across cases, this produced a sustained tension between obligation and self-fulfillment. Employment did not necessarily offer relief; instead, it often intensified the strain of negotiating multiple, non-negotiable roles, with limited space for rest, recognition, or reciprocity.

#### 3.2.3. A World of Hurt: Experience of Several Typologies of Violence from Romantic Partners and Husbands and Their Families

Participants’ accounts revealed patterned experiences of violence that unfolded over time across multiple, often co-occurring forms and frequently intensified within relationships. While physical violence was present in several cases, sustained emotional, sexual, and psychological harms more consistently shaped participants’ everyday lives. Narratives described cycles of abuse marked by escalation, intermittent periods of relative calm, and repeated attempts to make sense of or endure partners’ behavior, often accompanied by guilt, shame, and humiliation (See Supplementary Table S7).

##### Physical Violence

Physical violence was reported by several participants (Ray, Renu, Maya), typically in the form of hitting, slapping, pushing, or physical overpowering. Although not always frequent, these incidents were severe, unpredictable, and embedded within ongoing emotional abuse.

Renu experienced repeated episodes of physical violence within a short span of marriage. Ray described violence across multiple relationships, beginning with her first partner and continuing into later ones, with increasing intensity and fear. In one instance, she narrowly escaped serious harm; in another, she experienced direct physical restraint and choking. Maya’s experiences were closely tied to her husband’s alcohol use, with brutal beating and assaults directed at both her and their child. Notably, the violence did not fully cease even after separation.

Across these cases, physical violence was embedded within broader patterns of fear, control, and emotional degradation rather than occurring as isolated incidents.

##### Sexual Violence, Coercion, Deprivation and Humiliation

A majority of participants (Ray, Renu, Rhea, Damini, Priya) reported experiences of sexual coercion or non-consensual sexual encounters within their relationships. These experiences were often difficult to articulate, and participants were at times guarded or reluctant to elaborate but consistently acknowledged them as distressing and violating.

For some, coercion involved force or pressure in moments of resistance; for others, it took the form of manipulation, intoxication, or situations in which refusal was not meaningfully possible. In several cases, sexual intimacy itself became a site of humiliation and control, where consent was undermined through both coercion and emotional degradation during or after the act. Over time, these experiences produced withdrawal from intimacy and fear or aversion associated with sexual contact.

Among older participants, responses were often more restrained and marked by resignation rather than detailed narration. Some described limited agency in decisions around sexual activity and contraception, while others linked the absence of intimacy to emotional estrangement within marriage. In these cases, sexual relationships were shaped less by mutuality or desire than by obligation, power imbalance, and emotional disconnection.

##### Psychological Abuse and Violence

Psychological abuse was the most pervasive and enduring form of violence across all participants’ narratives. It appeared in sustained patterns of humiliation, verbal aggression, neglect, exclusion, and emotional manipulation, often continuing over years or decades.

For participants such as Ray and Damini, repeated involvement in abusive relationships reflected cycles of emotional manipulation, abandonment, threats, and systematic undermining of self-worth across multiple partners. For married participants, psychological abuse was deeply embedded within family dynamics, particularly through the structurally vulnerable position of the daughter-in-law within the marital household.

Renu described persistent humiliation and exclusion within her marital home, where she was actively silenced and treated as inferior, with little intervention from in-laws despite clear awareness. Priya similarly described escalating verbal abuse, public humiliation, financial restriction, and emotional neglect. Maya’s account reflected emotional abuse, financial neglect, and instability linked to her husband’s substance use, producing chronic anxiety and insecurity over time.

For Meera and Rhea, the impact of psychological abuse unfolded over longer durations. Both described years of verbal humiliation, strained relationships with in-laws, and emotional distance from their husbands. In Meera’s case, this was compounded by caregiving demands and social isolation, contributing to significant deterioration in both physical and mental health. Rhea similarly described enduring humiliation and emotional suppression, with limited space to express distress or resist. The treatment of her recently widowed mother (Table 5) further illustrates how gendered norms regulating women’s bodies, behavior, and respectability extended beyond the individual to shape family interactions across generations.

Across cases, psychological abuse was often narrated in a matter-of-fact manner, reflecting the extent to which these experiences had become normalized. Yet its cumulative impact was profound, shaping participants’ self-concept, emotional well-being, and capacity for relational trust over time. Related excerpts are further detailed in Supplementary Tables S6 and S7A.

**Table 5:** Cultural regulation of women’s dignity and respectability (Also, See Supplementary File S10, Table S7A).

Participant	Excerpt
Rhea	“I had offered her a cake… and immediately I got a comment… she’s a widow and she’s having cake *… When she wore a coloured blouse, they said, ‘a widow wearing a coloured blouse *’… The way they treated her… it was a nightmare for me.” * Traditionally, among Indian Hindus, widows are prohibited from wearing any color other than white, as well as from eating any kind of non-vegetarian food, including eggs, which are used in baking cakes. They are given bland and vegetarian food only, prohibited and ostracized from social celebrations like marriages, or festive occasions

### 3.3. Coercive and Unpredictable Relational Contexts Resembling Prolonged Captivity

The patterns described in Section 3.2 extend beyond discrete acts of violence into a more pervasive relational environment characterized by control, unpredictability, and psychological domination. This section focuses on how these dynamics converge into conditions resembling prolonged captivity, where the objective is not only compliance but also the gradual reshaping of the survivor’s perceptions, responses, and sense of self. This closely aligns with Herman’s formulation of captivity, in which sustained coercive control produces not only obedience but also a psychologically dominated subject who adapts to the perpetrator’s demands, at times willingly and even gratefully.

Across participants, these environments were marked by sustained exposure to coercive strategies that produced fear, dependence, and disorientation. Rather than isolated incidents, these dynamics operated as ongoing relational conditions, exercised through threat, unpredictability, manipulation, and isolation. Over time, participants described adapting by anticipating reactions, modifying behavior, and suppressing resistance in ways that increasingly aligned with perpetrator expectations (See Supplementary File S10, Table S8).

#### 3.3.1. Threats of Harm

Threats—direct or implied—were a recurring feature across participants’ accounts and often extended beyond the individual to include family members. Ray and Damini described threats directed at themselves and their mothers, creating a wider field of fear that shaped responses within relationships. For Maya, her husband’s violence, particularly when intoxicated, produced a constant state of vigilance in which concern for her own safety was inseparable from fear for her children. Meera similarly described living with an unpredictable and at times dangerous mother-in-law, where the possibility of harm remained an ongoing concern.

These experiences illustrate how fear moved beyond specific incidents to become an organizing condition of everyday life, reinforcing compliance and discouraging resistance.

#### 3.3.2. Inconsistent Enforcement of Trivial Demands and Petty Rules

Participants described living within relational environments governed by arbitrary and shifting rules, where expectations were unclear, inconsistently applied, and often enforced with aggression. Table 6, Table 7, Table 8, Table 9 and Table 10 show participants’ excerpts illustrating such events in their lives.

Ray’s account of her partner imposing changing and inexplicable rules around communication reflects this pattern, where even minor perceived violations could trigger disproportionate reactions.

**Table 6:** Capricious rule enforcement and unpredictability as mechanisms of control (See Supplementary File S10, Table S9).

Participant	Quote
Ray	*“Everything is a rule—and he keeps changing them. Even if I follow all his rules, he creates new ones after the fact and blames me. It’s impossible to keep up. One moment he says he loves me, and the next, in the same breath, says I’m nobody.”*

Across cases, this unpredictability extended to everyday interactions, including domestic tasks and routine behavior. Participants described having to anticipate and manage potential triggers, often describing a constant need to monitor their own actions to avoid conflict. This produced a state of ongoing uncertainty, where stability was contingent on aligning with expectations that were neither fixed nor transparent.

Such conditions contributed to a heightened sense of vigilance, where participants adjusted their behavior pre-emptively, often at significant emotional cost.

#### 3.3.3. Emotional Manipulation and Unpredictable Violent Outbursts

Emotional manipulation emerged as a central mechanism through which control was maintained. Participants described gaslighting, humiliation, threats of self-harm by partners, and systematic undermining of confidence, appearance, and self-worth. For Ray and Damini, these patterns recurred across multiple relationships, combining emotional degradation with intimidation and control. Ray specifically describes Mohsin as being intentionally hurtful and degrading, including mockery of her illness.

**Table 7:** Humiliation and identity-based degradation as strategies of emotional manipulation and control (See Supplementary File S10, Table S10).

Participant	Quote
Ray (29)	*“He will say very, very hurtful things, very abusive things. Very loathsome things, very disgusting things. Very violent things…. He is always insulting me. He makes fun of my problems. He makes fun of everything I believe in. He makes fun of my disease even. He has called me an autistic retarded bitch. ‘I can’t deal with you anymore. You and your autism can go to hell’”.*
Damini (31)	*“I was told my English is not good… if I pronounce this word like this in front of a public gathering I would be laughed at. Somebody had once told me I look like a bhains (buffalo). That my hands are not good, my feet are not beautiful”*

In several cases, perpetrators constructed narratives that destabilized participants’ sense of reality, including false claims, exaggerated threats, and deliberate distortion of events. Damini described prolonged periods of fear induced by threats involving her family, while also being subjected to ridicule targeting her appearance, dark complexion, accented English, and socioeconomic background. These experiences contributed to a gradual erosion of self-confidence and increased dependence on the perpetrator.

Participants also described episodes of extreme and unpredictable anger, often disproportionate to the situation. Renu’s experiences illustrate how minor domestic lapses could escalate into aggressive confrontations, including threats of eviction and public humiliation. Such incidents extended the site of control beyond the private sphere, embedding it within broader social contexts of honor and respectability.

**Table 8:** Unpredictable and disproportionately violent outbursts of anger as strategies to foster uncertainty and constant fear (See Supplementary File S10, Table S11).

Participant	Excerpt
Renu	*“He would go into a rage over small things. Once, when I forgot to set his alarm after waking up at 4 am to cook, he dragged me out of the house and threatened to throw me out. This happened multiple times—over minor mistakes, he would threaten eviction or throw away the food I had cooked.”*
Ray	*“When he loses his temper, he becomes like a monster… anything can trigger it. Even if I follow all his rules—when to call, how long to speak—he still finds something to get angry about, abuses me, threatens me, or cuts me off.”*

Acts of intimidation, including destruction of belongings and aggressive outbursts, further reinforced this environment. Maya and Priya both described partners who engaged in such behavior during episodes of anger, contributing to an atmosphere of instability and fear.

Isolation functioned as a critical component of this system. Participants described being cut off from communication, prevented from maintaining contact with friends and family, or placed in situations where social interaction became difficult or unsafe. Renu’s experience of repeated disruption of her communication devices and Maya’s inability to host or maintain social connections illustrate how access to external support was systematically restricted.

**Table 9:** Isolation through control of communication and enforced disconnection from support systems (See Supplementary File S10, Table S12).

Participant	Excerpt
Renu	*“Whenever my phone rang, he would get angry. I tried to take calls in the kitchen where others were present because it felt safer. But he often broke my phone when I spoke to my parents. Over two years, I went through several phones because they kept getting destroyed—just to make sure I stayed cut off from the outside world.”*

Public humiliation also emerged as a powerful tool of control. Renu described being dragged out of the house by her husband, verbally berating and threatening to evict her because of an alarm she forgot to set. She was also shamed by her father-in-law in a public setting, reinforcing both familial authority and social surveillance. This account illustrates how control is enacted not only through private coercion but also through public humiliation, where social norms of respectability and shame are mobilized to enforce compliance and suppress resistance. These episodes were often chaotic and intentionally destabilizing, reinforcing a sense that safety was contingent on perfect compliance.

**Table 10:** Intentional gaslighting and public humiliation and control as mechanisms of compliance (See Supplementary File S10, Table S13).

Participant	Quote
Renu	*“He dragged me outside the house… and threatened to throw me out… I ran out just to make a phone call… and my father-in-law shouted at me in front of neighbours… saying I was dressed inappropriately… like a loose woman.”*
Ray	*“He told me he knows exactly how to hurt me—and that he will keep doing it, even step it up, to destroy me emotionally.”*

Across these accounts, control was not maintained through a single form of abuse but through the convergence of multiple strategies that reinforced one another. Participants described gradually adapting to these conditions—monitoring their behavior, suppressing reactions, and attempting to anticipate outcomes—often leading to a narrowing of their social world and increasing dependence on the very relationships that were causing harm. These converging strategies of coercive control across participants are further illustrated through additional excerpts in Supplementary Tables S8–S13.

##### Mapping Themes 3.2 and 3.3 onto the SPECTRA Domain of Relational Captivity

Themes 3.2 and 3.3 capture complementary dimensions of relational control, with Theme 3.2 describing the structural conditions of constraint within marital environments and Theme 3.3 elucidating the processes through which these conditions are enacted and sustained. Together, they are integrated within the SPECTRA domain of relational captivity, reflecting domestic violence as a continuous and relationally embedded condition rather than a set of discrete acts.

### 3.4. Psychological and Physiological Alterations in Survivors: Loosely Aligned with Herman’s Prediction of Sequelae in Survivors of Prolonged Victimization

Participants’ narratives revealed a range of psychological and physiological alterations that developed over time in the context of prolonged relational trauma. These changes were described not as discrete symptoms but as enduring shifts in emotional states, bodily experiences, self-perception, and relational functioning. While these empirically derived patterns are broadly consistent with Herman’s formulation of the sequelae of prolonged victimization, they are presented here as lived adaptations rather than diagnostic categories (See also Supplemental Table S1). For analytic clarity, the conceptual organization of these alterations is summarized in Table 11, while the results below focus on how these patterns are expressed across participants’ experiences (see also Table S3C, Methods and Supplementary Methods). Excerpts from participants’ narratives across subthemes are tabulated in Supplementary Table S14.

#### 3.4.1. Somatization (Physical Manifestations of Trauma)

Participants described a range of physical health changes that developed over the course of prolonged exposure to relational stress and abuse, often without clear medical explanation or with worsening of existing conditions. These manifestations were not reported as isolated symptoms but as persistent bodily states that participants directly associated with their relational environments.

Several participants linked sustained psychological distress to significant physical health outcomes. Meera described severe and prolonged emotional strain in her marital home, particularly in relation to conflict with her mother-in-law and lack of support from her husband, which she associated with the onset of a serious cardiac condition. Maya similarly reported a marked worsening of her diabetes during the years of her marriage, which she connected to ongoing stress and instability.

Others described more diffuse but persistent bodily changes. Priya experienced significant weight loss, along with disturbances in sleep and appetite during periods of sustained conflict, at times requiring medication for insomnia. Ray reported chronic gynecological conditions that were exacerbated over time, alongside ongoing fatigue, musculoskeletal pain, and sleep disruption. In her account, physical and psychological distress appeared to reinforce one another, producing a cycle of worsening symptoms.

Across participants, these experiences point to a pattern in which prolonged exposure to relational violence and control becomes embodied over time, shaping not only emotional states but also physical functioning. These patterns are reflected in somatization-related code clusters within the analytic framework (see Table S3C) and are interpreted here as cumulative bodily adaptations to sustained stress rather than discrete medical conditions.

#### 3.4.2. Alterations in Affect and Impulses

Participants described pervasive and long-standing alterations in emotional states and regulation, often emerging gradually over the course of prolonged exposure to relational violence. These changes were not confined to specific episodes but became enduring patterns shaping how participants experienced, expressed, and managed affect. Across cases, participants oscillated between heightened emotional reactivity and emotional constriction, with significant implications for decision-making, self-regulation, and interpersonal functioning (see Table S3C, Methods).

##### Persistent Dysphoria

Many participants described sustained states of emotional distress characterized by anxiety, low mood, restlessness, and intrusive recollections of past experiences. These were often long-lasting and, in several cases, persisted even after leaving abusive environments.

Ray and Renu reported heightened sensitivity to external stimuli, ongoing anxiety, and intrusive recollections that were triggered in everyday contexts, such as being alone or navigating unfamiliar spaces. These experiences interfered with sleep, concentration, and a sense of safety. Renu, in particular, described lingering fears and startle responses that continued years after separation.

Depressive features were also evident across participants. Damini described prolonged periods of emotional overwhelm, panic episodes, and difficulty maintaining routine functioning, which eventually led her to seek professional help. Ray similarly reported enduring struggles with anxiety, low confidence, and persistent emotional distress. Among older participants, these states were less explicitly labeled but were reflected in descriptions of prolonged emotional strain, fatigue, and distress linked to past experiences.

##### Anger and Emotional Lability

Participants described significant difficulties with anger, both in terms of suppression and dysregulated expression. In many cases, anger could not be directed toward perpetrators due to fear of escalation, leading to its internalization or displacement.

Meera reported episodes where accumulated distress resulted in breaking household objects, particularly when conflict within the home intensified. Maya described frequent anger that required active suppression to maintain stability within the household. Damini noted that conflicts within her marriage sometimes escalated to the point where both partners directed harm toward themselves rather than toward each other.

Ray described a long-standing difficulty in managing anger, where attempts to regulate emotional responses were often unsuccessful, and external attempts to calm her could further intensify her reactions. Across participants, anger appeared both as a suppressed and dysregulated affect, reflecting the constraints within which it was experienced.

##### Affect Dysregulation, Impulsivity

Several participants described difficulties in regulating emotional responses, with reactions that were experienced as disproportionate or difficult to control. Maya noted that she continued to become easily overwhelmed and found it difficult to regain emotional equilibrium. Renu described involuntary emotional responses, such as crying despite attempts to remain composed, particularly when distressed.

These patterns were often accompanied by difficulties in managing stress, leading participants to seek immediate coping mechanisms, such as reaching out to family members during moments of overwhelm. Across cases, emotional responses were experienced as both intrusive and difficult to regulate, indicating enduring disruptions in affective control.

##### Apathy and Numbness

In contrast to heightened emotional states, participants also described periods of emotional numbing and detachment, often emerging after prolonged exposure to distress. Ray described reaching a point where emotional disengagement became necessary to tolerate ongoing experiences, indicating a shift toward psychological withdrawal as a means of coping.

Among older participants, similar patterns were reflected in subdued emotional expression and a tendency to describe distress in minimal or indirect terms. Participants such as Maya and Priya often spoke about their experiences in a restrained manner, with limited elaboration, and emphasized endurance rather than emotional expression. These patterns suggest a form of emotional constriction that developed over time in response to sustained exposure to distress.

##### Hopelessness and Suicidal Ideation

In more severe cases, participants described experiences of profound hopelessness and thoughts related to self-harm. Ray and Damini reported periods marked by intense despair, where emotional distress was accompanied by thoughts of ending their lives.

These experiences were often linked to cumulative exposure to both early adversity and later relational trauma, combined with feelings of isolation and lack of support. In these cases, distress was not episodic but persistent, contributing to a diminished sense of future orientation and a perception of limited alternatives.

##### Inhibited Affect

Several participants, particularly among the older group, displayed patterns of inhibited affect, especially when describing earlier phases of their marriages. Experiences of humiliation, coercion, and restriction were often narrated with minimal emotional expression, reflecting long-standing patterns of suppression.

Participants such as Maya, Priya, and Rhea described enduring difficult circumstances with limited overt resistance, often emphasizing adjustment and endurance. This pattern appeared to be shaped both by early socialization and by the consequences of expressing dissent within abusive environments, where resistance often led to escalation.

Even in the present, participants described continuing to internalize distress and avoid sharing emotional experiences with others. Maya and Priya, for instance, reported having few avenues to express their concerns, often managing stress privately. In interviews, their responses were initially brief and restrained, with more detailed accounts emerging only with sustained engagement.

Across cases, inhibited affect appeared as an enduring adaptation, reflecting both learned suppression and the long-term consequences of environments where emotional expression was constrained.

#### 3.4.3. Alterations in Consciousness

Participants described disruptions in attention, memory, and states of awareness that emerged in the context of prolonged exposure to trauma. These alterations were reflected in experiences of dissociation, fragmented recall, and difficulties in sustaining attention, often occurring during or in relation to distressing memories.

Ray described recurrent episodes of detachment, particularly in earlier periods of her life, where she experienced a sense of observing herself from a distance. These experiences appeared to function as a way of disengaging from overwhelming situations, especially in the context of early exposure to abuse. Maya reported similar patterns of detachment, describing frequent states of disconnection that were not tied to a single event but occurred repeatedly over time.

Disturbances in memory were also evident. Participants described difficulty recalling specific episodes of abuse or providing coherent accounts of events, even when the emotional intensity of those experiences remained strong. Maya, for instance, demonstrated marked gaps in recall when asked to describe particular instances, while Ray showed variability in recall across different relational contexts, with some experiences remembered in fragmented detail and others remaining indistinct.

Participants also exhibited disruptions in attention and continuity of thought during interviews, including losing track of conversations and requiring prompts to re-engage. These patterns suggest difficulties in concentration and sustained cognitive engagement that extend beyond the immediate context of trauma recall.

In some cases, alterations extended to a disrupted sense of time and future orientation. Ray described increasing difficulty tracking time and maintaining engagement with academic and work-related responsibilities, alongside an absence of clear future-oriented thinking. Across participants, these alterations reflect enduring changes in how experiences are processed, recalled, and organized, as captured in consciousness-related code clusters within the analytic framework (see Table S3C, Methods).

#### 3.4.4. Alterations in Self-Perception and Disorganization in Personality

Participants described profound shifts in how they perceived themselves over time, often marked by diminished agency, internalized blame, and a sense of being fundamentally altered by their experiences. These changes were not limited to emotional responses but extended to the structure of identity and self-evaluation, shaping how participants understood their past, present, and future selves. The conceptual organization of these alterations in self-perception is summarized in Table 12, while the patterns described below reflect their empirical expression in participants’ narratives.

##### Shame and Guilt

Feelings of shame and guilt were pervasive across participants, often linked to experiences of loss of control within relationships and the internalization of blame for the abuse they endured. Participants described gradually coming to see themselves as responsible for conflict or violence, particularly in contexts where resistance led to escalation or further harm.

Ray articulated a persistent sense of limited control over her life, attributing both her circumstances and her inability to leave abusive relationships to factors she perceived as personal limitations. Priya similarly described being made to feel responsible for her husband’s behavior, often blaming herself for actions that she retrospectively recognized as unjustified. Even after separation, she continued to experience guilt associated with leaving the marriage.

Damini traced her feelings of guilt to both her early family responsibilities and later relational experiences, describing difficulty in prioritizing her own needs without experiencing discomfort. Among older participants, such as Maya and Rhea, guilt was often intertwined with early socialization into roles emphasizing obedience and adjustment, leading to the normalization of endurance and self-blame. Across cases, these patterns suggest that shame and guilt were not incidental but became embedded in participants’ self-concepts over time (see Table S3C).

##### Seeing Self as Damaged and Fundamentally Altered

Participants frequently described a sense of being changed in enduring and often irreversible ways. This was reflected in diminished self-worth, loss of confidence, and difficulty recognizing or reconnecting with earlier versions of themselves.

Ray described a loss of purpose and uncertainty about her own identity, alongside pervasive self-consciousness and heightened concern about how she was perceived by others. Maya reported longstanding feelings of inadequacy linked to disrupted educational and personal aspirations. Renu described a marked transformation in her personality following her separation, noting difficulties with concentration, avoidance of confrontation, and a sense that her earlier, more assertive self was no longer accessible.

Participants also described changes in how they related to their own bodies and appearance, often shaped by earlier experiences of criticism and humiliation. Damini linked repeated derogatory comments about her appearance—both in childhood and in later relationships—to enduring insecurities and a heightened vulnerability to seeking validation within harmful relational contexts.

Across participants, these accounts reflect not only diminished self-evaluation but also a broader disorganization in identity, where experiences of prolonged control and abuse reshaped how individuals understood themselves and their place in relationships and the world. These patterns align with self-perception–related code clusters within the analytic framework (see Table S3C).

#### 3.4.5. Alterations in Relationship with the Perpetrator

Participants described complex and often contradictory relational dynamics with their abusive partners, shaped over time by cycles of control, intermittent affection, and psychological dependence. These relationships were not experienced as uniformly coercive but were marked by alternating phases that reinforced attachment while sustaining harm.

Several participants described remaining in or returning to abusive relationships despite recognizing their harmful nature. Ray, for instance, continued to engage with her partner in anticipation of moments of care and emotional connection, which appeared to outweigh, or temporarily obscure, the cumulative impact of abuse. This pattern reflects a form of relational dependence in which intermittent positive reinforcement sustains engagement within otherwise damaging relationships.

Minimization of abuse was also evident across participants. Ray frequently downplayed the severity of her partners’ behavior, particularly in romantic relationships, even when describing sustained emotional and psychological harm. Among older participants, similar patterns emerged in different forms. Maya contextualized her husband’s violence and infidelity as contingent on external factors such as alcohol use, while Rhea and Meera interpreted or normalized their experiences through prevailing social expectations, framing them as typical or unavoidable aspects of marriage.

Participants also described adapting their interpretations of events to maintain relational stability. Meera, for example, coped with persistent conflict by withdrawing and avoiding confrontation, while simultaneously downplaying the severity of the situation. Across cases, such responses appeared to function as mechanisms for managing ongoing distress within relationships that could not be easily exited.

These patterns suggest that relationships with perpetrators were not only sites of harm but also contexts in which participants developed adaptive strategies to endure, interpret, and sustain those relationships. These dynamics are reflected in perpetrator-related and interactional code clusters within the analytic framework (see Table S3C, Methods and Supplementary Methods).

#### 3.4.6. Alterations in Relationships with Others

Participants described enduring changes in how they related to others beyond the abusive relationship, marked by withdrawal, mistrust, and shifts in expectations from interpersonal connections. These alterations extended into friendships, family relationships, and broader social interactions, often persisting even after exiting abusive environments.

##### Social Withdrawal and Isolating Oneself

Many participants described a gradual withdrawal from social relationships over time. While initial isolation was often externally imposed within abusive environments, it later became self-sustained, shaped by fatigue, shame, and difficulty re-engaging with others.

Ray’s current life was marked by significant social withdrawal, which she associated with both emotional exhaustion and physical fatigue. Renu described difficulty forming friendships and a preference for remaining within familiar familial circles. Among older participants, such as Maya, Priya, and Rhea, withdrawal was often attributed to ongoing responsibilities within the household and limited time; however, their accounts also reflected a persistent sense of loneliness and lack of meaningful emotional connection.

Across participants, social withdrawal appeared both as a continuation of earlier enforced isolation and as an adaptive response to prolonged distress, resulting in reduced engagement with social life.

##### Mistrust and Difficulty in Forming New Relationships

Participants consistently described difficulty trusting others and forming new relationships. These challenges were often linked to prior experiences of betrayal, neglect, or unmet expectations within close relationships.

Ray described a generalized mistrust of others, reinforced by repeated experiences of disappointment in friendships. Renu similarly reported that forming new connections was difficult, shaped by low confidence and hesitation in social situations. Among older participants, early marital experiences involving isolation from prior social networks further limited opportunities to build or maintain trust-based relationships over time.

These patterns suggest that mistrust became a generalized relational stance, extending beyond specific relationships and influencing broader social engagement.

##### Altered Expectations from Relationships

Participants described a shift in what they expected from relationships, often characterized by reduced emotional expectations and a move toward functional or pragmatic engagement.

Meera, for instance, described reaching a point where she no longer expected emotional fulfillment from her marriage, instead maintaining the relationship on the basis of coexistence and practical interdependence. Her account reflected a broader pattern in which expectations were recalibrated to minimize disappointment and maintain stability.

More broadly, participants described approaching relationships with increased caution, emphasizing the need for vigilance, self-protection, and strategic engagement. These shifts indicate a reconfiguration of relational expectations shaped by prior experiences of harm.

##### Revictimization

Some participants described patterns of entering multiple abusive relationships over time, often recognizing these patterns only in retrospect. Ray and Damini, in particular, described repeated involvement in relationships that shared similar dynamics of control, exploitation, and emotional harm.

Both participants linked these patterns to earlier experiences, including unmet emotional needs, isolation, and difficulties in recognizing or responding to warning signs in relationships. These trajectories suggest that prior relational experiences shaped subsequent relationship choices, increasing vulnerability to further harm.

Across participants, these patterns reflect how earlier experiences of trauma and relational control continued to influence interpersonal dynamics, extending the impact of abuse beyond a single relationship and into broader relational histories (see Table S3C, Methods and Supplementary Methods).

#### 3.4.7. Alterations in Beliefs and Meaning-Making Systems

Participants described shifts in their beliefs, values, and sense of meaning over time, often reflecting a loss of coherence in how they understood their lives, relationships, and future. These alterations were not abrupt but developed gradually, shaped by cumulative experiences of relational trauma and its psychological consequences.

Several participants described a growing sense of hopelessness, where distress was experienced as continuous rather than episodic. Ray, in particular, articulated a perception of life as persistently difficult, with diminished expectation of change or improvement. This was accompanied by a reduced ability to imagine a future that was meaningfully different from the present.

Participants also described disruptions in previously held beliefs about themselves and their values. Ray reflected on a loss of clarity about her own identity and principles, indicating a broader destabilization of meaning-making frameworks. Similarly, Renu described a shift in how she understood her own life trajectory, noting a stark contrast between her expectations prior to marriage and her subsequent experiences.

For some participants, these changes were linked to a sense of disorientation in relation to their own goals and purpose. Damini described a period of significant psychological distress prior to seeking professional support, during which she experienced difficulty organizing her thoughts, emotions, and sense of direction. Among older participants, such as Maya and Priya, these alterations were less explicitly articulated but reflected in narratives of enduring dissatisfaction, unrealized aspirations, and a narrowing of perceived possibilities.

Across participants, these accounts suggest that prolonged exposure to relational trauma reshaped not only emotional and relational functioning but also the broader systems through which individuals interpreted their experiences and located meaning in their lives. These patterns are reflected in meaning-making–related code clusters within the analytic framework (see Table S3C).

The patterns described in Theme 3.4 map onto the SPECTRA domain of Psychological and Somatic Adaptations, reflecting how prolonged exposure to relational trauma gives rise to enduring, trauma-focused adaptations across affective, cognitive, bodily, and relational domains. These alterations are not transient responses but become stabilized over time, shaping how participants experience themselves and the world. Notably, many of these adaptations persist even after exiting abusive environments, indicating that the effects of relational captivity extend beyond its immediate conditions. Together, these findings underscore how trauma becomes internalized and continues to organize functioning in the absence of ongoing external control.

### 3.5. Response, Negotiation, Resilience and Rediscovery

Participants’ narratives did not reflect passive endurance alone but revealed a range of responses over time, shaped by shifting constraints, relational dynamics, and sociocultural expectations. These responses were neither linear nor uniformly progressive; instead, they reflected ongoing negotiation within conditions that were often difficult to exit. Across accounts, participants described phases of breakdown, adaptation, resistance, and, in some cases, gradual efforts toward reclaiming aspects of their lives. These processes were deeply embedded in familial, social, and cultural contexts, where decisions were rarely individual and often carried significant relational and social consequences. Illustrative excerpts reflecting these trajectories of response, negotiation, and adaptive repair are presented in Supplementary File S10, Table S15.

#### 3.5.1. Initial Response: Subjugation and Breakdown

Across participants, the initial response to abuse was characterized by subjugation, confusion, and gradual psychological erosion rather than immediate resistance. Many described entering marital or intimate relationships with limited prior exposure to relational autonomy, which shaped their early interpretations of control, restriction, and conflict. As a result, early instances of abuse were often normalized, minimized, or interpreted as part of adjustment within marriage.

Participants described a period of disorientation in which expectations of care, stability, or companionship were disrupted, but not immediately replaced with clear recognition of abuse. Instead, there was a gradual accommodation to shifting relational norms, where compliance, silence, and endurance became primary modes of response. For several participants, especially those socialized into norms of adjustment and obedience, resistance was not initially perceived as viable, particularly when dissent led to escalation (Table 13).

**Table 13:** Silence and non-confrontation as enforced responses within conditions of subjugation (See Supplementary File S10, Table S16).

Participant	Excerpt
Rhea	*“Keep quiet and never protest… If you protest, there’s no way to calm it down. The only way was to stay silent. I couldn’t fight back… I still keep quiet.”*
Maya	*“Even if I’m angry, I used to keep quiet so that I don’t say anything harsh…never reply back”.*
Priya	*“I used to stay quiet to not trigger any more of these problems. Kept quiet, went to my room, Tried to ignore all of it- but I used to suffer a lot within myself.”*
Meera	*“I kept quiet. I didn’t know what to reply. Just avoid”*

Over time, this accommodation was accompanied by psychological strain and breakdown. Participants described increasing emotional distress, confusion, and a sense of losing control over their circumstances. Renu, for instance, recalled an early phase of her marriage marked by escalating volatility, where minor incidents led to disproportionate reactions, leaving her uncertain about how to respond. Priya similarly described a gradual intensification of emotional and relational strain, where repeated humiliation and exclusion were endured with limited protest due to fear of worsening consequences.

Among older participants, such as Maya and Rhea, early marital experiences were often framed retrospectively as periods of silent endurance, where restrictions, coercion, and emotional neglect were accepted as normative within the marital household. These responses were shaped not only by immediate relational dynamics but also by prior socialization that emphasized compliance and preservation of family stability over individual well-being.

Across participants, this phase reflects not a lack of awareness, but a constrained field of possible responses, where subjugation and psychological breakdown emerged as initial adaptations within environments that limited both recognition and resistance.

#### 3.5.2. Negotiating Difficulty, Resistance and Reclaiming Lives

Despite sustained constraints, participants gradually developed ways of negotiating their circumstances, demonstrating that their responses were neither static nor passive. These negotiations were shaped by practical realities, social pressures, and shifting personal thresholds, and often took the form of partial resistance, strategic accommodation, and incremental attempts to regain control. Returning to work emerged as one of the earliest and most significant strategies, offering not only financial independence but also mobility, identity, and psychological grounding within otherwise restrictive environments. For many participants, work functioned as a sanctuary from marital distress, enabling them to step outside the household and sustain a sense of self. At the same time, participants also engaged in personal efforts to regain control over their lives, including pursuing higher education, managing health conditions, and seeking psychological support. Returning to work emerged as a critical strategy, offering both financial independence and a sense of identity, even when it required sustained resistance within the marital household (see Table 14).

These efforts were not uniform but reflected varying degrees of awareness, access, and possibility. Participants’ trajectories diverged significantly across contexts: Ray remained in a relationship she experienced as entrapping while attempting to leave; Damini actively addressed her mental health and entered a more supportive marriage; Renu exited her marriage and began rebuilding her life despite ongoing challenges; Priya separated early but pursued legal divorce much later, mentioning it took her years to get over her failed marriage; Maya remained separated without formal dissolution and continued experiencing emotional distress; and Meera and Rhea continued within their marriages while negotiating distance and adjustment. These varied pathways reflect how responses to abuse were shaped by intersecting sociocultural constraints and opportunities rather than following a single trajectory.

**Table 14:** Reclaiming work and identity as an act of resistance within constrained marital environments (See Supplementary File S10, Table S17).

Participant	Excerpt
Rhea	*“After 16 years, I realized I could come out of it… every woman should have an identity.”* *“One day I decided enough is enough… the next day I got an interview, and that week I got the job.”* *“No one accepted it at first—not even my husband—but I did not listen. Later, everyone accepted… now even the daughters-in-law work.”*

##### The Inescapability of the Marriage

For many participants, marriage was experienced as structurally and culturally inescapable, shaped by deeply embedded social norms, economic dependencies, and moral expectations. Divorce was not merely difficult but often unimaginable, particularly within middle-class contexts where marriage is constructed as sacred, permanent, and central to family identity. Participants described remaining in abusive relationships despite recognizing harm, constrained by fear of social judgment and the stigma associated with separation (see Table 15). Economic and familial dependencies further reinforced this condition. In some cases, marital families provided financial support to natal households, creating enduring feelings of obligation that limited the possibility of exit. The absence of male support within natal families, particularly the lack of a father or brother, intensified vulnerability and reduced perceived options. These constraints were embedded within cultural practices that symbolically transfer a woman’s belonging from her natal to marital home, reinforcing the expectation that marriage is not easily reversible. Across participants, inescapability reflects a convergence of social, economic, and cultural forces that restrict the field of possible action rather than a lack of awareness or will.

**Table 15:** Social stigma and moral expectations as constraints on exiting marriage (See Supplementary File S10, Table S18).

Participant	Excerpt
Rhea	*“Thinking of divorce… we couldn’t even dream of it. It was a social taboo.”* *“In our time, only girls with strong financial or family backing could think of leaving. I had no brother, my father had passed away, and I wasn’t earning—so all responsibility was on me. That also shaped how I stayed.”*
Priya	*“I knew this [her marriage] wouldn’t work… but I was afraid of society.”*
Meera	*“That is not a solution to walk out of the marriage.”*

##### Separation as a Compromise

When separation did occur, it was often pursued as a negotiated alternative to divorce rather than a definitive break. Participants described forms of partial disengagement that allowed distance from the abusive environment while maintaining the formal structure of marriage.

In several cases, returning to the natal home functioned as a critical mechanism enabling separation. Maya and Priya were able to leave their marital homes and reside with their families, supported by relatively stronger natal networks. However, this return was not understood as a permanent exit from marriage but as a provisional arrangement within culturally accepted boundaries.

Among older participants, separation was less common, but forms of partial disengagement were evident. Meera described gradually withdrawing emotionally from her marriage, recalibrating expectations and maintaining the relationship in a more functional than affective capacity. This form of internal separation allowed for a degree of stability without formally exiting the relationship.

Across cases, separation—whether physical or emotional—was shaped by constraints and consequences, and rarely represented a clean break. Instead, it marked a transition into new forms of negotiation, often accompanied by continued entanglement with prior relationships.

##### The Communal Spectacle and the Construction of an Ideal Victim

Participants’ experiences were embedded within social environments in which their actions were observed, interpreted, and judged, producing a form of communal spectacle. Abuse and responses to it were not confined to the private sphere but became visible and subject to public evaluation, often in ways that intensified participants’ distress. Renu’s experience of being publicly shamed while attempting to leave her home illustrates how humiliation operated as both a relational and social mechanism, reinforcing control through reputational damage. Similarly, participants described fear of social judgment, lack of support, and community apathy as factors that shaped their decisions and constrained their responses. Participants’ experiences of abuse and their responses to it were not confined to the private sphere but unfolded within a broader social field in which actions were observed, interpreted, and judged. This created a form of “communal spectacle,” where participants were required to navigate not only the abuse itself but also expectations about how a “good” woman or daughter-in-law should behave under such conditions.

Meera’s experience illustrates how reputational dynamics within extended family and community networks shaped both her constraints and her choices. Despite enduring prolonged hostility and psychological strain within her marital home, her actions were continuously subject to social interpretation. Her mother-in-law’s ongoing antagonism extended beyond the household, affecting how Meera was perceived socially, contributing to her sense of isolation and distress.

When Meera eventually arranged for her mother-in-law to be moved to an eldercare facility due to concerns about safety and lack of support, this decision was not evaluated in terms of necessity or care, but through a moral lens that positioned her as failing in her duties as a daughter-in-law. Her husband’s initial resistance, and his continued dissatisfaction even after the decision was implemented, reflected this framing. The expectation that she should continue to provide intensive care—despite her own well-being, professional responsibilities, and lack of support—remained intact.

At the same time, Meera’s decision required mobilizing extended family involvement, as she lacked allies within her immediate household. Even after institutional care was arranged, her responsibilities did not diminish; she continued to manage caregiving tasks, demonstrating that compliance with expectations did not necessarily restore social legitimacy (see Table 16).

**Table 16:** Community-level stigma and reputational harm as extensions of relational conflict, Moral scrutiny and social judgment in enforcing normative expectations of caregiving and sacrifice (See Supplementary File S10, Table S19).

Participant	Excerpt
Meera	*“Socially I was very much affected… that brought my mental trauma.”* *“Even now, he is still dissatisfied that I kept her in an old age home.”*

Within this framework, legitimacy as a victim was contingent not only on suffering but also on conformity to expected norms of endurance and conduct, making resistance socially risky.

##### Children as a Catalyst

Children played a decisive yet complex role in shaping participants’ responses. While fear of stigma and social consequences often delayed action, concern for children’s well-being frequently acted as a catalyst for change. Participants described becoming more willing to resist or leave abusive environments when they perceived direct harm to their children. Maya’s separation, initiated at her son’s urging to protect his sibling from ongoing abuse, illustrates how children could prompt decisive action where self-protection alone did not (see Table 17).

Priya similarly chose to leave soon after her daughter’s birth, prioritizing her child’s future over her own continued endurance within the marriage. Meera’s consideration of separation was closely tied to concerns about her daughter’s safety in the context of her mother-in-law’s erratic behavior, demonstrating how maternal responsibility reshaped thresholds for action. Across participants, decisions were deeply relational, with children functioning both as constraints and as catalysts, complicating the process of negotiation and making responses contingent on broader familial considerations.

#### 3.5.3. Rediscovery and Adaptive Repair

Participants’ accounts reflected gradual and uneven processes of rediscovery and attempts to reorganize their lives following prolonged relational trauma. These processes did not represent a return to a prior state but involved reconstructing a sense of self, stability, and direction under altered conditions. For some participants, natal families functioned as critical sites of safety and intervention. In Renu’s case, disclosure of her situation prompted immediate action by her family, who facilitated her exit from the marital home and recognized the extent of her psychological distress. This support enabled her to begin rebuilding her life while also confronting the longer-term consequences of trauma. Her engagement with therapy was directed not only toward managing distress but also toward developing the confidence to navigate legal systems and articulate her experiences. These processes of rediscovery often involved a gradual reclaiming of voice and self-assertion after prolonged periods of silence and constraint (See Table 18).

Participants also described efforts to actively reshape relational dynamics. Damini’s ability to resist verbal abuse contributed to a shift in how she was treated within her marital home, while her husband’s efforts to manage his own difficulties enabled partial stabilization of the relationship. Among older participants, rediscovery often occurred later in life and was shaped by necessity rather than a linear trajectory of recovery. Rhea’s return to work represented a significant assertion of identity and independence, while Meera’s decision to institutionalize care for her mother-in-law marked a decisive reassertion of control within a prolonged and constrained context. In cases where participants remained within their marriages, adaptive repair took the form of recalibration rather than restoration. Relationships were renegotiated toward reduced conflict and pragmatic coexistence, without full emotional resolution or equality. Participants also described developing evolving practices of self-care that enabled them to manage ongoing distress and reclaim personal space within constrained lives. For some, this took the form of reconnecting with social networks and creating routines outside the household, including regular meetings with friends and, in Meera’s case, annual trips that provided respite from caregiving and domestic responsibilities. Others described quieter, individualized practices such as reading, gardening, meditation, and spiritual engagement, which offered moments of calm and continuity. Work itself often functioned as a stabilizing structure, while small, self-directed activities—whether time with grandchildren, volunteering, or engagement with nature—became important ways of restoring a sense of control, pleasure, and selfhood in everyday life. Across cases, rediscovery and adaptive repair were shaped by available resources, relational contexts, and sociocultural constraints, reflecting not resolution but the capacity to reorganize life in the aftermath of sustained disruption.

Theme 3.5 maps onto the SPECTRA domain of Negotiation and Adaptive Repair, capturing how participants actively engaged with, responded to, and gradually reworked their circumstances within and beyond conditions of relational constraint. These processes were not linear or uniformly agentic, but involved ongoing negotiation shaped by structural, relational, and sociocultural limits. Responses ranged from subjugation and endurance to partial resistance, separation, and attempts at rebuilding aspects of the self and everyday life. Together, these patterns reflect adaptive efforts to manage and reorganize life in the aftermath of prolonged relational trauma, often coexisting with its enduring psychological and social effects.

## 4. Discussion

The present study advances a relational and culturally grounded understanding of domestic violence as a continuous, developmentally embedded, and socially organized process rather than a series of discrete incidents. Across the illustrative cases, violence emerged as a cumulative relational condition shaped by early gendered socialization, kinship structures, and enduring psychological and bodily adaptations. Taken together, the findings support SPECTRA as a theory-building framework that bridges developmental, relational, and sociocultural dimensions of trauma within patriarchal contexts. The broader theoretical provisions, conceptual rationale, and intended research and intervention utility of the SPECTRA framework are elaborated in, which presents the model as a feminist, culturally grounded conceptual scaffold for understanding domestic violence as continuous trauma embedded in relational architectures.

### 4.1. Domestic Violence as Continuous Relational Trauma

The findings suggest that domestic violence is best understood as a chronic relational system unfolding across the life course. Harm was experienced not as isolated acts but as interconnected processes spanning natal and marital contexts, progressively reshaping women’s sense of safety, selfhood, relational trust, and agency. Violence was often sustained through extended family structures, in-law authority, and normative expectations of adjustment, duty, and respectability, embedding coercion within socially legitimate roles.

The consequences of prolonged exposure extended beyond disorder-specific symptom language. Participants described enduring psychological and bodily changes, constriction of relational worlds, and complex negotiations of endurance, withdrawal, and exit. These are more appropriately understood as adaptive responses to sustained relational threat than as decontextualized pathology.

### 4.2. From Episodic Framing to Relational Captivity

A central contribution of the study is its challenge to episodic framings of domestic violence within psychology and public health. By tracing women’s experiences across developmental and relational contexts, the findings foreground violence as cumulative and socially sustained rather than event-bound.

Within this reconceptualization, relational captivity emerges as a key theoretical contribution. This extends existing work on coercive control by showing how entrapment in the present context is co-produced through kinship hierarchies, stigma surrounding separation, and socially legitimate expectations of adjustment. In this sense, captivity is not limited to partner-level control but is embedded within broader relational architectures of patriarchal authority. Entrapment was maintained not only through dyadic coercion but also through layered mechanisms of surveillance, mobility restriction, dependency, social isolation, stigma surrounding separation, and kinship-based authority. These forces progressively reorganized women’s anticipatory worlds, making compliance and strategic silence adaptive modes of survival.

This formulation recentres patriarchal power as relational architecture rather than merely interpersonal behavior aligned with extant literature (Vindhya, 2007; Gangoli & Rew, 2011; Heise, 2011; Vindhya, 2020). Violence is thus understood as scaffolded by gendered social scripts that precede and outlast specific abusive episodes.

### 4.3. Trauma as Adaptation Rather than Pathology

The psychological consequences of domestic violence are more usefully understood as adaptations to prolonged relational threat than as discrete symptom clusters. Participants’ narratives reflected enduring shifts in affect, bodily experience, self-worth, trust, and meaning-making that were inseparable from context.

These alterations are best interpreted as survival-oriented recalibrations to chronic uncertainty, coercion, and emotional unsafety. These patterns converge with established complex trauma literature on altered self-perception, relational disturbance, and chronic affective dysregulation (Herman, 1992a, 1992b; Herman, 2015; Luxenberg et al., 2001; van der Kolk et al., 2005; van der Kolk, 2014; Cloitre, 2020), while the present findings extend such models by locating these adaptations within culturally sustained relational threat rather than solely within individual symptom frameworks. Somatic distress, hypervigilance, internalized guilt, and identity disruption reflect how trauma becomes stabilized within both body and self-structure. This perspective retains the analytic value of trauma theory while resisting the tendency to depoliticize distress by locating it solely within the individual as critics have pointed out (Davar, 2002; Burstow, 2003).

### 4.4. Agency and Adaptive Repair Within Constraint

Participants’ responses to violence cannot be reduced to binaries of victimhood and resistance. Instead, the findings reveal ongoing negotiation within structural constraint, including strategic silence, selective compliance, boundary-setting, help-seeking, and, in some cases, exit.

A feminist relational reading requires that agency be understood contextually. Remaining, withdrawing, separating, or preserving family stability may all function as meaningful forms of self-preservation within conditions shaped by stigma, dependency, and kinship obligations.

Repair likewise emerged as ongoing rather than complete, involving the gradual reconstruction of autonomy, self-worth, social support, and relational boundaries while often coexisting with enduring distress.

### 4.5. Sociocultural and Contextual Contributions

A major contribution of the study lies in situating domestic violence within gendered relational architectures shaped by kinship, marriage, duty, and social legitimacy. Violence frequently unfolded beyond the partner dyad through in-law hierarchies, moral expectations, and culturally sanctioned authority structures (Gangoli & Rew, 2011; Heise, 2011).

Developmental socialization within natal families also appeared to shape later thresholds of recognition, endurance, and negotiation. This continuity supports a life-course perspective in which violence is embedded within broader patriarchal scripts rather than confined to marital episodes alone. An additional axial pattern emerging across the narratives concerns age-linked differences in the recognition, articulation, and negotiation of violence. Within the present sample, younger women appeared more likely to identify coercive behaviors as abuse and to articulate boundaries or resistance, whereas older women’s narratives more often reflected longer periods of normalization, endurance, and delayed reinterpretation. Older participants also expressed stronger beliefs in the sanctity of marriage, internalized stigma surrounding divorce, and continued guilt and emotional distress following separation or divorce. While this observation remains exploratory and specific to the present cases, it may reflect generational shifts in sociocultural vocabularies surrounding domestic violence, mental health, and women’s autonomy.

Several themes emerging across the cases—including divorce as taboo within middle-class value systems, conditional support from natal homes and children, normalization of abuse, restrictions on autonomy, patriarchal revictimization, and the idealized bahu identity—underscore the inescapable relationality of women’s lives within patriarchal family structures in India. Taken together, these patterns position patriarchy not merely as background context but as an overarching structural perpetrator that organizes domestic violence through culturally sanctioned scripts of duty, endurance, and role compliance. These observations are consistent with prior work on domestic violence, marital stigma, and the social regulation of women’s identities in India (Vindhya, 2007, 2020; Ahmed-Ghosh, 2004; Ghosh, 2011; Agnes & D’Mello, 2015; Dommaraju, 2016; Berkl & Jain, 2018; Capelos & Basu, 2021; Abdul Azeez et al., 2024; Bhandari & Hughes, 2017).

Notably, some of these trends appeared more prominent among older respondents, whose narratives more often reflected prolonged normalization of abuse and stronger internalization of caregiving and marital obligations. While younger participants appeared to describe somewhat greater educational and aspirational autonomy within natal homes, this did not necessarily translate into reduced structural burden. Existing evidence suggests that women’s entry into professional spaces has not been accompanied by a commensurate reduction in domestic and caregiving labor, and women’s workforce attrition continues to be shaped by the prioritization of familial duty over professional identity (Vindhya, 2007; Arabandi, 2016; Abraham et al., 2021; Dalal et al., 2025). Similarly, the theme of work–life imbalance closely mirrors the documented gender inequity in the distribution of unpaid domestic labor in India, where women continue to shoulder a disproportionately larger share of household responsibilities irrespective of employment status (Addati et al., 2018). The qualitative accounts in the present study reflect this structural pattern, with domestic and caregiving duties remaining overwhelmingly feminized and normalized as part of women’s expected familial roles. Together, these findings reinforce the importance of understanding age and life stage as axial dimensions through which domestic violence, duty, and constrained agency are differentially negotiated.

This sociocultural grounding gives SPECTRA its analytic value as a culturally situated, data-led framework rather than a direct extension of existing trauma models. Relational architecture may therefore be understood as the foundational analytic layer of the framework, capturing how violence is scaffolded through duty, respectability, family honor, and socially regulated thresholds of protest and exit.

### 4.6. Implications

The present findings support movement beyond event-based and disorder-centered models toward frameworks capable of capturing developmental continuity, relational embeddedness, and sociocultural mediation. For trauma-informed practice, this suggests the need for approaches that address not only symptom reduction but also identity disruption, relational distrust, bodily distress, and the ongoing structural constraints within which survivors continue to negotiate safety and agency. Existing trauma-informed literature has already established the value of stage-wise, modular, and severity-sensitive approaches, with trauma-focused interventions demonstrating stronger outcomes than non-trauma-informed care, particularly in the context of prolonged interpersonal trauma. However, emerging evidence also indicates that such interventions require careful cultural adaptation when applied beyond the Western settings in which they were largely developed. In this regard, the present framework does not seek to replace existing trauma-informed interventions (Herman, 2015; Karatzias & Cloitre, 2019; Coventry et al., 2020; Jewkes et al., 2021, Heim et al., 2022, Karatzias et al., 2024). Rather, SPECTRA may serve as a culturally grounded conceptual scaffold to guide the adaptation of established stage-wise and modular trauma approaches by incorporating domains such as identity reconstruction, relational healing, bodily and psychological adaptation, and negotiated autonomy within enduring structural constraints. More broadly, the framework may support the future development of culturally grounded research and intervention models that better capture the lived continuity of domestic violence and its long-term psychological and relational sequelae in community as well as clinical settings.

### 4.7. Limitations and Future Directions

The study is based on a small set of illustrative qualitative cases drawn from educated, urban women in India. Its purpose is theory building rather than representativeness. Accordingly, SPECTRA should be understood as an emergent and contextually situated framework rather than a finalized model.

Future work should examine how the framework holds across more diverse social locations and in longitudinal designs capable of tracing violence, adaptation, and negotiated agency across time. Future empirical work should also examine whether the framework requires modification across intersecting axes such as caste, class, and ethnoreligious regulation, rather than presuming transferability in advance.

## 5. Conclusions

Taken together, the findings support a shift from event-based models of domestic violence toward an understanding of violence as continuous, relationally embedded, and culturally mediated. SPECTRA offers an analytically grounded framework for organizing developmental embedding, relational captivity, adaptation, and negotiated repair within patriarchal contexts, while remaining open to further empirical refinement.

## Figures and Tables

**Table 11:** Psychological Alterations in Complex Trauma Literature. This table summarizes key domains of psychological alterations described in complex trauma literature, including somatization, affect dysregulation, and disruptions in attention and consciousness. Adapted from foundational formulations (e.g., Herman, 1992b; Luxenberg et al., 2001), it provides a conceptual reference for understanding how prolonged trauma has been characterized in prior work. The table is intended to situate, rather than determine, the interpretation of patterns observed in the present study.

Domain of Psychological Alterations	Specific Psychological Presentations	Examples	Conceptual Description (Based on Complex Trauma Literature)
Somatization	Chronic pain, fatigue, headaches; gastrointestinal, cardiopulmonary, and sexual symptoms; appetite and sleep disturbances	Persistent unexplained medical complaints (e.g., chronic pain, fatigue, sleep disturbances, gastrointestinal distress); worsening of existing conditions	Complex trauma literature describes somatic symptoms as embodied expressions of prolonged stress, where distress is experienced and communicated through the body rather than through explicit psychological articulation.
Affect and Impulses	Persistent dysphoria; emotional lability; explosive or inhibited affect; difficulty with self-soothing; impulsivity and risk-taking	Chronic sadness, rage, emotional numbing, suicidal ideation, affective instability, impulsive or self-destructive behaviors	Survivors of prolonged trauma often exhibit chronic affect dysregulation, characterized by oscillation between emotional numbing and overwhelming affect. Emotional responses may be amplified, suppressed, or difficult to regulate, reflecting enduring alterations in emotional processing.
Attention and Consciousness	Dissociation; amnesia; altered states of consciousness	Memory gaps, depersonalization, detachment, fragmentation of experience, difficulty concentrating	Complex trauma literature identifies dissociation and altered consciousness as adaptive responses to overwhelming stress. These include disruptions in memory, attention, and continuity of experience, enabling psychological distancing from distressing events.

**Table 12:** Psychological and Relational Alterations in Complex Trauma Literature. This table outlines additional domains of alteration identified in complex trauma literature, including disruptions in self-perception, relationships, perceptions of perpetrators, and meaning-making systems. Adapted from prior theoretical and clinical formulations (e.g., Herman, 1992b; Luxenberg et al., 2001), it highlights how prolonged trauma has been conceptualized as affecting identity, relational schemas, and interpretive frameworks. The table serves as a comparative backdrop for situating the study’s findings within existing literature.

Domain of Psychological Alterations	Specific Psychological Presentations	Examples	Conceptual Description (Based on Complex Trauma Literature)
Self-Perception	Shame, guilt, self-blame; chronic helplessness; sense of permanent damage	Feeling worthless, guilty, damaged, or fundamentally flawed	Complex trauma literature describes persistent shame, guilt, and self-blame as central features of prolonged abuse. These experiences contribute to a disrupted and negatively valenced sense of self, often accompanied by a perceived loss of agency and control.
Perception of Perpetrator	Preoccupation with the abuser; attribution of power; ambivalent attachment (fear, loyalty, gratitude)	Viewing perpetrator as powerful; minimization of abuse; emotional attachment despite harm	Survivors may develop ambivalent and dependency-based attachments to perpetrators, often described as trauma bonds. These relationships are characterized by fear, loyalty, and rationalization of abuse within conditions of prolonged control and isolation.
Relationships with Others	Social withdrawal; mistrust; difficulty forming relationships; revictimization	Avoidance of relationships; inability to trust; repeated involvement in harmful relationships	Prolonged trauma is associated with disruptions in relational capacity, including difficulties with trust, intimacy, and mutuality. Survivors may withdraw socially or re-enter harmful relational patterns, reflecting enduring alterations in relational expectations and schemas.
Meaning- Making Systems	Hopelessness; loss of sustaining beliefs; existential crisis	Fatalism, loss of purpose, diminished belief in change or future possibilities	Complex trauma literature highlights disruptions in meaning-making systems, including loss of belief in safety, purpose, or future orientation. Survivors may adopt fatalistic or despair-oriented frameworks in interpreting their experiences.

**Table 17:** Children as emotional anchors and catalysts for change, enabling action and social reconnection (See Supplementary File S10, Table S20).

Participant	Excerpt
Maya	*“(About abuse) my elder son said (to her husband), ‘Let us stay separate and you stay separate, and let us stay in peace.”*
Meera	*“I was very traumatized… my daughter told me, ‘Move on. Don’t think about the family. Find your friends.’”* *“Through Facebook I found my old friends again… my social life opened up.”* *“Only time I considered leaving marriage was if I could not place my mother-in-law in an eldercare, for my daughters’ sake.”*

**Table 18:** Reclaiming voice and agency as part of adaptive repair (See Supplementary File S10, Table S21).

Participant	Excerpt
Renu	*“I needed the strength to face the system… to raise my voice and put it out there.”*
Damini	*“Now I speak back… they don’t talk to me the same way anymore.”*

## Data Availability

Data for this study is not publicly available because of its sensitive nature, and out of privacy and ethical concerns related to study participants. Deidentified data may be shared by the authors upon reasonable request, at the discretion of the corresponding author, only for the purpose of academic research. In all the supplementary files and text containing case information, participants’ and their partners’ names have been changed for confidentiality and their narratives deidentified as far as possible, like specific places of residence or employment.

## Supplementary Materials

The following supporting information can be downloaded at: https://www.mdpi.com/article/10.3390/bs16050814/s1, File S1: Case Study: Ray; File S2: Case Study: Damini; File S3: Case Study: Renu; File S4: Case Study: Priya; File S5: Case Study: Meera; File S6: Case Study: Maya; File S7: Case Study: Rhea; File S8: SPECTRA-Supplement; File S9: Methods-Supplement; File S10: Results—supplement.

## Abbreviations

The following abbreviations are used in this manuscript:

cPTSD	Complex Post-Traumatic Stress Disorder
DESNOS	Disorders of Extreme Stress Otherwise Not Specified
DSM	Diagnostic and Statistical Manual of Mental Disorders
DTD	Developmental Trauma Disorder
DV	Domestic Violence
GBV	Gender-Based Violence
IPV	Intimate Partner Violence
LFPR	Labor Force Participation Ratio
LICs	Low-Income Countries
LMICs	Low- and Middle-Income Countries
NFHS	National Family Health Survey
PTSD	Posttraumatic Stress Disorder
SPECTRA	Socially and Psychologically Embedded Continuous Trauma in Relational Architecture
VAW	Violence Against Women
WHO	World Health Organization
