# Supplementary material for: SPECTRA: A Conceptual Framework to Bridge Praxis and Remap Relational Violence in India Using a Complex Trauma Lens
Source: Behav Sci (Basel). 2026 May 19;16(5):814. doi: 10.3390/bs16050814 (PMC13203196; doi:10.3390/bs16050814)
Supplement: Supplementary file 1 [file behavsci-16-00814-s001.zip › behavsci-4012693-supplementary.pdf]

SPECTRA: A Conceptual Framework to Bridge Praxis and Remap Relational Violence in India Using a Complex
Trauma Lens

Maitrayee Sen, Snigdhaa Rajvanshi †, Stuti Khandelwal † and Simantini Ghosh *

Department of Psychology, Ashoka University, Sonipat 131029, India;
maitrayee.sen_phd19@ashoka.edu.in (M.S.); snigdhaa.rajvanshi@alumni.ashoka.edu.in (S.R.);
stuti.khandelwal@alumni.ashoka.edu.in (S.K.)
* Correspondence: simi@ashoka.edu.in
† These authors contributed equally to this work.

Supplement: Organization

File S1-S7: Case Studies

- File S1: Case Study: Ray
- File S2: Case Study: Damini
- File S3: Case Study: Renu
- File S4: Case Study: Priya
- File S5: Case Study: Meera
- File S6: Case Study: May
- File S7: Case Study: Rhea

File S8: SPECTRA supplement

- SPECTRA domains
- Supplementary Table S1 : Table S1: SPECTRA domains and explanations (Extended)
- Supplementary Table S2: Conceptual Comparison of Clinical Trauma Frameworks and SPECTRA

File S9: Methods supplement

- Supplementary methods
- Supplementary Tables S3A-D

File S10: Results Supplement

- Full Thematic Map
- Supplementary Tables S4-S22

File S1: Case Study: Ray

Ray (29)

Demographics and Family Background

Ray is 29 years old and she resides in Kolkata, which is her hometown. She holds an M.Phil and is currently pursuing her PhD. Ray's immediate family includes her parents and a younger brother. Her parents were born in a town outside the main city, and her grandparents were originally from Bangladesh. Her father retired from a major consulting firm in 2019, and her mother is a housewife. The family's income currently relies on savings, rent from flats (approximately ₹12,000 per month), and Ray's UGC scholarship (₹31,000 per month). Ray is not completely financially independent.

Ray's childhood was characterized by fear, as she was terrified of her father. Her father had extreme, uncontrolled anger issues. He was the primary decision-maker and controller of finances in the household. Ray did not have a safe relationship with her parents, though she initially went to her mother for comfort until her brother was born. Family life was marked by constant, huge conflicts, often involving Ray's father's family being abusive toward her mother. Ray believes her younger brother, who is 6 years her junior, received preferential treatment, possibly due to his gender or being born later, as her father was less violent with him and her mother devoted more time and special care to him. Her mother was her only source of comfort, but when her brother was born, her mother and her brother were both sick for a long time, and spent a lot of time lying down together in the same room, where she had very little access. This caused her to feel lost, and she has occasionally felt jealous of her brother because he took so much of her mother's attention away from her. He got more gifts than her, although she was not sure if it was because they had more money later. Her relationship with her parents during childhood was "*quite terrifying*" due to her father's abusive behaviour, which culminated in her father beating her violently on multiple occasions. She found comfort in her mother until her brother's birth, after which that relationship also suffered. She felt her mother was spending most of her time with her brother and became emotionally distant from her mother as well. She never felt safe sharing things with her parents. The family experienced *"huge conflicts,"* often involving her father's entire family being abusive towards her mother, a dynamic Ray describes as "*typical how Indian wives are treated by their in-laws"*. Even though she does not know the details, she

has a hunch that it is all about hurtful things that her grandmother said or did to her mother. Ray's father frequently beat her. Her father maintained almost complete control over family decisions and finances, with the only significant decision her mother made being the insistence to move flats in 2019. Ray remembers her teenage years being very traumatic, because her mother was also probably depressed at this time, and her behaviour towards Ray changed a lot. She reports her current relationship with her mother as "*still very weird*".

Ray also suffered from terrible abdominal pain since age 10, but neither of her parents took it seriously. She later found out that she had endometriosis, and reports to have suffered severely because of medical gaslighting and gynecologists' tendencies to minimize women's pain related to menstrual discomforts. The doctors she independently saw classified her pains as regular menstrual cramps despite their debilitating severity and told her these will go away after marriage and childbirth. She spent years trying to research the reasons for her own suffering for years before making a breakthrough. Even then, she could not find a sympathetic doctor who would even order the diagnostic tests that would confirm endometriosis. It was only last year she found a sympathetic doctor and clinic through social media who confirmed the diagnosis, however, by then the disorder had spread to her entire abdomen and "damaged many organs". Despite a recent surgery, neither the doctor , nor Ray herself was convinced if the damage could be repaired fully. She lives with severe pain and limited mobility. Ray attributes her deeply troubling history of relationships partially to all the cumulative lack of emotional support from her family and from the terrible ignorance and indifference she has received from the medical fraternity when she has sought treatment for her ever-worsening pains.

1. **Career**

Ray is focused solely on her PhD studies, having completed her B.A., M.A., and M.Phil. She has limited professional experience, having worked only for a few months on a research project after her M.Phil. A traumatic experience occurred when she was not allowed to study science at her preferred school despite good grades, which she suspects was due to financial reasons or donations. She later joined JNU in 2011 to study English, hoping to become a fiction writer, not an academic. She was discouraged from pursuing these interests. As a researcher, her work schedule is described as erratic

and unpredictable, dependent on presentations, assignments, and fieldwork interviews. Due to her struggles with both physical and mental health, she states that she is currently "*just surviving*".

2. **History of Romantic relationships**

a. Past Relationships

Ray has been in three relationships in total.

1. **First Relationship (Ashish, Duration: 2.5 years, ending 2015):** Ray entered this relationship at age 20. It ended due to differing ideologies, as Ashish was "*proudly misogynistic*" and desired a traditional marriage. Ray felt she had some autonomy in this relationship, being "*allowed to speak*" and express disagreements, with conflicts generally revolving around their opposing views, although her partner would say "*horrible things*" during arguments. Ashish was possessive and grew suspicious of and objected to anyone leaving a comment of appreciation on any of her photos on social media .He commented on her dressing style, suggesting more traditional clothes, which she unconsciously adopted towards the end of the relationship, though she felt it wasn't outright forced. Ray later vaguely recalled instances of physical abuse in this relationship, and that Ashish hit her once or twice.Towards the end of that relationship she remembers him pushing her in the path of a car while they were fighting. The car was not moving fast so she was not injured. She had largely "*forgotten*" these physical incidents, focusing more on the emotional scars. Ray mentions she thought all these were signs of a bad relationship, but her relationships got progressively worse. She views this relationship, in retrospect, as "*not that bad*" compared to subsequent ones, as they at least had a two-way discussion.

2. **Second Relationship (Pavan) (Duration: 9-12 months; 6 months of happy relationship, followed by pure abuse):** The initial six months (starting late 2017) were highly positive, described as a "*dream*". After her first misogynistic relationship, she was cautious but "*miraculously found somebody like that online"* who seemed perfect – kind, supportive, and understanding of her anxieties and trauma, including physical health issues. During the first few months, Pavan even expressed intentions to move to Kolkata for a job, assuring her it wasn't

solely for her. Ray felt her mental health was at its best during this period, free from anger, sadness, or anxiety. She felt completely supported and her partner initially seemed compassionate, a feminist ally and understanding. However, around March 2018, as Ray stated, Pavan "*changed completely, taking a 180-degree U-turn*". He refused to move to Calcutta on his father's advice , suddenly took a very materialistic turn about lack of career opportunities in Kolkata, and started pressuring her to shift to Delhi instead. He demanded she move immediately, despite knowing her health issues and aversion to Delhi, and previous agreements not to live there. He started imposing impossible deadlines for moving on her. This marked the beginning of "*pure abuse*," characterized by unachievable demands, emotional manipulation, and blocking her on all platforms. Pavan would write messages, give dead-voiced ultimatums, and block her before she could reply, and then ghost her for extended periods of time, making it a "*one-sided relation*". She would continuously try to plead her case and pacify him. Ray felt she "*could not afford to lose him*" and did whatever Pavan said, even traveling to Delhi twice despite her fear and illness to keep him in her life. He constantly changed his conditions and accused her of lying and not loving him. She was going through her M.Phil submission and severe gynecological problems (endometriosis and adenomyosis), which made moving incredibly difficult. He also expected her to live separately and pay for everything in the more expensive city of Delhi. Ray still doesn't believe she has fully recovered from this relationship, describing it as a "*mess*" that led her into her current situation.

Ray suffers from debilitating pain due to endometriosis and adenomyosis, diagnosed only last year, after years of doctors and family dismissing her pain because she wasn't married. This discrimination and lack of treatment added to her trauma. Her adenomyosis and endometriosis has spread to her entire abdomen, and all organs are damaged by her current evaluations, finally explaining her debilitating pains. She suspects she has many autistic traits, which makes dealing with major life changes particularly difficult. After the breakup Ray felt betrayed from the abandonment, and found her loneliness further unbearable compounded by her medical trauma. Her isolation and need for support, especially regarding her health, influenced her entry into the current relationship.

b. Current relationship (Mohsin, Last 3 years)

Ray has been talking to her current partner, Mohsin, on and off for three years. She met Mohsin, a homeopathic doctor, online while she was desperately seeking distraction from the pain of her previous relationship, despite knowing his profile had clear "*red flags*," including his dogmatic stance on religious matters. More than anything, initially she had been hoping he would understand her suffering from health problems and how her case was mishandled by the hegemonic medical industry, because of his background in alternative medicine. Initially Mohsin was supportive and sweet, and she felt grateful for him. After the relationship began, she had to have surgery, and after the surgery, she had gained weight, and frustrated from all the side effects of the hormonal treatments shaved her head. At that time, he supported her entirely, for which she seems to be extremely grateful to him. That a religious Muslim man could *"love a fat girl with her head shaved"*, became evidence to her of her being more feminist than herself or her friends in college who were all biased against her, despite their progressive posturing. However, Mohsin increasingly grew unpredictable and she reports him as constantly sending her mixed signals. She also reports a very high degree of emotional violence every single day. Ray is unsure of her relationship status, noting that Mohsin gives her different answers daily, which she considers abusive.

This relationship is characterized by daily, continuous, and extreme mental abuse, which Ray finds worse than she could have imagined. Mohsin is described as exhibiting "*hot and cold*" behavior, switching rapidly between declarations of love and extreme insults and threats. Ray feels entirely isolated and desperate, which contributes to why she stays, hoping for the rare moments of his "*sweet side*".

Abuse History

a. Abuse in natal family

Ray experienced psychological and physical abuse from her father beginning in childhood. Her father used to hit Ray and her mother, demean them, and shout, but masked his behavior socially. Growing up was terrifying for Ray. She also faced systemic issues in her childhood, such as being prevented from pursuing her desired academic path.

b. Abuse in romantic and marital/cohabiting relationships

Abuse has been present in all three relationships:

- **First Partner(Ashish):** Included verbal abuse and possessive behavior. He physically pushed her in front of a slow-moving car during a fight near the end of the relationship, and Ray believes he hit her once or twice.
- **Second Partner(Pavan) :** Involved extreme emotional abuse, manipulation, emotional coercion, and controlling demands regarding her relocation and submission of her M.Phil thesis.
- **Current Partner (Mohsin):** The abuse is daily, extreme, and multi-faceted:
    - **Physical Violence:** Mohsin was physically violent once, pinning her against a wall with his hands around her neck, attempting to strangle her. Ray describes his anger as something capable of turning him to a "*monster*" and "*worse than*" her father, which she had not thought possible.
    - **Verbal/Emotional Abuse:** Mohsin constantly uses gaslighting, making everything a conflict, insulting her intelligence, mocking her diseases, and making her feel inferior. He frequently ruins or cancels things that are important to her.He frequently hangs up on her, especially if she voices a slight disagreement or points out his lack of attention. Conflicts are constant, and everything is made into an argument. He controls their interactions, calculating time and blaming her for taking too long. He constantly changes rules and blames Ray even if she follows them.Ray notes similarities with her first partner in terms of misogyny but states that her first partner allowed two-way discussion, unlike Mohsin, who just silences her. Mohsin accuses her of being with him for money or cheating, despite evidence to the contrary. He also shifts his persona, sometimes appearing supportive and feminist, understanding her pain from society and doctors. However, he rarely exhibits this side. Previously Ray had thought he was not aware of the damage he was doing to her, but Mohsin recently admitted he "*knows exactly what he's doing*" and will *"amp up the speed now"* to destroy her mental health deliberately, because of his perceived insults from her. He admits that he knows exactly how to hurt her efficiently and deliberately seeks to destroy her mental health.

- **Using her health against her:** Even as he is sporadically supportive and espouses feminist values at times, during their conflicts he defends misogynistic doctors who dismissed her suffering, adding to her trauma. He criticizes her intelligence, calling her an "*autistic retarded bitch*" after she disclosed her suspected autism, and degrades her physical appearance.
- **Threats:** He has given Ray **rape threats** and described in detail how he would kill her. He has also threatened to harm her family,and has threatened to rape her mother in front of her, to bring in ten men to commit a gang rape to her and her mother.
- **Sexual Coercion:** In sexual interactions, Mohsin uses Ray's health problems as a form of abuse, saying he "*can't even have sex with you properly*" making her feel horrible. Even when they are intimate, he claims to have sex out of pity for her once they are done, saying that he hated every moment , even though he appeared happy during the acts. This feels particularly hurtful to Ray. Ray feels she has no choice when he asks her to do something sexual she finds uncomfortable, like oral sex, as he begs and uses guilt ("*I did this for you, why can't you do this little thing for me?*") Exhausted at his constant insults and also partly as her libido has been affected by her medications, Ray has not been able to participate in any sexual intimacy within the last 6 months.
- **Control and Religion:** He refuses to let her speak and constantly changes the rules of interaction. He weaponizes his religion against her, accusing her of hating Muslims when she expressed concern for his health during Ramadan and COVID-19, asking him to not fast that year. Ray considers herself as a progressive person who has actively participated in protests against oppression of religious minorities, and felt hurt by such flawed accusations, She tried explaining that her only motivation for that request was her worry about his health, Mohsin blocked her on phone and social media , severing all contact with her for three solid months. He refused to visit her after surgery because it was during Ramadan, emphasizing that his religion comes first and Ray is not important to him.Mohsin is 30 years old and hasn't completed his MD. He refuses professional help but claims to have a personality disorder and extreme anger issues. Ray states that

she feels constantly afraid in the current relationship, and was also terrified during the abusive phase of the second relationship.

Impact on Mental and Physical Health

Ray describes her mental health as severely affected, having "*crashed*" after the transition in her second relationship. She is experiencing anxiety and depression. She is constantly anxious, highly alert, and feels she must "*walk on eggshells*" around Mohsin. She experiences distressing symptoms such as racing thoughts, sweating, restlessness, and traumatic flashbacks, which often compel her to contact Mohsin, perpetuating the abuse cycle. She also experiences nightmares related to the relationship. She admits to feeling a general sense of bleakness, misery and impending doom.

Ray suffers from severe physical health issues, including **endometriosis and adenomyosis**, which cause debilitating pain she has dealt with since age 10. According to her , her parents did not take her pain seriously. She further found the experience of dealing with the misogynistic medical industry severely traumatizing. According to her, over the years, the cumulative dismissal of her suffering from her parents as well as the medical establishment was more damaging and debilitating, possibly even more than the physical pain itself. While she never had a big group of friends, her current isolation is complete and absolute. She expressed frustration at her friends, who she had thought of as a place for solidarity and support. Despite their feminist credentials, according to Ray they would still judge her for her string of abusive relationships, and fail to see the years of trauma that probably underlies her bad decisions when it came to relationships. When she tried to express her gynecological problem and pain, according to her, her friends knowingly steered the conversation away from endometriosis and the systematic medical negligence that marks her case. She used the phrase that her friends *"Appropriated her pain"* and turned the conversation to the much more common Poly Cystic Ovarian Syndrome (PCOS), which some of them had, but in the process excluded her from the conversation, which Ray perceived as a betrayal, further undermining her agency and ability to share her pain. This has alienated her from all her friends, exacerbating her isolation. She does not even want to go back to them for further support. She has also experienced psychosomatic issues, including jaw pain and back pain, and struggles with tiredness, sleep issues, and decreased appetite. She feels isolated, invisible, and misunderstood by family and friends, further exacerbating

her vulnerability. This relationship also makes her feel becoming increasingly distant from a staunch feminist standpoint she always believed she held.

Ray struggles with immense guilt and shame related to her body, health, and general life situation. Her guilt and self -loathing about her dependence on Mohsin also stems from her seeing this inability as a betrayal of her core feminist beliefs. She has experienced suicidal thoughts and wishes for a painless way to die, noting that she avoids self-harm because she already endures substantial physical pain due to her illnesses. She also suspects she has traits related to the **autism spectrum**, which makes dealing with major life changes, such as moving, especially difficult.

Coping mechanism and Future outlook

Ray's primary coping mechanisms revolve around philosophical practices and isolation management. She has found that dabbling in the philosophy of **Taoism** (learned via YouTube) has been more helpful than previous counseling sessions. She attempts to apply Taoist principles by focusing on slowing down, being silent, being still, and avoiding reaction, which helps reduce her anxiety. She also uses meditation and spends time in nature to calm herself. Due to her health, she keeps social interaction and activities (like watching TV) to a minimum, as almost everything triggers her, and even talking to other people can sometimes feel draining for her. Exhaustion is a persistent feeling.

Looking ahead, Ray struggles to envision a future and does not "*see light at the end of the tunnel*". She believes that recovery and healing depend on her ability to leave Mohsin. She feels she urgently needs a supportive network of people around her before she can move out of her current abusive environment. She feels she lacks the energy to fight the system (such as initiating legal proceedings) due to the draining nature of her existing battles with health and abuse. Ray states that what keeps her going is the hope of experiencing those rare moments when Mohsin shows his sweet, understanding side, when her pain seems to melt away. However, even as Mohsin has told her countless times he is with her out of pity, which has been deeply hurtful to her, she has not been able to leave the relationship because she feels she is no longer in control of her own life. When asked about her future plans Ray was unsure as she felt all her energies were now just about survival and getting through one day to the next. She displayed many signs of complex trauma as discussed in the manuscript.

File S2: Case Study: Damini

Damini (31)

1. Demographics and Family Background

Damini identifies as a female and will be turning 32 in November. She is married, and her marriage will reach its two-year anniversary in December. She currently lives outside of her hometown (Delhi) for work and marriage. She is financially independent and lives with her spouse, though her mother has been living with them for the last three months. The estimated combined household income (from both her and her husband's stipends) is around one lakh per month. Both Damini and her husband are PhD researchers. Damini grew up in north Delhi and spent around 28 years of her life there. She comes from a Protestant Christian family, as her grandfather was a priest, and they lived in a parsonage within the church premises. Her parents had a love marriage, as her mother is a Hindu Khatri, though Damini personally *"does not have a religion"*. She has an elder sister and adopted her niece as her sister when her elder sister abandoned the little girl to elope with another man. The household was initially led by her grandfather, who was a patriarch. As a teenager (around age 12 or 13), Damini became *"a rebel"* and began *"speaking against the problems and the atrocities that my grandfather inflicted on my mother"*. Conflict arose because her mother, a practicing Hindu, was married into a Christian priest's family and was not allowed to practice her religion openly or keep a small temple at home. Furthermore, her pure vegetarian mother *"was forced to eat non-vegetarian food,"* and if she didn't comply, she was ridiculed. Damini herself was called a *"Hindu kali"*. This stand led to estrangement from her father and grandfather. When her father passed away when she was 17, Damini took up major responsibilities, including managing hospital bills, which repaired her relationship with her father. Following his demise, the family suffered severe financial problems. Her mother went into *"serious depression,"* stopped cooking and speaking, and developed a bad relationship with Damini. She said extremely hurtful things to Damini such as *"Your father died. Why did you not die? I wish you had also died."(Excerpts in Appendix 8A, I6)*

2. Career

Damini started working at age 17 to support her education and responsibilities. She worked as a young child reporter/coordinator through a fellowship from the Gandhi Smriti and Darshan Samiti (Ministry of Culture) and conducted projects with UNICEF and FAO. She is proud that *"there hasn't been a bridge in my career"* and finds that working keeps her *"very happy and grounded"*. She currently takes *"the major decisions in my house"* (marital home) and financially supports her natal family (mother and niece). Damini is currently pursuing a PhD at an Indian University, specializing in modern Indian studies/history. She holds an M.Phil and she started working at age 17 while attending St. Stephen's College for her undergraduate degree, due to the family losing funds after her father's death. She has over a decade of work experience and has not taken a break in her education or career. While in her M.Phil., she taught ad-hoc classes in history and sociology for 11th and 12th grades. Before her PhD, she was a teaching fellow at a private university in the outskirts of Delhi, for three and a half to four years. She attributes her continuous career to keeping her *"very very happy and grounded"*.

3. History of Romantic Relationships

Damini states her history of romantic relationships has been *"very problematic"*. Starting her dating life around age 18 in college, she admits that her subsequent choices were driven by a profound *"neediness"* for emotional support. She confesses, *"I have a very difficult time being alone without a relationship"* and always *"craved to be loved"*. This emotional vulnerability caused her to ignore obvious *"red flags"* in relationships she *"should have never gotten into"*.

a. Past relationships

She started dating at age 18 (second year of college). Her first relationship, with her close best friend, lasted about four to five years and ended because he cheated on her multiple times. She continued the relationship for another year because she was "*not ready to live without him*". Her first relationship lasted *"about four five years"* and ended when the partner repeatedly "*cheated on me*". After this, she fell into a series of five subsequent relationships that were consistently *"abusive and exploitative"*. She was drawn to these abusive dynamics because she was *"very needy" f*or emotional support and love and found it difficult to be alone without a relationship. The men she dated were *"toxic and extremely controlling"* and patriarchal, affording her *"very little*

autonomy". They frequently lied, and she noted that while she was a staunch feminist, she consistently ended up with men who turned out to be *"very patriarchal"*.

b. Current relationship

Her current marriage is an intercaste, interreligious love marriage to her partner, Bharat. They knew each other for 6 to 8 months before deciding to marry. Bharat is 36, an engineer, who is now pursuing a PhD from the same university as her. His job is considered more stressful than hers because he has to do teaching assistantships every semester throughout his PhD. He devotes *"very little"* time to his family, mostly through phone calls. Damini's relationship with Bharat is "*becoming better now*" since she began therapy. Bharat was diagnosed with bipolar disorder before the marriage, a condition that runs in his family, which he manages primarily through Vipassana meditation. Although Bharat generally takes care of her, the marriage suffered for about one and a half years due to issues with his "*very regressive*" and patriarchal family. He has never criticized her intelligence or physical appearance. They both have independent access to their stipends and spend their money as they wish, without interference. They make major financial decisions together. Her husband has never pressured her for sex or ignored her discomfort regarding sexual acts. He doesn't keep tabs on her when she goes out alone and has never humiliated her in a social setting.

4. Abuse Histories

a. Abuse in natal family

Damini grew up witnessing the "*atrocities*" her grandfather inflicted on her mother due to her mother being a Hindu married into a Christian priest's family. Her mother was prevented from practicing Hinduism openly (not allowed a temple or to go to the temple). Despite being a vegetarian, her mother was ***forced to eat non-vegetarian food*** and ridiculed if she did not comply. Damini herself was subjected to prejudice and called a ***"Hindu Kali"***. When Damini spoke up against her grandfather (age 12 or 13), her father became angry and "***estranged me completely***" for about a year (from the eighth to the ninth standard). She only started talking to him following his first heart attack, when she assisted her mother with his care, and coordinated with the doctors for medications etc.

She also suffered extensive family trauma. Her father passed away when she was 17 and her mother went into *"serious depression"* afterward, stopped speaking normally, or cooking, developing a *"tumultuous relationship"* with Damini and saying things like, *"Your father died. Why did you not die? I wish you had also died"*.The family was socially ostracized as they continued living in the same house, which the church wanted to reclaim after her grandfather's death, for it was supposed to be a priests' accommodation. Her family faced a decade-long court case filed by the church on grounds of *"encroachment of property"* adding to their financial difficulties. Her mother was also diagnosed with cancer shortly afterwards. Her elder sister abandoned her child (the niece) when Damini was in class six. Starting at 17 years of age, through her 20s, she had to be the sole provider for the family with so many adversities on a meagre income , making her feel *"socially, financially, emotionally very vulnerable all the time"*.

b. Abuse in marital/cohabiting relationships both by partners and their family

By Past Partners: Her previous partners were extremely **controlling and patriarchal**, leaving her with "*very little autonomy*". Abuse included:

- **Verbal Abuse and Sexual Degradation:** She was frequently lied to. She was called a "***prostitute***" by multiple men, and was asked her price, specifically, "*How much do you charge for your vagina?*". They ridiculed her English pronunciation, intelligence, and physical appearance (e.g., told she looked like a "*Bhains*" which means a buffalo, that her hand or feet were not dainty and beautiful, as one would expect of any woman), all of which were severely damaging for her own confidence, self esteem, and body image. Another partner suggested she must be earning money through sexual means because she had financial responsibilities: *"must be that men are you know visiting you in some way or the other"*.One partner would call her at specific times, making her life *"hell"* if her phone was busy. They also degraded her family, especially her mother and niece, being *"very insensitive"* and insulting. Despite this, these experiences paradoxically strengthened her relationship with her family, as she realized her family was her constant support.
- **Manipulation, Blackmail and Threats:** Partners used lies, pretense (*e.g., one pretended to be bipolar, having survived a fall from a high floor*) to gain sympathy and manipulate her, or using guilt to subjugate her during disagreements. Conflicts often ended with her pacifying them. One partner used **threats of self-harm**

(claiming he would jump from a cliff) to induce guilt when she tried to break up. She was **threatened, warned, and blackmailed many times**. Partners used their social position (e.g., Indian Engineering Services) and her family's financial vulnerability to scare her, threatening to send the police or CBI to her house or inform her university department. At this time she was only 22-23 years old and gullible enough to spend days in abject fear from these threats, believing the lies consistently. When she tried to end the relationship, he threatened her, leading her to contact the women's police. She ultimately informed his mother and ended all contact, though he continued to send *"very bad emails for years"*.She also felt physically threatened in relationships, fearing they would hurt her, even if not directly hit. This cycle of fear happened with multiple partners.

- **Gaslighting and Isolation:** Partners dismissed her feelings, calling her "*crazy*" or "*irrational*". They degraded her mother and niece. One told her she needed him for "*refuge*" and that no other man would marry her because she was isolated. One man specifically told her she was isolated and dependent on him: "*I'm the only one who can be with you... There's not going to be any man in this world trying to get married to a girl like you... So the only person whom you can come for refuge is me*"
- **Coercion/Sexual Abuse:** She was forced for sex even when she was not in the mood. On one occasion, a partner closed all the doors and forced physical contact, and on another, she was forced to drink heavily, leading to non-consensual contact. She was also manipulated into sexual acts she found uncomfortable. Her partners were "*very very insecure men"* and controlled her appearance and movements, criticizing her for wearing a sari because *"other men are going to look at you"*

By Current Partner and In-Laws:

- **Marital Conflict:** During severe fights, both she and Bharat have been so angry that they started **hitting their own selves,** but never each other. Bharat has shouted at her, causing her to feel disrespected and humiliated. He has also ignored her feelings or told her they were irrational.
- **Husband's mental health:** Bharat is diagnosed as bipolar. The condition runs in his family, as his mother also suffers from serious bipolar disorder, as does his brother. He manages his bipolar condition "*without any medication"* by practicing Vipassana meditation. However, Damini expressed anxiety about Bharat's health

concerns, as she herself was not helped by Vipassana at all, instead feeling like her feelings were being ignored and she was some *"lab rat"*. However, chronically traumatized individuals often can't benefit from meditation because of their chronic autonomic deregulation, which would take much more time and training before the beneficial effect of mindfulness can truly be felt.

- **In-Laws' Abuse:** Bharat's family is described as "*very regressive*" and "*very patriarchal*". She describes them as *" a middle class Baniya family"* indicating their upper caste merchant status, signaling they had access to wealth. But she also describes them as regressive and their lives just revolving around *"food and kids and festivals and marriages."*
- They caused a "mess" in the marriage for almost 1.5 years. They showed open disrespect to Damini's family, such as *wilfully not inviting her mother to the wedding reception*. She did not speak to them for a while after this. Her in-laws also physically took back "*bills*" (belongings or gifts) that they had given her for her wedding. Damini noted that Bharat struggles to stand up for her against his family, which caused her a lot of anguish. She however attributes it to his disciplinarian small town upbringing where protesting against an elder while defending his wife would be inconceivable. Ultimately, Damini eventually had to *"put her foot down"* and *"shout at his family,"* which changed the dynamic as they had never heard a woman speak like that.
- **Unequal distribution of household chores:** Damini feels she has more responsibilities at home that affect her work, particularly cooking. Since her mother started living with her, the amount of cooking has increased, and her husband *"doesn't enter the kitchen that much,"* which is a source of fights. She feels her responsibilities are always greater, as her husband, having grown up in a family that didn't teach him responsibilities, doesn't cook for himself even when alone. He contributes *"little bit"* to housework only after she fights with him. They do not yet have any hired house help as is quite common in India.

4. Impact on Physical and Mental Health

Damini noted that she was feeling slightly unwell at the time of the interview, mentioning she was having *"slight fever and cold"*. Damini's mental health struggles began in college, though she was unaware of the seriousness at the time. She acknowledges that her depression started when she was in college, but she was "in

denial" and never sought follow-up care, partly because the word *"depression"* was *"very taboo"* in 2007. She felt she had to live up to the image of being *"very strong,"* a tag that felt like a *"dead albatross"*. Damini sought therapy about a year ago, and was recently diagnosed with **Post-Traumatic Stress Disorder (PTSD)**, which she believes stemmed from her history of relationship abuse. Prior to receiving treatment, she was in a *"very bad shape"*. Symptoms included crying for hours, sleeping 15-16 hours at a stretch, and experiencing very severe panic attacks at times. The situation was so grave that she was actively **planning her death** and looking for ways to end her life. She also felt intensely lonely and vulnerable after each failed relationship.

5. Coping mechanism and Future outlook

Damini considers herself a resilient person who "*bounces back after every fall*".

Coping Mechanisms and Support Systems:

- **Professional Help:** She sought help from a psychotherapist and a psychiatrist (consulting POS for Perspective) and is taking medicine regularly, which has helped her get "*on track*" and feel "*much better*".
- **Resistance:** She "*fought back*" in all her relationships. She actively fights back against her husband's gaslighting, lecturing him and making him realize his error. She took a strong stand against her in-laws, shouting at them and telling them their behavior was unacceptable, which "*changed the game completely*".
- **Social Support:** She maintains a large social circle and a strong support system of friends (including her PhD cohort) and family (mother and niece).
- **Marital Efforts:** Bharat initiated the move to seek separate and couples therapy sessions. This has led to a drastic improvement of their conjugal relationship, and communication. Immediate conflicts are resolved through apologizing, hugging, kissing, and making love.

Despite the constant attacks on her self-worth, her self-esteem was **not ruined** because of her resilience, and knows she is *"much better"* than her abusers. She also notes that all the negative labels her abusers used to strip away her confidence only exists for women rather than men, when the burden of guilt largely resides with them. In her own words, therapy helped her to learn to *"love myself thoroughly"*. Since starting medication and therapy, Damini reports a significant improvement in her mental state. She feels she is

"on track and I'm much better". She now gets up *"happy"* and *"sleeps well"* and on time. She noted that the Bharat's willingness, who initiated couples therapy, to work on their relationship has also helped improve her outlook towards life and made her feel "*less vulnerable*"

Future Outlook and Wishes: She feels "*so much better now*" due to therapy and medication, noting she gets up happy and sleeps well. She feels less vulnerable and more confident in others. Her in-laws do not live with them and generally behave well with her now that she is more vocal about her feelings. Her primary wish is for Bharat to stand up for her more against his family's regressive and petty behavior so that she does not have to constantly fight with them and deal with the "*mess*". Damini explicitly states that while her in-laws expect them to have children, she does not want to have children, and that she finds the thought of having children scary. She notes that she still struggles with feeling guilty when doing something solely for herself, a feeling developed from being the sole bread owner for her family for so long.

<u>File S3: Case Study: Renu</u>

Renu (31)

Demographics and Family Background

Renu is **31 years old** and identifies as a **Brahmin living in Hyderabad**. She grew up in a **joint family**. Her childhood is described as being filled with **really great memories, a happy one**, and **full of energy**, with lots of family around and playing. She confirms her childhood was **not unhappy or stressful**.

Her relationship with her parents was "*really great*" and they were *"more supportive"*. She still maintains a *"strong bonding"* with them. She characterized her early life as being *"like full of bed of roses"*. Her father, who was in the army, was described as a *"cool guy,"* and she was never *"pressured to follow any norms or any rules"*. Instead, she was given her *"own choice to lead my life"*. Crucially, in this joint family setting, she reported that while differences of opinion existed, they were *"not that major conflicts that the family breaks"* and never reached an extent that *"would really affect the relations"*. Renu consciously notes that she was *"only exposed to one side of it where it is full of happiness, comfort, great relations, and bonding"*. This supportive environment led to an insulated social life; she described herself as *"bit kind of an introvert"* who takes *"time to open up to people"*. She had *"very very few friends apart from family"*, explaining that she *"nearly never felt a need of a friend outside"* because she *"had a gang of army at my home"*.The core critical observation of Renu's background is that this extreme protection and autonomy created a significant vulnerability, causing her immense difficulty when confronted with abuse.Having been exposed only to the beautiful, good *"one side of the coin"*, Renu realized later that she *"was not ready for that"*. She noted that she learned the hard way that *"man is always like that no man can be a horrible side as well"*. Her established identity as an *"independent and happy go girl who speaks our mind out and no rules"* directly clashed with the expectations placed upon her in her marriage. The emotional draining she suffered stemmed from being forced into the role of a *"so-called married women"* expected in *"Indian society"*, involving mandates like being told to "*sit in a home just four walls"* and having to *"resign my job"* and *"give away a few projects"*. Her career was *"ably stopped"*.

Career

Renu established a strong professional identity built on independence and ambition, which aligned with her autonomous upbringing. She was an *"educated"* and *"working"* individual prior to her marriage. Her history positioned her as an *"independent and happy go lucky girl"*. The most critical impact on Renu's career occurred during her two-year abusive first marriage (entered at age 25, dissolved by 28) with Ganesh. One of the sharpest losses that Renu remembers doing substantial damage to her was the deliberate and forced termination of her professional life, despite promises to the contrary when marriage was being arranged. She could not understand this, as both her in-laws were educated, and high ranking government officials. She was forced to *"resign my job"* and *"give away few projects"*. This was part of a rigid, patriarchal expectation that she become a *"so-called married women"* who had to *"sit in a home [within] just four walls"*..After leaving the abusive marriage with Ganesh in 2017, Renu had to restart her professional life, feeling like she was beginning a *"second innings or like second new beginning"*. She credits her mother for heavily encouraging her *"to get back to my studies and to my work"*. She subsequently focused on her education, currently pursuing her PhD from a university in northern part of India. She describes the experience as something that "*affected my confidence*" and "*affected my thought process*". She noted that the fears and anxieties she developed were *"new things"* that *"really affects my performance and when I'm on stage"*. The experience of those *"four years of life has totally changed"* her. She feels she has *"evolved to a total different personality"* and is no longer the *"24 years girl who was like in a different world"*. This shift means she interacts with professional colleagues differently, sometimes shocking her friends and cousins with her changed perspective. In current professional or academic disagreements, she *"tries to make my point for sure"*. However, if the point is not accepted, she will "*quietly draw back"* and not *"come out and again fight for it"*. This retreat, while indicating self-control (she doesn't "overreact"), suggests a tendency to avoid prolonged conflict, which she questions herself, noting that running away is *"not correct"* and *"affects that you are just running away from it"*. She specifically mentions her current work in higher studies, which often puts her in rooms where her peers are engaging in academic debates, and she sometimes feels disturbed and unfocused during these sessions, feeling extremely uncomfortable. In conclusion, Renu's career history demonstrates her inherent capacity for independence and work, which was violently suppressed during her first marriage. Although she has successfully returned to professional and academic pursuits, the experience left a critical, enduring impact on her confidence and overall professional persona.

History of Romantic relationships

a. Past Relationships

Before her marriage, Renu's romantic history appears minimal or non-existent. When asked how old she was when she entered her first romantic relationship, she responded, "*I didn't had any romantic relationship so far but yeah uh nothing of that kind. Yes*". This lack of prior experience, coupled with her highly insulated upbringing (which was *"like full of bed of roses"*), critically contributed to her unpreparedness for the abusive environment she entered.

b. Current relationship

Renu was married at age 25 (or 24/25) through a family decision, and by age 27 she had *"walked out of the marriage,"* officially divorcing at 28. She described this two-year period as *"terrible"* and leading to *"after effects of that trauma still with me now"*

Abuse History

While Renu experienced physical violence, she stressed that the abuse was predominantly non-physical and psychological:

a. Abuse in natal family

Renu reports that her childhood was happy, comfortable, and supportive. She was **never used to people talking in high tones** and was not raised with strict rules or pressure. There is no indication of abuse within her natal family.

b. Abuse in romantic and marital/cohabiting relationships

Renu was married at age **25** through a family decision, walked out at **27**, and was divorced by age **28**. The marriage lasted two years.

Renu experienced **physical violence** during this marriage. She confirms that episodes of **physical violence took place twice or thrice** during the duration of the marriage. Examples of physical assault include her husband pushing or beating her, and pulling her by the hand and **dragging her out of the house enacting throwing her out of their home, to shame her publicly** .

The abuse was **more than just physical**; it was **emotional, stressful, and threatening**. She found the experience **emotionally draining**. She experienced **humiliation, all day, every day**. This humiliation was sometimes done publicly or in front of guests. She describes the goal of the humiliation as bringing her down to the core so that she would **lose herself badly**. Other forms of abuse and control included:

- **Isolation:** The in-laws and husband **made sure that she was not in touch with the outside world**. Stopping her from working outside the home contributed to this. She reported her mobile phones were frequently *"broken off"* by her husband, who *"made sure"* that she stayed isolated. This isolation prevented her from maintaining professional contacts or seeking external support. She was not able to communicate with her family from the in-laws' place. She had to resort to writing a brief email to a cousin for rescue, fearing for her safety.
- **Destruction of Property:** Renu's phone was often the first thing to be destroyed by her angry husband. She purchased **five to six mobiles in that two years just because that phone used to get broken off**.
- **Enforcement of arbitrary and petty rules, followed by threats:** Her husband threatened to **throw her out of the house** multiple times. She recounted an incident where her husband pulled and forcibly dragged her out of the house shouting at her, because she forgot to set an alarm for him. She reflected that him missing a workday would worsen the abuse on her, if he stayed home for the entire day. He would throw whatever food she cooked away and these patterns of abuse continued constantly. On other occasions, she remembered that he would throw food that she cooked, and threaten to leave her if her behaviour was not to his liking, on extremely petty grounds. .Her father-in-law was never physically abusive directly, but *"very threatening," "creating a constant sense of "surveillance"* and intimidation. Renu reported that the constant stalking of her movements and communication from her husband, and the general sense of threat of violence loomed large in her head throughout the marriage and this was what felt worse than the physical violence that she was subjected to. These threats, combined with her unfamiliarity with such harsh treatment from her supportive upbringing, solidified her decision to leave: *"I decided [before] you're leaving me rather I kick you off and go out of the house"*

- **Sexual Coercion:** Renu reports that **multiple times** she was pressured to engage in sexual activity when she was not ready, causing her to lock herself in a room to protect herself.Renu implied facing coercion stating that *"multiple times yes"*, she was *"not ready"* and tried to *"just run away from it,"* often locking herself in a room to *"save myself"*
- **Public Degradation:** She was never allowed to talk to guests in the household. She was expected to just open the door, and then recede into subservience. If she dared to talk to them on equal terms, she was sharply rebuked, reminding her that she was not good enough to talk to her husband's family. She recounted a horrifying incident where her father-in-law (who was "*very threatening*") shouted at her in public as she tried to flee the house in her nightwear. He yelled a humiliating comment that she "*look[s] like a lady who just* [got] *out from the bed,*"calling her an "*illicit lady*" causing her great anger and shame.
- **Control over Social Interaction:** She was not supposed to talk to guests beyond a "formal welcome greeting". Any perceived self-importance led to humiliation, such as being told she was "*just family member don't be so important for yourself you become your family like my family. It's never been our family. It's my and yours"*.
- **Explicit threats of abandonment and physical removal from the home:** In one instance,Ganesh *"pulls me with his hand and drags me out of the house"* because she forgot to set his alarm causing him to miss his transport to office, threatening, *"if you behave like this again I'm going to throw it off you from my house"*. Renu also mentioned that one time he also threw food and threatened, *"I'm going to leave you"*. Renu used these threats to solidify her decision to leave: Renu lived with her in-laws, who were educated and held high positions. However, **they provided no support**. She tried speaking to them,believing they should be *"responsible for [their]the kid, how he is behaving*". However, **the severity and the intensity [of the abuse] was even more increased** afterward, making her feel no point in talking to them. Her in-laws also contributed to the **constant surveillance** on her. This complicity forced her decision to "*just go back and talk to my parents and yeah and that's what I've done"*

Renu managed to escape by writing an emergency email to one of her cousins, saying: ***"Please pull me out... I'm not sure how safe I am."***. The moment she told her parents, she was out **within a week** due to the ***"great support"*** of her family.

Curiously enough, Renu's family had to devise an elaborate ruse to get her out of her marital home, concocting a story that an elderly relative was on her deathbed, and Renu had to go meet her. Renu and her cousins all feared that she would not be *"allowed"* to leave the house otherwise. While the support offered by her family is undeniable, this also shows how a marital home's decision to restrict their daughter-in-laws mobility outside the house is legitimized, and socially accepted.

Impact in Mental and Physical Health

The two years of marriage were **terrible**, and Renu **still has the after effects of that trauma**.

She suffers from intrusive thoughts and experiences which she refers to as "***flashbacks***" or "***episodes***". For her, this means she is " ***again reliving and everything comes before me***". She specifically recalled going through the feeling of her husband dragging her out of the kitchen during one such episode.

After returning home, she began dealing with **anxieties** and **fears** that were not present earlier. She sometimes felt these issues when traveling alone, noting that she would ***"freeze in the middle of the road"*** due to a loud sound. She finds she gets easily disturbed and cannot take certain things which were normal for her before. She particularly mentions being very uncomfortable with noise, loud sounds, and any form of confrontational situation. While her family is mindful of these situations, it is plausibly impossible to regulate any of these as she resumes working again.

The abuse **affected her confidence** and **thought process** significantly. She feels she has "***evolved in*[to] *a totally different personality"***; the person she was for the first 24 years of her life is gone.

While Renu never felt an *"out of body experience"*, she noted that for a couple of months after she came out of the marriage, ***"I was not myself"*** and she ***does not remember what really happened*** during that time. **Coping mechanism and Future outlook**

Renu utilized several mechanisms to cope and recover:

- **Family Support:** Her family, including her parents and cousins, provided **great support**. They became cautious not to make noise , even regulating the amount of conversations around her, or play loud rock music around her initially, recognizing she was not herself.

- **Professional Help:** Renu **sought professional help** after leaving the marriage in 2017 and is **still in touch with them**, though she is not currently on medication. This professional help, alongside her guru, **helped her to face the legal process**—including dealing with police, lawyers, and courtrooms—and gave her the strength to face the grilling questions and justify her decisions.
- **Spiritual/Meditative Practices:** She practices *tenner yoga* and *mandelas*, and **meditates**. She maintains contact with her guru, who taught her techniques to maintain calm and heal herself.
- **Emotional Regulation:** She is not grounded and stable all the time, even now. When she feels she is losing stability, she makes a call to her **mom** or her **guru** to **vent it out**. She keeps herself busy with work and deadlines to avoid reflecting on negative emotions. Renu states that she tries to **respond to people, not react**.When she is upset, ***"water comes out of*** **[her]** ***eyes"***, without her being able to control them. She is also prone to panic in stressful situations.
- **Conflict Handling:** Renu reported being extremely uncomfortable in conflict situations and finds herself very wary of them. In disagreements, Renu tries to make her point with **logic** in a **softer voice**, attempting to convince people twice; if not agreed upon, she **quietly draws back**. She no longer fights for her convictions as before, or raises her own voice at all.

Renu expressed **pride** in herself for raising her voice and getting support. She recognizes that the experience taught her deep insights into the "gray shades" of individuals. She sees her life post-divorce as a **second chance** or **second beginning**.People close to her observe her overall transformation and "*want the old Renu to be back,*" but she states she is "*alive. Just be happy for that. Don't... search for the 24 years girl who was like in a different world*".

File S4: Case Study: Priya

Priya (50)

Demographics and Family Background

Priya is 50 years old and identifies as female, currently residing in Kolkata. Priya grew up primarily in Kolkata, spending some time in Delhi. She lived in a household of five, including her "*elder sister and my grandmother and parents*". She reported that there were no major conflicts "*at all*" between her family members while she was growing up. Furthermore, she was shielded from domestic duties, stating she "*never*" took major responsibilities at home. She is highly educated, holding a postgraduate degree and a B.Ed. She is currently single and divorced. She has one 21-year-old daughter. Priya is financially independent. Growing up, there was **no conflict at all** between family members. Her father was the primary decision-maker and also controlled the finances. Growing up, while the environment in the household was good, the household operated under clear paternal authority. Her father held the "*most decision-making power*" and was "*most(ly) in control of the finances*". Her relationship with her mother was close and friendly, while her relationship with her father was described as more or less formal. Priya states she never took on major responsibilities at home when she was younger and notes that her ambitions were modest, adhering to the "*normal typical Indian*" expectation of either getting a job or getting married and settled in domesticity. There is no history of psychological illness reported in her family. .The central critical point of Priya's background is that the "*very open-minded*" nature of her natal family directly was at complete odds with the "*very conservative kind of family*" that she was married into This cultural dissonance between her liberal upbringing and their conservative expectations became a common reason for marital conflicts. She separated after 3 years of marriage when her daughter was born, and came back to her natal home. She obtained her divorce after 15 years of separate living, Currently, her immediate family consists of one person, her father. Both she and her father, who is a pension holder, are earning members, with a reported monthly family income of around 130,000 to 140,000. Priya now takes most of the major decisions in her house and financially supports her family.

Career

Priya is highly educated, holding a postgraduate and B.Ed. degrees. However, her professional ambitions growing up were notably modest, conforming to traditional

societal expectations. Critically, her higher education level—being a postgraduate while her husband was *"just a graduate"*—was used against her. Priya was only able to return to professional life after she separated from her husband. She began working again after the birth of her daughter when she separated from her husband. She is now employed as an assistant teacher at a school, working five days a week. She reports having 19 years of work experience, indicating a long and continuous career trajectory outside of her brief, oppressive marriage. She is now financially independent and contributes to a high household income (around 130,000, 140,000 monthly) alongside her father's pension. This financial autonomy is reflected in the fact that she "mostly" takes the major decisions in her house.

1. **History of Romantic relationships**

a. Past Relationships

Priya's first and only reported romantic relationship was her marriage. She had no romantic relationships prior to her marriage. She entered her first relationship (marriage) at around 24 or 25 years old. The marriage was arranged, and she had known her partner for two months before getting married. It was an intercaste marriage, but it had the full approval of both families.

Priya and her husband separated after three years, citing a "*Clash of egos*" as the reason. Legally, however, the divorce was finalized 15 years later. Her ex-partner is 56 years old, a graduate with a specialization in marketing, and a businessman. His family was described as "*quite rich,*" richer than her natal family. During the marriage, her husband made all the financial decisions. She reports that her husband was not supportive and put zero effort into caring for their daughter. During the three years they lived together, Priya was subjected to severe, total control, which stripped her of autonomy and independence. She stated she was "*totally controlled by him*". Her in-laws did not help her care for her daughter, as she separated and moved to her parental home immediately after the birth. Her relationship with her in-laws was described as superficial and formal.

After the end of the marriage, it took Priya about **5 years to recover**.

b. Current relationship

Priya is currently single and divorced. She has **absolutely no contact** with her ex-partner. She took sole responsibility for their daughter after separation, acting as a single mother, and her ex-partner was not a part of the daughter's life.

2. Abuse History

a. Abuse in natal family

Priya reported that there was **no conflict at all** in her natal family while she was growing up. She noted that the arguments in her parental home were not characterized by the kind of shouting attitude she later experienced in her marriage.

b. Abuse in romantic and marital/cohabiting relationships

During her marriage, Priya felt **totally controlled by her husband**. She reports high levels of conflict due to the clash between her open-minded upbringing and her marital family's very conservative nature.

Emotional abuse and humiliation: The abuse started in the first few days of the marriage when her husband began to shout at her. This behavior was later repeated in front of other family members who were *"non chalant"* about it. Her husband humiliated her, especially in front of his family. If she expressed an opinion, he would say, *"Don't think that just because you have a post graduation doesn't mean you know everything"*. Priya understood that her husband did this to feel superior because she was more educated than him. Her husband also ignored her when she was angry or hurt, making her feel like her feelings were irrational. She felt **horrible and terrible** when he verbally abused her.

Gaslighting, attribution of blame and Guilt: Priya's husband *"always"* made her feel that his abusive behavior was her fault. She admitted that, being *"a little naive"* at the time, he *"did make me feel guilty,"* leading her to question if she *"shouldn't have done this or I shouldn't have said this"*.

When she felt angry or hurt, he would "ignore" her, rather than take accountability or try to mitigate the conflict. This silent treatment was used as a form of gaslighting, to make her feel like her feelings were irrational.

Lack of affection, Emotional Neglect and invalidation: He was indifferent to her emotions, making her feel like her feelings were irrational or *"crazy,"* simply by ignoring her. He took no care of her emotions or her happiness. While her husband

had his own social circle, he never made any attempt to include Priya. She could not remember any instance of them going out as a couple, or a vacation. She felt she was completely uncared for, and excluded from any decision making, her wishes and opinions entirely discarded.

Control and Isolation: Priya was not allowed to have a social life outside the marriage. She was not permitted to go out alone unless it was to her parents' place or the market. For the three years she lived with her husband, despite her postgraduate education, her career was entirely halted due to the conservative and controlling nature of her marital household. Priya was *"not allowed to take a job"* during the marriage. She was expected to fulfill the role of a *"typical Indian bahu"* and was treated as a *"showpiece"* by her husband's family. Her in-laws were also *"not supportive"* of her working..Priya rarely socialized and did not have many female friendships, and a single male friendship during her marriage, so situations involving her husband's reaction to her talking to other men did not arise.

Physical Threat: Priya never experienced physical violence, but she did feel physically threatened in instances when her partner broke things in the house (which did happen, though not frequently).

Sexual Coercion: Her husband pressured her for sex even when she was not in the mood. She navigated these situations by being submissive. He also made all decisions regarding contraception and their sexual relationship.

Financial abuse: Her husband made all financial decisions, and she had no access to her own money, forcing her father to secretly provide her with *"pocket money kind of a thing"* for her own expenses.

Parenting and Caregiving: Priya eventually decided to have a child; she stated, *"I pressurized him"*. However, the husband put *"zero"* support and effort into taking care of their daughter while they were married. After separation, co-parenting did not occur, and she became a single mother, noting, *"it was my responsibility"*. Her ex-partner reportedly said that he was *"not a part of your daughter's life"* after separation. She noted that while married, she was *"entirely there"* for her in-laws, caring for them *"like a true bahu" (A real daughter-in-law).*

Impact in Mental and Physical Health

The romantic relationship experienced by Priya, which was her three-year marriage, resulted in significant negative impacts on both her physical and mental health due to recurring conflict, emotional abuse, and control.

Mental and Emotional Health Impacts

- **Emotional Suffering and Despair:** Priya noted that to resolve conflicts, she often *"used to stay quiet"* and would ***"suffer a lot within myself"***. The first time her partner shouted at her, she described the feeling as ***"horrible, terrible"***. Even when the shouting incidents recurred, she felt ***"very bad"***, often responding by keeping quiet and retreating to her room. She also routinely ignored the fact that fights had occurred, rather than addressing them.
- **Stress and Worry:** During the marriage, Priya stated that she felt ***stressed*** when she woke up in the morning. She worried about the relationship, specifically thinking about ***"how the day will be, any new issue would crop up or not"***.
- **Guilt and Self-Blame:** Priya was ***"always"*** made to feel by her husband that his abusive behavior was her fault. She described being ***"a little naive"*** at the time, which led her husband to ***"make me feel guilty"*** such that she questioned if she ***"shouldn't have done this or I shouldn't have said this"***. This feeling of guilt continued after the relationship ended; initially, after separation, Priya stated, ***"I did blame myself"*** and felt guilt, thinking ***"maybe I could have stayed there"***.
- **Fear and Threats:** Although Priya stated she ***"never experienced physical violence"***, she did ***feel physically threatened.*** There was an instance where her partner ***"broke things in the house, such that [she] was afraid"***.
- **Fear of Society:** A major source of anxiety that prevented her from leaving the relationship was a fear of ***"the society"***. She stated that this fear was the ***"main thing"*** in her mind.

Physical Health Impacts

The mental stress associated with the relationship led to clear physical manifestations:

- **Weight Loss:** Priya noted that the mental strain affected her ***"very much,"*** and this resulted in her ***"losing weight and all"***.
- **Appetite and Sleep:** Her "**Appetite**" was affected significantly. . Furthermore, her **sleep** was impacted, and she recalled ***"taking sleeping pills at that time"***.

It is also noted that her ex-partner may have experienced mental health challenges; Priya had heard he *"**had been to some kind of counsellor for counseling**"* after marriage, though she was *"**not really sure of the exact, you know, diagnosis**"*, which also demonstrates the extent to which she was excluded from her husband's life. Despite the long recovery time (approximately five years) and initial guilt, Priya noted that she is now financially independent and does ***not*** feel guilty at all if she takes time out for herself.

3. **Coping mechanism and Future outlook**

Coping Mechanisms During Marriage: Priya's primary coping mechanism was **staying silent** to avoid escalating conflicts while suffering internally. . When her husband shouted, she would keep quiet and retreat to her room. She also routinely ignored the fact that fights had happened. Regarding sexual coercion, she reported being submissive. She only reported the abuse to her own family after it *"went on and on"*.

Current Coping and Outlook: Priya is now financially independent and works as an assistant teacher. She has hired a domestic helper as well to assist with household chores. While she initially felt guilty when taking time out for herself, she reported that she **does not feel guilty at all now** if she takes time for self-care. She is divorced and has absolutely no contact with her ex-partner. She took five years to recover from the relationship's end.

File S5: Case Study: Meera

Meera (56)

1. Demographics and Family Background

Meera is 56 years old. She holds a Master's degree and currently resides in Kolkata. She grew up in Bilaspur. She comes from an educated background. Meera grew up in her natal family with five sisters and her parents (seven members in total). Meera is the youngest of the five sisters. Meera described her upbringing as a "*very happy life,*" noting that while they faced some financial crunches, there were "no mental issues". The family was described as a *"very well-knit family"* with *"never"* any conflict when Meera was growing up.

In her natal family, her mother exercised the most decision-making power. Her father, along with her elder sister (once she started working), controlled the finances. After her father expired, Meera took on major responsibilities, including taking care of her mother and her health. Meera stated that she sometimes felt burdened and had to compromise, possibly because she was the youngest of the five siblings. Meera's natal family has a history of psychological illness; her mother had a history of depression, and her eldest sister also suffered from depression due to financial problems, though she recovered with medicine.

She does note that if her childhood situation was somewhat like her own daughter, she could have done so much more for herself. She notes that while they lacked for nothing as she was growing up and never had to borrow money from others, they did not have a lavish lifestyle and could not afford luxuries, noting that families these days brought up kids very differently. She thought the way she had brought up her own daughter was very different from her own relatively frugal upbringing. She also notes that while her family was considered quite progressive, and she as well as some of her other sisters' were encouraged to educate themselves and work as well, their upbringing was more conservative in terms of freedom that girls have in modern times. She does note that her family was nothing different from other families in their circle in this regard and that *"everyone was brought up that way back then".*

Meera is currently married and has been for 30 years. She has one child, a 27-year-old daughter. Meera, her husband, and her daughter currently live together, forming her immediate family.

2. Career

Meera's current employment status is that of a high school teacher. She has more than 35 years of work experience. She is financially independent. She worked from home due to the pandemic, though her job timings are generally standardized. All three members of her immediate household are earning members.

Meera's natal family was supportive of her working because they needed the money. Meera attributes much of her autonomy and security in her marital life to her financial independence and her job. She noted that she was *"much freer"* because she was a working lady, which gave her the liberty to go out, sometimes even without informing her family. She believes that if she had been a dependent housewife, the situation would have been *"something different,"* possibly leading her to die by suicide or walk out of the marriage.

When she was pregnant, she had to leave her job, but she returned to work after six months, and has continued teaching ever since.

3. History of Romantic relationships

a. Past Relationships

Meera was not in a romantic relationship before she got married.

b. Current relationship

Meera got married at age 27. It was an arranged marriage, and she knew her husband for *"barely two months"* before the wedding. Both families approved, and it was not an intercaste marriage.Meera's marital life was tough for several years after marriage. Her husband was brought up in a joint family, whose values and attitudes were much more conservative than her own background. One of the biggest troubles for Meera after marriage was the violent abuse from her mother-in-law. She faced harsh criticism, erratic, unpredictable and somewhat dangerous behaviour, and malingering from her mother-in-law which actively damaged and undermined her reputation and social standing in the neighborhood, and the home. Meera's husband

never admitted his mother needed proper psychiatric care, and this added to the troubles.

Meera's husband was very young when he lost his father. His mother, Meera's mother-in-law was unsettled from such a major trauma so early in her married life, and according to Meera, she was uneducated herself, without any way to step out of her marital home. Her mother-in-law never worked, so she was financially unstable, and had to depend on the generosity of the joint family to bring up her children. Meera's husband also had a sister, who died by suicide after she graduated college. According to Meera, she only came to know about this incident after her marriage. Her late sister-in-law had a romantic affair that led to some ugly fights within the family, ultimately leading her to end her own life. Meera suspected that all this also contributed to her mother in law becoming mentally more undone with time, but no one in her own family could understand her violent anger and erratic outbursts or behaviour. She did concede that her mother-in-law had a confrontational, belligerent attitude and was abusive by her nature, however, she did believe she understood where her mother-in-law's troubles began.
Meera's mother-in-law's behaviour grew increasingly erratic. Meera came to know from her neighbors that her mother-in-law had started selling household things like utensils to ragpickers (*kabadiwallahs*) , and even valuables like gold jewellery to "*random strangers*". Meera was increasingly convinced that her mother-in-law could not be left home alone without supervision, with her adolescent daughter, whose school board examinations were drawing near. She started to think about shifting her mother-in-law to an old-age home where she will be supervised adequately. Her husband vehemently opposed the idea, despite refusing to care for his mother himself. At that time Meera reached out to her family and extended family, and through their mediation her mother-in-law was moved to an old age home near their Kolkata abode. There also Meera visited her everyday before returning home from work, hired an ayah (attendant) for her care, brought her food everyday, specially prepared dishes and gifts during festivities etc. From her end, Meera thinks she did everything to ensure her mother-in-law lived a comfortable life. However, her husband was still resentful of her because of this decision.

Meera and her husband have not always lived together; for 20 years, they "*never stayed together*". While this was mostly related to her husband's job, through many of these years Meera was the sole caregiver for her daughter as well as her unstable mother-in-law, apart from holding full time employment.

She and her husband have only been staying together again for the past two years, during the pandemic and as they both approach retirement. Meera noted that she has taken vacations with her partner very rarely. She has taken more vacations with her friends and colleagues, taking a vacation with them every year for the past 10 years. She feels "*out of the world*" when she is alone with her friends, and considers these vacations as solace and bliss.

Meera describes her current relationship with her partner as based *"on compromise"*. She makes the financial decisions in the marriage, and her husband now respects her ideas and decisions. Decisions in the house are generally made through consensus. She is not emotionally dependent on her husband and says that she currently does not *"expect anything from my relationship"*. Meera still feels she is the primary caregiver, stating that she spends time caring about her partner *"Always,"* while her partner does not take care of her equally.

Meera stated that she wishes she had the life she is spending now, 30 years back, because she *"had to sacrifice a lot to come to this stage"*. Her husband is a project manager with a Master's degree and is 50 years old. He is currently working. He is also a heart patient, as is Meera.

The sexual relationship in the marriage was affected by the constant conflict caused by the mother-in-law. Meera stated that she *"did not have this type of relationship only"* and that the constant conflict resulted in her husband *"never had a chance to look after ourselves"*. Her husband never pressured her for sex. Meera mentioned that her husband is *"broad minded"* and never had issues with her speaking to other men in the workplace or socializing, and that this trait was one of the reasons she *"did not come out of the marriage"*.

4. Abuse History

a. Abuse in natal family

Meera reported that there was *"never"* any conflict in her natal family. She emphasized that she never heard her father or mother using abusive language or any vulgar slang.

b. Abuse in romantic and marital/cohabiting relationships

Meera did **not** experience physical violence, such as hitting, pushing, or shoving, or any sexual violence.

Abuse from the Husband (Verbal/Emotional): Meera's husband shouted at her "**Many a time**", starting on the "**very first day of my marriage**". He would shout "**indecent words**", which Meera was not used to, having "**never heard**" such language from her own parents. She found this initial experience "**very traumatizing**".

Meera noted that her husband tried to turn her against her family "**Many times from the very beginning,**" primarily due to the "**false allegation my mother-in-law used to bring**". However, Meera stated that her husband "**never said or done something to humiliate her in public**".

Abuse from the Mother-in-Law (Psychological and Verbal): Meera reported that conflicts were faced "**till my mother-in-law was alive**", and this was "**the only reason**" for the conflicts. Meera stated that her MIL was psychologically unwell, illiterate, never received treatment, and was very abusive. Meera’s husband never really accepted his mother’s need for psychiatric care, and despite not staying together with Meera, tasked her with his mother’s care. The mother-in-law used to "**go out and tell lies to our neighbors and all**", causing misconceptions about Meera. The mother-in-law's actions caused social damage that "**could not be healed**" in the locality.

She was abusive and used "**abusive languages**". **Physical Threats and Safety Concerns:** Meera "**never experienced physical violence**". However, she did feel "**physically threatened**" by her mother-in-law. When her husband was outstation, she had to keep watch on her mother-in-law, who would stay awake all night and throw tantrums. Meera had a feeling that "**anytime she can do anything to me**". She recalled waking up to find her mother-in-law "**standing beside my bed staring at me**". The door to her bedroom had to be kept open constantly, which enhanced her sense of vulnerability.

Later, the mother-in-law's mental health worsened. . Because she became "**very risky to keep**" at home, Meera made the difficult decision to place her in an old age home. This also became a sore point in their marriage because her husband was vehemently opposed to this idea, and wanted Meera to continue taking care of her as she was doing for many years. He himself refused to take his mother with him, and let her stay with him where he worked. Meera involved her extended family and through their mediation her decision finally prevailed. However, her husband bore a lasting grudge against her because of this decision, and Meera thought her husband

continues to feel *"very dissatisfied"* with the decision to send his mother to an eldercare facility.

Sexual Relationship: Meera reported that she "**did not have this type of relationship only**" due to the constant conflict in the house, which meant her husband "**did not get a chance only**" to look after their intimate relationship. She confirmed there was no instance of her husband pressuring her for sex, or him being unduly jealous of her time spent with friends outside of her home.

Coping and Resolution: Meera's primary response to the abuse and conflict was avoidance. When her husband shouted, she initially "**kept quiet**". Later, when arguments "**crossed the limit,**" she would reply, which often escalated the situation. Both she and her daughter have experienced bouts of humiliated rage when they have smashed household things. However, avoidance and suppressing the memory of these events was more often her response, especially early on in the marriage. She chose to "**stop talking**" and "**avoid everything**" concerning the in-law conflict.

Meera denied experiencing psychological symptoms, but attributed that to her strength of character and her mother's strength in her own upbringing. She never sought any legal relief but spoke to her husband's family about the abusive nature of her mother-in-law and the insults she was facing. While she never confessed the difficulties of her marriage entirely to her sisters, they could guess, and she said maybe *"they knew 50% of what was going on."* However, they were steadfast in offering their support through her life.

Throughout, concern for her daughter is a major theme in Meera's life story. As described above, she agonized over her inability to devote adequate attention to her daughter's upbringing because of her preoccupation with her mother-in-law's abuse and caregiving. This peaked when the conflict with her husband was unfolding, about rehoming her mother-in-law. This collided with the time that her daughter's school board examinations for grade 12 were drawing near, causing her further worry, since this would be a major career milestone for her daughter. Her daughter also steadfastly supported her during the difficult phase of She said that it was her daughter who stood by her at this time, and advised her to move on with her own life, despite her guilt and emotional trauma, and not think about the family. Meera acknowledged that she was "**on the verge of leaving him [her husband]** " when he was actively resisting her efforts to rehome her mother-in-law to the eldercare home, and thought about *"taking her daughter out"* of the unpredictable and potentially dangerous situation her mother-in-law's erratic admittance of random strangers in

the house was creating, but eventually chose not to, as her decision prevailed. She decided to "**handle the situation very... tactfully**". She emphasized that she tackled the situation using her brain and that she was able to do this because she was "**working**" and financially secure. To maintain the family's current healthy relationship, Meera believes it is necessary to "**forget**" the past, noting she tries to forget everything she discusses about the trauma immediately after speaking about it.

4. Impact in Mental and Physical Health

The impact of Meera’s 30-year marriage on her physical and mental health was primarily driven by continuous conflict and abuse, largely stemming from her mother-in-law, which Meera referred to as a source of significant "**emotional strain and trauma**".

Mental and Emotional Health Impacts

Experiencing Trauma and Stress: Meera repeatedly stated that she had a "**mental trauma**", particularly during the period when her mother-in-law was alive. She noted that she was "**very much down traumatized**" and "**agonized**" while worrying about whether she would be able to bring up her child properly. This conflict and trauma had a "**big impact on our relationship**" with her husband.

Meera found the abusive behavior, including her husband shouting "**indecent words**" starting from the "**very first day of my marriage**", to be "**very traumatizing**". She had "**never heard**" such language used by her own parents and could not believe that a mother or a husband could say these to a daughter-in-law or a wife. Some of the vocabulary that she termed as “slang” would be undecipherable for her, having never heard them before, and she also struggled to understand why she was being abused this way. Her later realization was that such vocabulary was regularly used in her marital home, but she hated it nonetheless.

Mother-in-Law's Mental Health: Meera noted that the primary source of conflict was her mother-in-law, who was psychologically unwell and "**very abusive**". Meera tried to facilitate the process of her mother-in-law’s psychiatric care early on in her marriage; She made an appointment with a psychiatrist, and asked her husband to accompany her mother-in-law to the appointment. She also arranged for all prescribed medications to be given timely to her mother-in-law. She noted it was too little too late by that time. The medications reduced violence from her mother-in-law

but they did not curb her abusive language and harshness, which had become ingrained in her.

Fear and Safety Concerns: While Meera "**never experienced physical violence**", she did **feel physically threatened** by her mother-in-law. When her husband was outstation, she had to care for her mother-in-law and reported that she "**never had a sound sleep**". She felt constantly vigilant, thinking that "**anytime she can do anything to me**", *particularly because she was forced to sleep with the bedroom door open.* Meera recalled one incident where she woke up in the middle of the night and saw her mother-in-law "**standing beside my bed, staring at me**", which frightened her considerably.

Meera noted that she "**should have**" felt afraid of her husband but believes she was able to maintain security because she was a strong person and "**had my own job**" and was "**financially sound**".

Coping Mechanisms and Resilience: Meera tackled the difficult situation "**tactfully**" and made a calculated decision to stay, believing that walking out of the marriage "**is not a solution**". Her approach involved using her brain and avoidance; she would "**stop talking**" and "**avoid everything**" related to the conflict.

Although she stated that she "**never**" had depressive thoughts or suicidal thoughts, she acknowledged that had she been a housewife, she "**would have thought of committing suicide**" or "**would have done something**".

Post-Trauma Resolution: Meera believes that for the sake of her "**mental health and for the family's health**", she must "**forget**" the past. She stated that the "**mental trauma has reduced significantly**" after her mother-in-law passed away, and she now consciously forgets everything she discusses about the trauma the moment she is done speaking about it. However, some of her statements also show that she now harbors a fundamental distrust in romantic relationships, as well as a generally heightened state of alertness. She advises younger girls, however, including the interviewer, to be always alert and vigilant, saying even if they marry someone they have been in a relationship with and know well, "it is all a gamble". She says "what you see now may not be who he is going to be after marriage" because men change after marriage.
Her daughter's relationship with her husband mended over time, and his relationship with her family was now *"not great but okayish"*.

Physical Health Impacts

Manifestation of Trauma: Meera believes that the trauma she suffered "**manifested itself**" in physical health conditions. She stated that she suffered "**Mental trauma**" and is currently a "**heart patient**". She noted that the year her mother-in-law passed away, she "**succumbed to my health issue**".

Impact on Daughter's Health and relationships : The abusive home environment directly affected Meera's daughter. As her mother-in-law's behaviour started getting more erratic, her relationship with Meera's daughter was also strained and uneasy. She describes her mother in law as a *"very antsy"* grandmother, who tried to take care of her granddaughter but simply couldn't. Meera reported that because of her mother-in-law's "**abusive belief,**" her daughter had to "**suffer**". The daughter often became **ill** and had to be "**hospitalized many a time during her childhood**". She also noted that her daughter's relationship with her own father was also somewhat strained because of her husband's indifferent ambivalence towards her own suffering.

5. Coping mechanism and Future outlook

Meera employed several coping mechanisms throughout her marriage. Most importantly, she credits her ability to survive and thrive due to her **financial independence** and being a working woman. She also often used **avoidance** as a strategy, trying to avoid conflict and *"stop talking"* to them during disputes. She actively attempts to **forget** the traumatic past for the sake of her mental health and family peace, stating that she will forget everything she spoke about once the conversation is over. She also advises others to be *"very alert"* and *"tackle the situation"* using their brain.

Meera's **social support network** was crucial; her sisters were very supportive. Her daughter encouraged her to reconnect with her friends, and Meera was able to broaden her social life by reconnecting with childhood friends and colleagues via Facebook, with her daughter's help. She maintains a "*very broad social network*" and feels happy and "*out of the world"* when she takes time for herself, such as traveling alone with friends/colleagues yearly.

Regarding her future outlook, Meera believes that her current quality of life is good. She is currently experiencing peace in her family; she noted that her husband, daughter, and extended family now have a healthy relationship. Meera believes that

"Every relation should be given time". She feels that her approach has ensured she did not develop the severe mental health issues she *"should have had"* given her circumstances. Meera relies on her innate resilience and strength of character to have borne through it all.

<u>File S6: Case Study: Maya</u>

Maya (59)

1. **Demographics and Family Background**

Maya is 59 years old and identifies as female. She currently lives in Kolkata. Maya has a strong educational background, having completed a BSc, BEd, TTC, and a special class in English. She is financially independent.

Maya is married but currently separated from her husband, a separation that began six or seven years ago, following a marriage that lasted approximately 15 to 17 years. She is not divorced. She has two sons, aged 33 (the elder) and 21 (the younger). Maya lives with her two sons, making up her immediate family of three. All three are earning members in the household, with Maya's take-home salary being 35,000 to 36,000 per month.

Growing up, Maya lived alone with her parents and brother until Class 7, after which they moved to Kolkata and lived in a joint family that included 31 family members. Her natal family was professional; her father was (unclear profession), and her mother was a chartered accountant officer, additional chief of DVC, and a geography honors graduate who stayed home. Maya has one sibling, a brother, who is a doctor. Maya reported no feeling of discrimination growing up, noting that girls in her household were respected and given more preference than boys, at the same time also recognizing her brother's studies mattered more because of his medical profession. As a child, she was heavily pampered by her aunts who would say, "*when she goes to her in laws place she will have to do everything. So we will not allow her to do anything at all*" Her mother held the most decision-making power and controlled the finances in the joint family. Maya's father was described as kind, quiet, and "*a lot scared*" of her mother. The family environment was characterized by little to no conflict beyond the usual amountHowever, Maya's mother enforced a very strict and disciplinarian upbringing on her, and she did not have much autonomy or mobility growing up.

Maya maintains very good, friendly relationships with her marital family (in-laws), even though her husband has no contact with them. Her mother-in-law, who recently passed away, was described as an "*excellent lady*" and was more of a friend

than an in-law to Maya. Maya also noted that her own mother and mother-in-law were very good friends.

2. Career

Maya is an assistant teacher (now called "teacher") by profession. She has 38 years of work experience. She describes her experience as "lovely" and notes that being a teacher was her childhood ambition. Her job timings are short, averaging 4 to 4.5 hours daily. She supplements her income by taking tuitions in English, which can add between four to six hours of work per day.

Both her natal family and her in-laws were supportive of her decision to work and become a teacher. Maya manages her family and work life by working hard. Currently, she is primarily responsible for the housework, which she notes is *"all day work"* and *"very tiring,"* especially since she took over cooking after her son was diagnosed with COVID-19 and Hepatitis A. She handles all financial decisions, though she now discusses them with her sons.

3. History of Romantic Relationships

a. Past

Before her marriage, Maya had a *"liking for a boy"* in the tenth grade, but due to strict parental control, she was *"not allowed"* to pursue a relationship or go out. Her lack of autonomy was enforced by her mother.

Maya entered her first relationship (her marriage) around age 20-21. It was a love marriage. The relationship lasted 15 years, or possibly 17 years. The marriage ended due to her husband's excessive drinking, which led to abuse, philandering and financial neglect on his part. Maya's husband, a software engineer/computer consultant, initially worked in an office but later left to pursue private work, which marked the beginning of their financial struggles. Maya's in-laws did not approve of the marriage initially, but later she developed a very good relationship with them, especially her mother-in-law.

b. Current Relationships

Maya is currently separated from her husband and stated she has had no other romantic relationships since. She still has not recovered from the separation,

signaling an ending of the marriage. She is not in touch with her husband, except for rare instances when he calls if he is in need.

4. **Abuse Histories**

a. Abuse in natal family

Maya reports **no history of abuse** in her natal family. She stated there was never "too much" conflict. She was highly pampered by her aunts and never had to perform chores. She felt respected and supported.

b. Abuse in marital/cohabiting relationships both by partners and their family

Maya experienced physical, emotional, verbal, and financial abuse, primarily linked to her husband's heavy drinking.

- **Verbal and Emotional Abuse:** Conflicts involving "**abusive languages**" were a daily occurrence. When drunk, the husband would "**talk rubbish**". He would also turn Maya's cousins out of the house after abusing them if they tried to interfere in his drinking habits. He did accuse Maya of **cheating** "**once or twice**".He never forced sex on her. Their decision to have the first son was mutual and the conception of the second son was unplanned. **Physical Violence and Threats:** Maya "**experienced physical violence**". She reported being **pushed or slapped**. Her husband also **beat their eldest son** violently when he did not beat her. .Her husband broke items in the house "**Many a time,**" including her cousin's gifts. She recalled him breaking glasses and plates.
- **Coping and Fear:** Maya generally responded to conflict by remaining passive; she "**never used to shout back a lot**" and would "**keep quiet**" to avoid escalating the situation, stating that she "**couldn't reply back**". She "**always used to feel scared**" when her husband would come home "**drunk, over-drunk**". She also worried about her "**Safety**" and the safety of her children.
- **Guilt and Responsibility:** While she did not initially think the abuse was her fault, she later acknowledged that "**you can't say it was always his fault. It was both at times, of course**".
- **Amnesia Regarding Abuse Details:** Maya repeatedly struggled to recall specific details about the abuse, stating she **doesn't remember** the first time he raised his hand, the first time he shouted, or the specific things he told her when he changed as a person. She noted that after so many years of marriage,

she wouldn't remember and "**I don’t remember all those things**". She also noted her husband "**never remembered anything, that was his plus point**".

Maya stated that conflicts were an everyday occurrence. She never shouted back, choosing instead to keep quiet. When things got unbearable, she remembers asking her husband to leave the house a few times, but that did nothing to stop the relentless drunken abuse, and the general financial neglect. She did report the abuse to her supportive mother-in-law, who tried to intervene unsuccessfully. Maya stated that it was not always his fault, acknowledging that "It was both at times" and noting that her inability to reply back was her *"only weak point"*.

Maya’s separation from her husband was prompted by her elder son, who feared the younger son would pick up his father's abusive language and behavior. The elder son currently acts as a father figure to the younger son (parentification).

5. **Impact on Physical and Mental Health**

The impact of Maya’s 17-year marriage, particularly the last six to seven years characterized by her husband’s heavy drinking and abusive behavior, resulted in emotional strain, fear, and the development of a chronic physical health condition.

Mental and Emotional Health Impacts

Maya reported significant emotional distress, daily fear, and lasting mental consequences, though she noted an absence of depressive or suicidal thoughts.

- **Persistent Fear and Anxiety:** Maya constantly felt scared when her husband would come home "**drunk, over-drunk**". She also worried about her "**Safety**" and the safety of her children.
- **Traumatic Nightmares and Avoidance:** While her husband was still living at home, Maya did have **upsetting dreams and nightmares** related to the situation, specifically if he "**came drunk**". However, after six years of separation, these nightmares are now "**okay**". Maya stated that she "**always used to feel scared**".To cope with the trauma, Maya admitted, "**I don't want to think up those things**". She practices chanting and meditation to "**forget all this stuff**".
- **Stress and Despair:** Maya stated that she is "**often stressed**" due to having to pay a large amount for her younger son's studies, worrying about how much

she could help and "**From where will I get the money?**". When stressed, she felt like she had "**no where to go, No one to tell**".

- **Amnesia and Minimization:** Maya struggled to recall specific details of the verbal and physical abuse. She noted that her husband "**never remembered anything, that was his plus point**". Maya minimized the severity of her husband's actions, stating he "**wasn't at all harmful, no**" and that the conflict only occurred "**when he drank**". Maya stated that when he was normal, he was a "**pretty polite person**" and "**never did anything of the sort**". She stated he never drank in front of her family but contradicted herself when she admitted him abusing her cousins and throwing them out of the house. While she described their conflicts as an everyday affair, she also conceded that the physical violence as "**very rare**" and "**not that common**"
- **Feelings of Disconnection and Negative Self-Perception:** Maya reported experiencing transient dissociative episodes, stating she feels "**Every time, every time**" disconnected, describing herself as "**Badly shaped**". She also still feels like a "**failure**" when she reflects on her ambition to study more. She has experienced feelings of **worthlessness**. She also sees the "**negative side of any good thing**" because she has "**suffered**".
- **Difficulty Recovering and Coping Strategies:** Maya stated that she "**still hasn't recovered**" from the ending of the relationship, which occurred six or seven years ago. When upset, she finds it "**not that easy at times**" to calm herself down. She loves reading. Her primary coping strategy when upset is to go to the **garden** (indoor plants or outdoor garden) because she "**cannot concentrate on reading" at that point**.
- **Self-Blame:** While initially denying that the abuse was her fault, Maya later stated that "**you can’t say it was always his fault. It was both at times, of course**". She also acknowledged that she was unable to reply back or fight back during conflicts.

Physical Health Impacts

Maya has experienced the onset of a chronic health condition linked to her marital period:

- **Chronic Condition:** When asked about her physical health since her marriage, Maya stated that "**During that time I had diabetes, so that deteriorates my health**". Her aggravated diabetes was one reason why Maya felt she had to take extra precautions through the pandemic years.

- **Physical Manifestations of Stress (Absence):** Unlike others, Maya reported that she has "**None, no**" pains or aches in her body when stressed. She also stated that she has not had a headache.

Carelessness about Safety: Maya exhibited careless behavior concerning her own safety, admitting that she is often unmindful, "**crossed the road without looking**".

6. **Coping mechanism and Future outlook**

Maya utilizes several coping mechanisms to manage stress and feelings:

- **Reading and Gardening:** She loves reading and gardening, which are her primary ways of spending time with herself. When she is upset, she goes to her garden (or indoor plants at night) because she cannot concentrate on reading.
- **Working:** Maya derives enjoyment from working and focuses on teaching, which helps her get up every morning.
- **Social Connection:** She maintains close ties, noting that "*joint family is fun*" and regularly meets her friends and cousins.
- **Avoidance and Minimization:** Maya deliberately avoids thinking about her past relationship issues ("*I don't want to think up those things*"). She says she tries to take things lightly and control her anger by remaining quiet.
- **Altruism:** Maya works with NGOs (unofficially), collecting money with friends to feed stray animals/people, and distributing clothes and school supplies to villages.
- **Spiritual Practice:** She does "*a little bit of chanting*" and meditation "*to forget all this stuff*".

Maya's future outlook is characterized by a mix of aspiration and fatalism: she envisions herself having a beautiful house with a garden in front, but immediately states, *"but that won't happen"*. She states that she is happy and feels she has control over her own life.

File S7: Case Study: Rhea

Rhea (59)

Demographics and Family Background

Rhea is a 59-year-old female who identifies as a school teacher. She holds a Master's degree and has completed a teacher's training course. She currently lives in Kolkata.

Natal Family Background: Rhea grew up in Jamshedpur with her parents and sisters (all siblings were female). She felt very secure, and her relationship with her parents was "*wonderful*". Her father, the only earning member initially, encouraged his daughters to create their own identity. Decision-making power was shared, with her mother mostly taking decisions after consulting with her father. Her father controlled the finances but was liberal and gave her mother freedom. Rhea reports no major conflicts growing up and never felt burdened with responsibilities from a young age.

Marital Family Background: Rhea has been married for 36 years and had to move out of her hometown post marriage. She has two sons and a granddaughter. Her immediate family now consists of seven people, including her granddaughter. The household currently includes Rhea, her husband (a chartered accountant, age 68/69), her sons, her daughter-in-law, her grandchild, and a bedridden aunt-in-law. Her husband, herself, her son, and her daughter-in-law are the earning members, with a rough estimated monthly family income of around 3.5 to 4 lakhs. Rhea's marital family belongs to a *"very big family"* and is *"economically very stable"*. Her in-laws live with them.

Family Psychological History: Rhea’s mother suffered from depression, later in life, but recovered with medication.However, an aunt-in-law in her marital family is "*a little hysteric*" and suffers from epilepsy. Her elder daughter-in-law experienced mood swing problems and a little depression when she first married, but is presently fine.

1. **Career**

Rhea's initial ambition was to become a nurse, inspired by Florence Nightingale, but her family objected to this profession as it was viewed as low-class. She subsequently pursued teaching. She began her job the day she completed her graduation exam and started her Master's. Before marriage, she was a known teacher locally and was also a Hindustani classical singer.

After her arranged marriage at age 23, she was told not to move out and work due to her conservative marital family. Her career stopped, and she was a *"total housewife"* for a period of years. She took the decision to return to work when her children were old enough. She successfully secured a job unexpectedly the day after she decided to seek employment.

Initially, her return to work was not accepted by anyone, including her husband. Rhea considers the ability to work her achievement, as it is crucial for her own independence and identity. Currently, she is a working school teacher with about 27–28 years of work experience. Her scheduled job timings are usually from quarter to eight to three for offline work, but online work *"doesn't have any limit"*. The family now generally accepts that women should work for their own independence, and most daughter-in-laws who wish to work are permitted to do so.

2. History of Romantic relationships

a. Past Relationships

Rhea did not have any romantic relationships before her marriage.

b. Current relationship

Rhea's marriage was arranged. She did not know her husband beforehand and first saw him on their wedding day during the *jaimala* ceremony. She was 23 when she got married.

Initial Phase: In the beginning, she lacked autonomy and freedom in the marriage. Neither partner met the other's expectations, and "*little things did annoy*" both, though they never shared these feelings. Physical intimacy was a "*part and parcel of life,*" not driven by love. Her husband did not support her during the initial stages of motherhood, especially when she was pregnant or alone with their children while he was away on tour. She notes that this lack of support affected their children psychologically.

Current Phase: After 36 years, Rhea and her husband have become friends and are interdependent. Her husband realizes what happened in the past and "*takes a lot of care*" of her now. He now tries to respect her ideas and decisions. They have worked on their relationship, and she feels they have both changed themselves. After arguments, the husband typically takes the initiative to talk and "*sort it out*". They take family vacations together, which she enjoys.

3. **Abuse History**

a. Abuse in natal family

Rhea's experience in her natal family was generally positive; she reported feeling "*very, very secure* " and did not recall any conflict. Rhea was married at the age of 23. However, her upbringing definitely reflects restriction of autonomy and agency, and the values inculcated were in line with patriarchal ideologies. Rhea's initial ambition was to become a nurse, inspired by Florence Nightingale, but her family objected to this profession as it was viewed as low-class. She subsequently pursued teaching. She began her job the day she completed her graduation exam and started her Master's. Before marriage, she was a known teacher locally and was also a Hindustani classical singer.

b. Abuse in romantic and marital/cohabiting relationships

- **Initial Difficulties and Conservative Environment:** Rhea reported that in the beginning, the relationship was difficult because she "**did not know him, he did not know me**". She felt that neither was "**upto**" the other's expectation. She married into a "**very conservative family**" where she was initially told "**I should not move out and work**". She spent years as a "**total housewife**".
- **Lack of Autonomy:** Initially, Rhea felt she had "**no autonomy at all**" and lacked freedom. The family environment led to her being "**totally disconnected**" from her friends and relatives. She had to hide her communication, stating that she would "**create certain situations, tell them something else and go to the post office and get letters**" to keep in contact with family outside the city.
- **Decision Making:** Early in the marriage, the "**man of the house was the decision maker**". Decisions regarding her children were "**taken by husband's family**". Rhea took on financial responsibility "**voluntarily**" to help her natal family after her father's death.

- **Emotional/Verbal Abuse and Control:** Rhea married into a conservative family where she was restricted from working. Early in the marriage, she felt compelled to adjust and "*accept whatever they say*" to maintain peace, leading her to lose her "*peace of mind*". The conflicts that arose caused hurt, and she coped by choosing to "*keep quiet and never protest*". She was worried that if her mother protested against her in-laws, Rhea herself would suffer.

She reports that she never felt afraid of her husband or physically threatened. However, she acknowledges that his lack of support and decisions that went against her and her children's well-being were a *"kind of mental torture"*. Initially, she normalized this treatment, believing these were "*natural things that happen to every woman".*

Her husband has shouted at her, though rarely, typically concerning their children. She sometimes felt she shared the fault or that circumstances caused the conflict. She was humiliated by her husband in social or public settings both early in the marriage and currently, although she objects now. He previously criticized her intelligence and treated her as an inferior, though this has stopped.

Abuse by In-laws: Rhea maintained a formal relationship with her in-laws and did not feel they were very caring. Her in-laws often negatively commented on her mother after her father's death, particularly regarding her mother being a widow (e.g., criticizing her for having cake or wearing a colored blouse), which Rhea found highly distressing and described as a "*nightmare*". When her mother was widowed right after her marriage, her family also depended on her financially- while she herself was unemployed and a housewife. This initial support then had to come from her husband's earnings or his family- which further complicated the situation and invited snide taunts to Rhea from her marital relatives, about how they worried about the new financial strain on them because of her mother and sisters' financial responsibility. Even when her mother came to visit her in her marital homes, she was served frugal meals, and treated with barely disguised animosity, worsening the humiliation. Her mother never protested, fearing Rhea would suffer. The way her mother was treated by her in-laws gave Rhea a lot of anguish, but she could not find enough courage to protest.

Sexual Coercion: Rhea experienced instances where her husband pressured her for sex when she was not in the mood. She felt she had *"no options"* but to comply. She felt he used force. When she disagreed, she felt she was *"never heard of"*. This affected

her long-term, causing hurt, and she was *"not happy"*. She admitted that this coercion happened *"not very frequently but yes, it happened"*.

4. **Impact in Mental and Physical Health**

Rhea's sustained coping mechanism of remaining silent and refusing to protest against marital conflicts resulted in *"another depression"*. She experienced despair and hurt due to the conflicts and being coerced into sex. She felt emotionally subdued and had *"no space to express emotions,"* instead *"gulp[ing] it down"*. She now realizes that the verbal and emotional treatment she received was a form of mental torture. Presently, she wakes up with stress about her schedule, constantly thinking about *"what to do now, what's next,"* and is always busy.

Coping mechanism and Future outlook

Coping Mechanisms: In the initial years of marriage, Rhea's main mechanism to resolve conflicts and maintain peace was to "*keep quiet and never protest*". She would gulp down her feelings and emotions as there was *"no outlet".* She did not discuss these issues with anyone outside her marital family due to the perceived social taboo. She tried to rationalize the treatment she received, initially viewing the emotional torture as *"natural things"* that happen in life. She also sometimes blamed herself for the disagreements.

Rhea could not consider separation or divorce as a viable option, because initially her family was also dependent on her husband's income financially. She also reasoned that in the *"middle-class"* social strata that she belonged to, a divorce was only an option for people who had male relatives like brothers and fathers to support them. She cites *"the lack of manpower"* as one of the principal reasons for sustenance of her marriage as opposed to a separation.

Rhea voluntarily took on the responsibility of earning to help her parental family. She eventually took the decision to work for her own identity and independence after 16 years of marriage, and since then has repaired a lot of damage in her marriage. She uses her granddaughter as a source of relaxation, playing and singing with her. She also meets with friends once a month, though less often due to the pandemic. They sometimes go on family vacations as well.

5. **Future Outlook:** Rhea believes that time, maturity, adjustments, and sorting things out with a *"cool temperament"* have made her current situation much

better. She feels that both she and her husband have changed themselves for the better. She strongly advocates that every girl should work for their own independence and identity. She feels that her relationship with her husband has now evolved into one of friendship and interdependence.

File S8: The SPECTRA supplement

File S8A: The SPECTRA Model: Conceptual Framework and Theoretical Positioning

S8.1 Overview and Conceptual Rationale

The SPECTRA (Socially and Psychologically Embedded Continuous Trauma in Relational Architectures) model is proposed as a feminist, culturally grounded conceptual framework for understanding domestic violence (DV) in India through a psychological lens. The model responds to limitations in existing approaches that conceptualize violence as discrete events or rely primarily on diagnostic frameworks.

SPECTRA integrates insights from participants' narratives with gaps identified in the literature to conceptualize DV as a **continuous, relational, and developmentally embedded process**. While informed in part by constructs from complex trauma literature, the model does not adopt a diagnostic structure. Instead, it provides a **theoretical scaffold** for systematically examining how violence is experienced, internalized, and negotiated across the life course.

The framework emphasizes that DV outcomes are shaped through the interaction of:

- Developmental experiences
- Relational dynamics
- Socio-cultural norms and expectations
- Structural constraints

These interacting levels produce patterned trajectories that cannot be adequately understood through isolated categories such as physical or sexual violence alone.

S8.2 Developmental and Relational Foundations of SPECTRA

SPECTRA conceptualizes violence as emerging from a **cumulative and layered process** beginning early in life and unfolding across relational contexts.

S8.2.1 Natal Family Contexts and Gender Socialization

The framework foregrounds early experiences within natal families as foundational. These include:

- Direct exposure to violence, neglect, or deprivation
- Witnessing violence against female family members

- Differential treatment based on gender (e.g., son preference)
- Restriction of autonomy, mobility, and aspiration

Such experiences contribute to the early internalization of:

- Gendered hierarchies
- Norms of obedience and adjustment
- Expectations of self-sacrifice

These processes shape emerging models of self, relationships, and acceptable forms of behavior, often normalizing constraint within intimate relationships.

S8.2.2 Early Romantic Relationships and Pre-Marital Vulnerability

The framework recognizes that vulnerability to relational harm may be present prior to marriage. Early romantic relationships are often shaped by:

- Internalized norms discouraging disclosure of relational difficulties
- Cultural constraints around dating and partner choice
- Secrecy and limited social support

These conditions may allow abusive dynamics to develop without recognition or intervention, reinforcing dysfunctional relational expectations.

S8.2.3 Marital and Adult Relational Contexts

In adulthood, particularly within marital and extended family systems, these earlier patterns are intensified. The framework accounts for:

- Multiple forms of violence (physical, sexual, psychological, economic)
- Coercive control, surveillance, and restriction of autonomy
- Pressures associated with virilocal residence and extended family dynamics
- Devaluation of domestic and caregiving labor

These conditions often produce sustained relational constraint, within which women may have limited opportunities to seek support or exit.

S8.3 Psychological and Somatic Adaptations

SPECTRA situates psychological and physiological alterations within the context of **prolonged and relationally embedded stress**. Rather than treating these as isolated

symptoms, the framework conceptualizes them as **adaptive responses** emerging from cumulative exposure to constraint, unpredictability, and devaluation.

These adaptations may include:

- Affective dysregulation and emotional numbing
- Somatic expressions of distress
- Disruptions in identity and self-concept
- Alterations in relationships and trust
- Changes in meaning-making and belief systems

While these patterns show convergence with constructs described in complex trauma literature, their manifestation is shaped by culturally specific norms and relational expectations.

S8.4 Negotiation, Agency, and Adaptive Repair

The framework emphasizes that survivors are not passive recipients of violence but engage in **ongoing processes of negotiation and adaptation**.

These may include:

- Strategic silence and compliance to manage risk
- Partial or negotiated forms of exit (e.g., separation)
- Boundary-setting and relational recalibration
- Re-engagement with work, social networks, and support systems

Agency is therefore understood as **constrained and contextually shaped**, rather than as unrestricted autonomy. Processes of recovery are often incremental and negotiated within structural and cultural limits.

S8.5 Conceptual Mechanism Summary

The SPECTRA model proposes that:

1. Gendered developmental conditioning precedes and shapes later vulnerability to relational harm
2. Violence becomes embedded within relational systems rather than occurring as isolated events
3. Psychological and somatic adaptations emerge cumulatively over time

4. Survivors negotiate and adapt within structural and cultural constraints
5. Trauma is sustained through relational architectures, not solely through individual perpetrators

S8.6 Positioning Relative to Existing Trauma Frameworks

SPECTRA is informed by, but conceptually distinct from, major trauma formulations, including:

- PTSD (DSM-5)
- PTSD and Complex PTSD (ICD-11)
- Disorders of Extreme Stress Not Otherwise Specified (DESNOS)
- Developmental Trauma Disorder (DTD)

These frameworks have advanced understanding of trauma-related psychological alterations, including dysregulation, identity disturbance, and relational disruption. However, they remain primarily:

- Diagnostic
- Event-based
- Centered on intrapsychic or interpersonal disturbance

S8.7 Key Points of Divergence

SPECTRA differs from these frameworks in three fundamental ways:

1. Non-diagnostic orientation

SPECTRA does not define a syndrome or symptom threshold. It conceptualizes trauma as a relational and developmental process rather than a disorder located within the individual.

2. Continuous rather than event-based conceptualization

Unlike frameworks requiring identifiable traumatic events, SPECTRA conceptualizes trauma as developmentally sedimented within everyday relational environments.

3. Structural and cultural embedding

SPECTRA foregrounds culturally specific relational systems—such as kinship structures, gender norms, and community regulation—as constitutive mechanisms of trauma. Psychological alterations are understood as patterned adaptations within these systems.

S8.8 Summary

SPECTRA extends existing trauma frameworks by integrating:

- Developmental embedding
- Relational captivity
- Psychological and somatic adaptations
- Negotiation and adaptive repair

within a unified conceptual model. It shifts the analytic focus from discrete events and diagnostic categories toward **continuous, relationally structured processes shaped by cultural and social contexts**.

8B : Supplementary tables S1 and S2

Table S1: SPECTRA domains and explanations (Extended)

Domain	Core Psychological Process	Mechanisms of Operation	Cultural Specificities in Indian Context	Narrative Indicators (Analytic Markers)	Implications Beyond Diagnosis
**1. Developmental Psychic Embedding**	Early embedding of gendered relational hierarchies through developmental socialization within family systems	Repeated exposure to gendered hierarchy and asymmetrical power<br><br>Internalization of obedience, adjustment, and relational roles<br><br>Early normalization of constraint through familial interactions and observation	Patrilineal and patrilocal family systems<br><br>Son preference and differential gender socialization<br><br>Norms surrounding the "good daughter" and "future daughter-in-law"	"That was normal"<br><br>"Girls have to adjust"<br><br>"We were not allowed to…"<br><br>Early restriction of mobility, decision-making, or expression	Reframes trauma as developmental conditioning rather than event-based injury
**2. Relational Captivity**	Entrapment within coercive, hierarchical, and unpredictable relational systems	Economic dependency and restricted mobility<br><br>Social isolation and disconnection from natal support systems<br><br>Surveillance and regulation of behavior within extended family structures	Joint and extended family systems<br><br>Collective family honor and reputational control<br><br>Intergenerational enforcement of patriarchal norms<br><br>Stigma surrounding divorce and separation	"I was not allowed…" / "I had to ask permission…"<br><br>"I couldn't go back to my family…"<br><br>"You never knew what would happen…"<br><br>"Things would change suddenly…"	Captivity mediated by relational architectures, not only individual perpetrator behavior

Domain	Core Psychological Process	Mechanisms of Operation	Cultural Specificities in Indian Context	Narrative Indicators (Analytic Markers)	Implications Beyond Diagnosis
		Control over sexuality, reproduction, and financial decision-making<br><br>Use of threat, unpredictability, and emotional manipulation to regulate behavior		"It was better to stay quiet…"	
**3. Psychological and Somatic Adaptations**	Survival-oriented adaptations to prolonged relational constraint leading to identity-level, affective, somatic, and meaning-system changes	Persistent affect dysregulation and emotional instability<br><br>Somatic expression of distress in the absence of clear medical pathology<br><br>Disruptions in consciousness, including dissociation and detachment<br><br>Internalization of self-blame, shame, and damaged self-concept<br><br>Relational withdrawal, mistrust, and altered expectations of others	Idioms of distress (e.g., "tension," "dard")<br><br>Stigma surrounding mental health and emotional expression<br><br>Cultural normalization of endurance and sacrifice<br><br>Moral framing of suffering and relational duty;	"I feel tired all the time… doctors find nothing wrong"<br><br>"I feel empty / I feel nothing"<br><br>"Maybe it was my fault"<br><br>"I am not the same person anymore"<br><br>"I don't trust anyone now"<br><br>"This is my fate / women have to tolerate"	Moves beyond symptom-based frameworks to capture identity restructuring, relational disruption, and culturally embedded meaning-making processes.

Domain	Core Psychological Process	Mechanisms of Operation	Cultural Specificities in Indian Context	Narrative Indicators (Analytic Markers)	Implications Beyond Diagnosis
		Restructuring of beliefs and meaning systems, including normalization of suffering			
**4. Negotiation and Adaptive Repair**	Constrained agency expressed through strategic negotiation, endurance, and incremental reconfiguration of relational roles and self-perception	Strategic silence, compliance, and endurance to manage risk<br><br>Negotiation within structural constraints rather than full exit<br><br>Partial or staged exit strategies (e.g., separation)<br><br>Relational recalibration and boundary-setting over time<br><br>Rebuilding agency through work, social networks, and selective autonomy	Reliance on natal family support; culturally embedded coping (e.g., meditation, religious practice); community mediation; negotiated autonomy rather than exit;	"If I spoke, things would get worse"<br><br>"Leaving was not an option"<br><br>"Separation felt safer than divorce"<br><br>"People will talk"<br><br>"My children stood by me"<br><br>"I started working again / I rebuilt my life slowly"	Rejects passive victim model; foregrounds adaptive intelligence and relational navigation

Table S2: Conceptual Comparison of Clinical Trauma Frameworks and SPECTRA

Construct	Primary Source / Citation	Core Definition	Trauma Framing	Primary Locus of Disturbance	Developmental Scope	agnostic Status	How It Differs from SPECTRA
**PTSD (DSM-5)**	American Psychiatric Association, 2013	Trauma- and stressor-related disorder requiring exposure to a qualifying traumatic event; four symptom clusters (intrusion, avoidance, negative cognition/mood, arousal)	Event-based; requires identifiable traumatic exposure	Intrapsychic symptom clusters	Any age following trauma	Formal DSM-5 diagnosis	SPECTRA rejects event-threshold model; conceptualizes trauma as continuous and relational rather than discrete and symptom-defined
**PTSD (ICD-11)**	Brewin et al., 2017	Narrower PTSD with 3 core clusters: re-experiencing, avoidance, persistent threat	Event-based; parsimonious diagnostic model	Fear-based trauma response	Any age following trauma	Formal ICD-11 diagnosis	SPECTRA moves beyond fear-response model to identity restructuring and relational captivity embedded in social systems
**Complex PTSD (ICD-11)**	Brewin et al., 2017; Cloitre, 2020	PTSD plus Disturbances in Self-Organization (affective dysregulation, negative self-concept, interpersonal disturbance)	Prolonged interpersonal trauma; still event-linked	Intrapsychic and interpersonal dysfunction	Typically chronic trauma contexts	Formal ICD-11 diagnosis	SPECTRA is non-diagnostic; embeds identity disturbance within culturally specific relational architectures rather than symptom criteria

Construct	Primary Source / Citation	Core Definition	Trauma Framing	Primary Locus of Disturbance	Developmental Scope	agnostic Status	How It Differs from SPECTRA
**Disorders of Extreme Stress Not Otherwise Specified (DESNOS)**	Luxenberg et al., 2001; van der Kolk et al., 2005	Disorders of Extreme Stress Not Otherwise Specified; broad disturbances in affect regulation, consciousness, self-perception, relations, somatization, meaning	Chronic interpersonal trauma; not restricted to narrow symptom triad	Multi-domain psychological disturbance	Often adult survivors of prolonged abuse	Proposed (DSM-IV field trial), not adopted	SPECTRA does not conceptualize trauma as disorder; foregrounds structural power and cultural kinship systems as constitutive mechanisms
**Developmental Trauma Disorder (DTD)**	Ford et al., 2013; van der Kolk et al., 2019	Chronic childhood interpersonal trauma leading to disorganization across affective, cognitive, somatic, and behavioral development	Chronic early caregiving trauma	Neurodevelopmental and regulatory disruption	Childhood and adolescence	Proposed diagnosis (not adopted in DSM-5)	SPECTRA includes developmental embedding but extends across lifespan and incorporates adult relational captivity and negotiation
**SPECTRA Model**	Present study	Socially and Psychologically Embedded Continuous Trauma in Relational Architectures; four domains: developmental embedding, relational captivity, psychological and somatic adaptations, negotiation and adaptive repair	Continuous, relational, developmentally sedimented trauma	Relational architectures + identity-level restructuring	Lifespan continuum	Non-diagnostic conceptual framework	Centers culturally specific kinship systems, structural inequality, and negotiated agency; reframes trauma as patterned relational process rather than clinical disorder

File S9: Supplementary Methods

S9.1 Literature Review Strategy

A structured, theory-oriented review of existing literature was conducted to identify conceptual gaps in psychological approaches to domestic violence (DV), with a particular focus on the Indian and broader South Asian context. This review was not designed as a systematic review of prevalence or intervention outcomes. Instead, it functioned as a conceptual synthesis aimed at situating the present study within existing theoretical and empirical work.

Sources included peer-reviewed journal articles, books, and policy reports accessed through databases such as PubMed, PsycINFO, and Google Scholar. The review focused on three interrelated domains:

1. **Psychological frameworks of DV and trauma**, including PTSD, complex trauma, and related constructs
2. **Socio-cultural analyses of gender, family, and kinship systems in India**
3. **Empirical research on women's lived experiences of DV in South Asian contexts**

The primary objective of this review was to identify limitations in dominant frameworks—particularly their emphasis on discrete events, diagnostic thresholds, or decontextualized symptom clusters—and to inform the development of a relational and developmental conceptual model.

S9.2 Epistemological and Analytical Orientation

This study is grounded in a **feminist, relational epistemological framework** that views knowledge as contextually situated, relationally produced, and shaped by structures of power. Rather than assuming neutrality, the analysis treats participants' narratives as meaning-making practices embedded within socio-cultural and relational contexts.

The analytic approach prioritizes:

- Lived experience over diagnostic categorization
- Relational dynamics over isolated individual behavior
- Culturally embedded meaning-making over universalized constructs

Narratives are understood not simply as reports of events, but as interpretive accounts through which participants construct identity, negotiate responsibility, and make sense of relational experiences across time.

Language was treated as analytically significant. Particular attention was paid to culturally embedded idioms, metaphors, and normative terms (e.g., "adjustment," duty, respectability), which function as vehicles of both constraint and meaning-making within participants' accounts.

The analytic process therefore involves interpretation as an integral component of knowledge production, while maintaining reflexive awareness of the researchers' role in shaping that interpretation.

S9.3 Narrative Interview Design

The interview schedule was designed to elicit extended narratives rather than short, categorical responses, consistent with narrative analytic principles (Bamberg, 2021). The goal was to generate data that captured experiences across time, relationships, and socio-cultural contexts, enabling multi-level analysis.

Interviews were organized around key domains, including:

- Experiences in natal families
- Intimate and marital relationships
- Work–life negotiations and gendered labor
- Psychological and emotional experiences across the lifespan
- Meaning-making processes

Instead of asking participants to simply report whether specific forms of violence occurred, we invited them to narrate experiences temporally and relationally.

(*What we tried to investigate: "What happened?"*)

Participants were encouraged to describe events in sequence, beginning with childhood and moving through romantic and marital relationships. Prompts such *as "Can you tell me about what growing up in your family was like?"* were designed to elicit developmental continuity rather than isolated incidents.

S9.3.1 Use of Probes and Narrative Elicitation

Follow-up probes within core questions of each domain asked participants about landmark incidents in their lives, happy or sad memories, exposure to abusive relational events or violent events. Probes were used flexibly to deepen narrative elaboration and to support analysis across multiple levels:

- **Temporal probes** (e.g., *"How did this change over time?"*)
  → to capture developmental continuity and sequencing

- **Relational probes** (e.g., *"How did others respond?" "What would happen if you protested?"*)
  → to examine relational dynamics and positioning
- **Meaning-making probes** (e.g., *"What did this mean to you?" "What does 'adjustment' mean in your context?")*
  → to access interpretive frameworks and self-understanding

In addition, some probes were informed by complex trauma literature (e.g., affective shifts, bodily experiences, relational changes), but were used as **sensitizing prompts rather than fixed categories**.

For example, participants were asked to describe:

- How everyday decisions were made within families
- What was considered "normal" or expected behavior
- How they understood their own responses (e.g., silence, resistance, adjustment)
- Their own positioning in relation to others

This approach enabled the generation of layered narrative data that could be analyzed simultaneously at thematic, relational, and socio-cultural levels.

S9.4 Linking Data Generation to Analysis

The design of the interview schedule was closely aligned with the analytic framework. Narrative elicitation strategies were intended to produce data that could be examined across three interconnected levels:

- **Thematic level**: patterns of experience and developmental trajectories
- **Positioning level**: constructions of self, agency, and relational roles
- **Socio-cultural level**: invocation of cultural norms, values, and discourses

By structuring interviews to elicit temporal sequences, relational contexts, and interpretive reflections, the study ensured that analytic categories were grounded in the form and content of participants' narratives rather than imposed post hoc.

S9.5 Participants and Sampling Strategy

S9.5.1 Sampling Approach

The study employed a combination of **convenience and snowball sampling** to recruit participants. Initial participants were approached through the researchers' extended professional and social networks, as well as through targeted outreach via

social media. Subsequent participants were identified through referrals, allowing access to individuals who may not have responded to formal recruitment calls but were willing to participate through trusted interpersonal channels.

This approach was selected for both **pragmatic and ethical reasons**. Given the sensitivity of discussing intimate relationships and experiences of violence, recruitment through relational networks facilitated trust, disclosure, and participant safety. At the same time, it enabled access to experiences that may remain hidden in more formal or institutional recruitment contexts.

S9.5.2 Inclusion Logic and Conceptual Rationale

Participants were required to have experienced at least one **significant intimate relationship**, defined as:

- a relationship lasting six months or longer, or
- a cohabiting relationship, with or without extended family involvement

Notably, **experience of violence was not used as an inclusion criterion**. This decision was conceptually motivated. The study aimed to examine how relational constraint, control, and gendered expectations operate within everyday life, including forms of harm that may not be labeled as "violence" by participants themselves.

This allowed the analysis to capture:

- normalized or routinized forms of coercion
- culturally sanctioned expectations (e.g., adjustment, duty)
- early-stage or ambiguous forms of relational harm

rather than only cases that meet explicit thresholds of recognized abuse.

S9.5.3 Participant Characteristics and Contextual Positioning

The sample consisted of seven urban, educated Indian women between the ages of 28 and 60. Participants represented diverse relational trajectories, including:

- arranged marriages
- self-initiated (love) marriages
- separation and divorce
- non-marital intimate relationships

Most participants had current or prior engagement in professional work. However, several described periods of restricted employment or enforced domestic roles

within marital or familial contexts. These shifts were analytically relevant, as they reflected intersections between gendered expectations, economic dependence, and relational control.

Geographically, the sample included participants from Kolkata (n=5), Haryana (n=1), and Hyderabad (n=1). While not intended to be regionally representative, this distribution allowed for examination of both shared and context-specific relational patterns across urban Indian settings.

S9.5.4 Sampling Limitations and Analytical Position

The sample is **small, urban, and socioeconomically specific**, and does not aim to represent the diversity of women's experiences across India. Rather, participants are treated as **analytically informative cases** that allow for in-depth examination of relational processes and meaning-making practices.

The goal of sampling in this study was therefore **theoretical and conceptual**, not statistical. The emphasis was on:

- depth of narrative
- richness of relational context
- variability in life trajectories

These cases function as sites through which broader relational and cultural mechanisms can be examined and theorized.

S9.6. Data Collection Procedures

S9.6.1 Interview Modality and Structure

Data were collected through **in-depth, semi-structured online interviews**. The online format was selected to ensure participant comfort, privacy, and logistical feasibility, particularly when discussing sensitive topics.

Each interview followed a flexible structure organized around core domains (see Section S3), while allowing participants to guide the flow of narration. Interviews typically progressed from early life experiences (e.g., natal family) to later relational contexts (e.g., marriage or partnerships), enabling the reconstruction of **developmental trajectories**.

S9.6.2 Narrative Elicitation in Practice

The interview process was designed to move beyond factual reporting toward **extended narrative construction**. Participants were encouraged to:

- describe sequences of events rather than isolated incidents
- reflect on changes over time
- situate experiences within relationships and family systems
- articulate their interpretations, doubts, and contradictions

For example, participants were invited to elaborate on:

- how decisions were made within the household
- how expectations of "adjustment" or duty were communicated and enforced
- how their own responses (e.g., silence, compliance, resistance) evolved over time

Probing was adaptive rather than scripted. Interviewers followed participants' narratives while selectively introducing prompts that deepened:

- temporal continuity ("What changed after that?")
- relational positioning ("What did you feel you were expected to do?")
- socio-cultural framing ("Was this considered normal?")

This approach allowed narratives to retain their coherence while generating analytically rich material across multiple levels.

S9.6.3 Trauma Sensitivity and Ethical Safeguards

Given the sensitive nature of the topic, interviews were conducted with explicit attention to **trauma-informed principles**.

Key safeguards included:

- detailed informed consent procedures prior to participation
- reiteration of voluntary participation and the right to skip questions or withdraw
- sensitivity to emotional distress during interviews
- immediate debriefing following the session
- provision of referral resources for psychological support

Interviewers received prior training in:

- qualitative interviewing and active listening
- trauma sensitivity
- psychological first aid

Where needed, referral pathways to trained mental health professionals were made available.

S9.6.4 Reflexive Documentation

Throughout the data collection process, researchers maintained:

- **field notes** documenting contextual observations, interactional dynamics, and affective tone
- **reflexive journals** capturing immediate impressions, emerging interpretations, and potential biases

These materials served both as contextual anchors for later analysis and as tools for maintaining reflexive awareness of the researchers' role in the co-construction of narratives.

S9.7 Coding Framework and Analytic Strategy

Coding was conducted using a multi-layered framework designed to capture different dimensions of narrative data. Codes were not mutually exclusive, and multiple codes could be applied to the same segment.

The coding system included:

- **Open codes (O)**: descriptive coding of events and experiences
- **Process codes (P)**: actions, sequences, and ongoing relational dynamics
- **Positional codes (Po)**: how participants positioned themselves and others
- **Socio-cultural codes (S)**: cultural norms, expectations, and structural influences
- **Thematic codes (T)**: higher-order conceptual groupings

Interactions between coding types (e.g., Po × S) were used to capture how identity, meaning-making, and socio-cultural structures co-occurred within narratives.

S9.8 Stepwise Analytic Process

Analysis proceeded through iterative and interconnected phases rather than as a strictly linear sequence.

S9.8.1 Data immersion and narrative structuring

Each transcript was first read holistically to understand the narrative arc, including:

- chronological sequencing
- key life events and transitions
- relational shifts
- explicit and implicit emotional trajectories

Short narrative summaries were developed for each participant to preserve coherence prior to coding. Analytic memos were written during this stage to document initial observations and emerging questions.

S9.8.2 Open coding and code generation

Three researchers independently conducted line-by-line open coding. Codes were generated inductively from participants' language and reflected both descriptive content and relational processes.

Coding decisions were documented through analytic memos, which specified:

- the rationale for code assignment
- whether a segment reflected experience, interpretation, or justification
- points of ambiguity or alternative readings

More than 80 initial codes were generated at this stage, capturing a wide range of experiential, relational, and cultural dimensions.

S9.8.3 Thematic consolidation and subtheme development

Codes were compared across cases to identify patterns of recurrence, co-occurrence, and developmental sequencing. Conceptually related codes were clustered into subthemes representing shared relational processes.

Analytic decisions at this stage were guided by multiple criteria:

- recurrence across participants
- coherence within narrative sequences
- developmental continuity
- explanatory relevance to relational processes

For example, codes relating to surveillance, mobility restriction, and decision-making control were clustered into subthemes reflecting restriction of agency and autonomy. Similarly, codes reflecting different forms of violence and control were grouped into higher-order relational patterns.

Subthemes were iteratively refined and, where necessary, reorganized to preserve both analytic clarity and narrative coherence.

S9.8.4 From subthemes to themes

Subthemes were further consolidated into broader thematic structures that captured distinct dimensions of participants' experiences. Certain subthemes were sufficiently complex to include nested subdomains.

Five higher-order thematic domains were initially generated:

- experiences in the natal family
- relational dynamics within marriage and extended family
- coercive and unpredictable relational contexts
- psychological and somatic adaptations
- response, negotiation, and repair

S9.9 Analytic Transition to SPECTRA Domains

A key analytic step involved reorganizing descriptive themes into conceptual domains that capture underlying relational and developmental processes.

This transition was not a direct or singular step but involved iterative comparison and abstraction. Themes were evaluated for conceptual overlap, underlying mechanisms, and explanatory scope.

In particular:

- themes relating to marital relational dynamics and captivity-like conditions were analytically merged into the domain of **relational captivity**, reflecting their shared emphasis on sustained constraint and entrapment
- experiences in the natal family were interpreted as reflecting early internalization of relational norms (**developmental embedding**)
- psychological and somatic patterns were conceptualized as adaptations to prolonged relational constraint
- patterns of response and resilience were interpreted as processes of **negotiation and adaptive repair**

This process resulted in four interrelated conceptual domains forming the SPECTRA framework.

S9.10 Abductive Theoretical Engagement

Following inductive theme development, the analysis engaged with theoretical frameworks—particularly from complex trauma literature—as sensitizing constructs.

These constructs were used to:

- identify points of convergence with established trauma frameworks
- examine where participants' narratives extended or modified existing concepts
- interpret culturally specific mechanisms not fully captured in existing models

For example, while certain psychological patterns aligned with constructs such as affect dysregulation or altered self-perception, the normalization of "adjustment" emerged as a culturally specific mechanism shaping both experience and interpretation.

The analytic process therefore involved continuous movement between empirical material and theoretical frameworks, resulting in a culturally grounded conceptual model.

S9.11. Analytic Traceability and Mapping

The transition from codes to subthemes, themes, and conceptual domains involved iterative clustering and abstraction. A detailed mapping of this analytic process is provided in **Appendix 10D (Tables S3A–S3D)**.

- Tables S3A–S3B illustrate the progression from codes to initial and refined subthemes and their organization into themes
- Tables S3C–S3D present the mapping of analytic codes and data fragments to subthemes and SPECTRA domains, including indicators of intermediate clustering

These tables provide an explicit audit trail of analytic decisions and demonstrate how the conceptual framework is grounded in narrative data.

S9.12 Thematic Map and Analytic Iteration

A preliminary thematic map (Figure S1) was developed during intermediate stages of analysis to organize emerging patterns across domains. This figure represents an analytic scaffold rather than the final conceptual model.

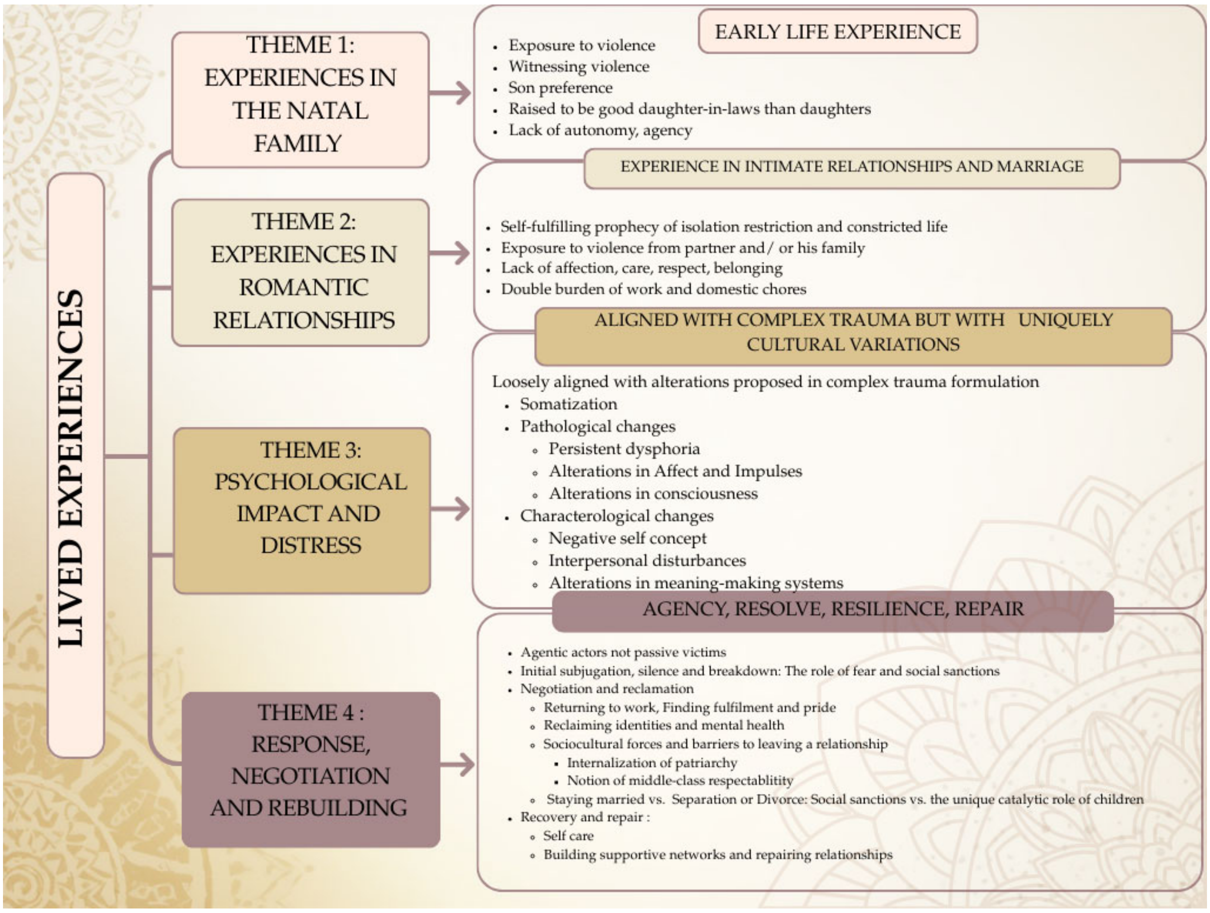

Figure S1. Early thematic Map

The final thematic structure presented in the Results section reflects subsequent refinement through iterative engagement with both data and theory.

Tables S3A–S3B: Coding, theme construction, reorganization and mapping to the SPECTRA domains. These tables present the stepwise analytic pathway from raw data to conceptual domains. The first column provides **illustrative analytic codes**, generated and synthesized from narrative data. The second column presents the **initial subthemes**, which were inductively organized from these codes. The third column shows the **analytic consolidation of initial subthemes**, achieved through iterative comparison and abductive engagement with relevant literature, resulting in **refined (final) subthemes and themes**. The final column maps these themes onto the corresponding **SPECTRA domains**. This structure shows the progression from descriptive coding to theoretically informed abstraction.

Notably, additional analytic reorganization was required for Themes 3.2 and 3.3, which were merged at the domain level into **Relational Captivity**, reflecting their shared underlying relational processes.

Table S3A. Theme 3.1 (Experiences in the natal family): Mapping to the SPECTRA Domain – Developmental Psychic Embedding.

**Illustrative Codes** (Annotated)	Initial **Subthemes**	Final Subthemes	Final **Themes**	**SPECTRA Domain**
"Beaten by father" (O); "Humiliated by grandfather" (O); "Saw father beat mother" (O); "Witnessed son preference" (S × O); "Strained relationship with parents" (Po) "Experienced early sexual exposure"(T x O) ; "Grandfather used to humiliate" (O)	Physical Violence experienced as a child<br>Sexual violence experienced as a child<br>Emotional abuse experienced as a child<br>- Son preference<br>- Neglect<br>- Humiliation<br>- Strained relationship with family	• 3.1.1 Adversities in childhood	**3.1 Experience in the natal family**	**Developmental Psychic Embedding**
"Mother monitored movements" (O × S); "Not allowed to go out" (P × S); "Career decided by family" (S × P); "Respectability prioritized over ambition" (S × T); "Girls have to adjust" (S)	Restriction of agency and autonomy	• 3.1.2 Learning to be a woman from girlhood: Normalization of gendered norms during early socialization<br>• 3.1.3 Raising the perfect future daughter-in-law, rather than a daughter—the tyranny of "adjustment"		

Table S3B. **Theme 3.2 (Experience within marriage and/or relationships: Relational control within extended family structures) and Theme 3.3 (Coercive and unpredictable relational contexts resembling prolonged captivity) Merged and mapped on to SPECTRA domain Relational Captivity.**

<table>
<tr><th>Illustrative Codes (Annotated)</th><th>Initial Subthemes</th><th>Final Subthemes</th><th>Final Themes</th><th>SPECTRA Domain</th></tr>
<tr><td>“Not allowed to work” (P × S); “Phone monitored” (P); “Had to ask permission” (P); “Humiliated by in-laws” (O × Po); “Cut off from natal family” (P × S)</td><td>Oppressive marital families</td><td>• 3.2.1 A self-fulfilling prophecy of a fundamentally different world in the marital home</td><td rowspan="3">3.2 Experience within marriage and/or relationships: Relational control within extended family structures</td><td rowspan="4">Relational Captivity (3.2 +3.3 merged)</td></tr>
<tr><td>“Never had help” (O x P); “Typical Indian Bahu” (Po X S)</td><td>Work-life balance and domestic chore load</td><td>• 3.2.2 Work and life: A double-edged sword</td></tr>
<tr><td>“hit by husband” (O)
“Sexual coercion” (O × P)
“Unwanted sex from husband” (O × Po)
“Sexual humiliation” (O × Po × S) “Got humiliated” (O × Po), “Humiliated in front of guests” (O × Po × S) “Not good enough” (Po × S) “Belittled publicly” (O × Po × S)
“Financial exclusion” (P × S)
“Decision exclusion” (P × S)</td><td>Violence from intimate partner
-Physical Violence
-Sexual Violence
-Emotional Abuse
- Financial abuse
Lack of affection
Exclusion from decision making</td><td>• 3.2.3 A world of hurt: Experience of typologies of violence</td></tr>
<tr><td>“Threats of harm” (O); “Rules change suddenly” (P) “Petty rules examples” (T x O); “Walking on eggshells” (Po × P); “Emotional manipulation” (Po × P); “Unpredictable outbursts” (O × P); “Broke things of emotional value” (T x O);</td><td>Convergence with Herman’s prolonged captivity like relationships
- Psychological domination
- Creation of a willing victim
- Isolation
- Identity erasure
- Capricious rules
- Threatening violence rather than enacting</td><td>• 3.3.1 Threats of harm
• 3.3.2 Inconsistent enforcement of trivial demands
• 3.3.3 Emotional manipulation and unpredictable violent outbursts</td><td>3.3. Coercive and unpredictable relational contexts resembling prolonged captivity</td></tr>
</table>

Tables S3C–S3D: Analytic mapping of coded data to subthemes and SPECTRA domains. These tables present the organization of analytic material for Themes 3.4 and 3.5 using a structure that emphasizes mapping rather than full stepwise reconstruction. The first column presents illustrative data fragments (quotes or close paraphrases). The second column provides the corresponding analytic codes (refined), including open, processual, positional, and socio-cultural codes and their interactions. Where relevant, brief markers (e.g., "clustered as") indicate analytic consolidation of codes into higher-order groupings. The third column presents the subthemes and microthemes, as defined in Section S2.4, and their organization within the thematic structure.

While these themes were grounded in inductive coding, their development involved distinct analytic emphases. In Theme 3.4, coding was more directly shaped by abductive engagement with complex trauma literature, particularly in delineating overlapping subthemes and microthemes that allowed for multiple coding of narrative segments. In contrast, Themes 3.1–3.3 and 3.5 involved more extensive interaction across coding types, resulting in layered subtheme construction. Accordingly, Tables S3C–S3D foreground the mapping of codes to subthemes and domains, while retaining indicators of the interpretive processes through which these structures were generated.

Table S3C. Theme 3.4 (Psychological and Physiological Alterations): Mapping to the SPECTRA Domain of Psychological and Somatic Adaptations

Illustrative Data Fragments (Excerpts/Close Paraphrase)	Analytic Codes (Typed)	Subthemes and Microthemes
"everything hurts"; "I feel pain everywhere"; "I am always tired"; "I cannot sleep"; "Doctors find nothing wrong"	Somatic distress without clear medical cause (O × S); Chronic fatigue (O × Po); Sleep disturbance (O); Bodily expression of distress (O × S)<br>- Clustered as somatic distress	3.4.1 Somatization (Physical manifestations of trauma)
"I feel sad all the time"; "Nothing makes me happy"; "I cried for hours"; "I feel empty"	Persistent dysphoria and emotional depletion (Po); Anhedonia (Po); Emotional overwhelm (Po)<br>- clustered as generalized affective distress	3.4.2 Alterations in Affect and Impulses<br>→ 3.4.2.1 Persistent Dysphoria
"I get angry suddenly"; "I shout and then regret it"; "My emotions go out of control"	Emotional lability and reactive anger (Po × P); Reduced affect regulation (Po × P)<br>— clustered as affective instability	→ 3.4.2.2 Anger and emotional lability
"I act without thinking"; "I cannot control myself sometimes"	Impulsivity and loss of emotional control (P × Po)<br>— clustered as dysregulated impulse control	→ 3.4.2.3 Affect dysregulation, impulsivity
"I feel nothing now"; "I stopped reacting"; "I don't feel anything anymore"	Emotional numbing and affective shutdown (Po)<br>— clustered as affective blunting	→ 3.4.2.4 Apathy and numbness
"I don't want to live"; "Sometimes I have felt like like ending everything"; "There is no point anymore"	Hopelessness and loss of future orientation (Po); Suicidal ideation (Po)<br>— clustered as collapse of future orientation	→ 3.4.2.5 Hopelessness and suicidal ideation
"I zone out"; "I forget things"; "Everything feels unreal"; "I feel disconnected from myself"	Dissociation and detachment (Po); Memory disruption (Po); Altered consciousness (Po)<br>— clustered as dissociative responses	3.4.3 Alterations in Consciousness

**Illustrative Data Fragments (Excerpts/Close Paraphrase)**	**Analytic Codes (Typed)**	**Subthemes and Microthemes**
"Maybe it was my fault"; "I should have done better"; "I feel ashamed"; "I deserved it";	Internalized self-blame (Po); Moral attribution of abuse (Po × S); Shame and guilt (Po × S)<br>— clustered as negative self-attribution	3.4.4 Alterations in Self-Perception and disorganization in Personality<br>→ 3.4.4.1 Shame and Guilt
"I am not the same person"; "I feel broken"; "I am damaged"; " I have so many body issues"	Identity disruption and altered self-concept (Po); Sense of being fundamentally changed (Po) ; Self-criticism (T); Low self-esteem (T) -clustered as identity fracture	→ 3.4.4.2 Seeing self as damaged and fundamentally altered
"He didn't mean it"; "He was a very decent guy otherwise"; "When he is nice, he is the sweetest person-my pain melts away"	Emotional attachment to perpetrator (Po × S); Rationalization of abuse (Po × S)—clustered as relational entanglement	3.4.5 Alterations in relationship with the perpetrator
"I don't trust anyone"; "I avoid people"; "I cannot form relationships"; "People always hurt you"	Social withdrawal (Po × P); Avoidance of relationships (Po × P); Anticipation of harm (Po) — clustered as relational withdrawal	3.4.6 Alterations in Relationships with Others<br>→ 3.4.6.1 Social withdrawal and isolating oneself
"I cannot depend on anyone"; "Relationships don't last"	Mistrust and relational difficulty (Po) — clustered as relational mistrust	→ 3.4.6.2 Mistrust and difficulty in forming new relationships
"I don't need anyone now"; "One always has to be alert in relationships and use tact"; "I don't need any emotional support"	Altered expectations from relationships (Po) — clustered as negative relational schemas	→ 3.4.6.3 Altered expectations from relationships
"I ended up in similar situations again"; "Kept on getting from bad relationships one after the other"; "From frying pan to the fire"	Revictimization patterns (P × Po) — clustered as repetition of relational harm	→ 3.4.6.4 Revictimization
"This is my fate"; "Women have to tolerate"; "Nothing will change"; "I don't know what I believe in now"; "Divorce- Oh my God why?"	Fatalistic belief systems (S × Po); Endorsement of endurance norms (S); Loss of belief in change (Po); Religious meaning-making (S × Po) — clustered as meaning system restructuring (abductively aligned with complex trauma frameworks)	3.4.7 Alterations in Beliefs and Meaning-making systems

Table S3D: Theme 3.5 (Response, Negotiation, Resilience and Rediscovery): Mapping to the SPECTRA Domain of Negotiation and Adaptive Repair

Illustrative Data Fragments (Excerpts/Close Paraphrase)	Analytic Codes (Refined)	Subthemes and Micro themes
"I stayed quiet"; "I didn't say anything"; "If I spoke, things would get worse"; "I just accepted it"; "If I objected it was not heard anyway'	Silence as a survival strategy (Po × P); Compliance to avoid escalation (Po × P); Acceptance of subjugation (Po × S); Emotional shutdown and withdrawal (Po) — clustered as protective compliance	3.5.1 Initial response: Subjugation and breakdown
"I have no man-power; No father or brother"; "Divorce- Oh My God, Why?"<br>"Leaving was not an option"; "Where would I go?"; "No support from family" "Divorce is not for middle class women"	Honor-based beliefs sustaining endurance (S × Po); Marriage as non-exitable institution (S × P); Structural constraints on exit (S × P) Perceived inescapability of marriage (S × Po); Dependence on patriarchal relational structures (S × P)) — clustered as structural entrapment	3.5.2 Negotiating difficulty, resistance and reclaiming lives<br>→ 3.5.2.1 The Inescapability of the Marriage
"Separation felt safer than divorce"; "I needed some distance"; "I found a middle ground"	Separation as negotiated compromise (P × Po); Partial exit strategies (P × Po) — clustered as negotiated exit	→ 3.5.2.2 Separation as a Compromise
"People will talk"; "It becomes a spectacle"; "Everyone judges the woman"; "neighbours were poisoned against me"	Social surveillance and reputational threat (S × Po); Moral regulation through public scrutiny (S) — clustered as community regulation	→ 3.5.2.3 The Communal Spectacle and the Construction of an Ideal Victim
"My children gave me strength"; "I did it for them"; "They helped me decide"	Children as catalysts for resistance and change (S × Po) — clustered as relational motivation	→ 3.5.2.4 Children as a Catalyst
"I started working again"; "I reconnected with friends"; "I didn't stop despite objections from him"; "I have a large group of work friends now"; "I don't need him now";	Reclaiming autonomy through work (P × Po); Rebuilding social networks (P); Boundary-setting and relational renegotiation (P × Po) — clustered as rebuilding agency	3.5.3 Rediscovery and repair
"Now my husband respects more, he understands me more"; "Now I make all my decisions"; "He won't talk like that with me now"; "After the pandemic he shares more domestic chores"	Partial reconnection with intimate partner (T x P) ; Interdependence enhancing dialogue (T x P); Partner's animosity mellowed with age (T x P x Po) —clustered as adaptive repair	
"I take annual vacations"; "I really enjoy when I now go out with friends"'; "I read and garden to calm myself" ; "I volunteer now"	Building new hobbies (Po x P); Rediscovering old hobbies (Po x P); Learning to take time for oneself (T x P x Po) – Clustered as rediscovering self	
"I understand things differently now"; "I find peace in Taoism"; "I value myself more"; "I am taking better care of myself now"	Reconstruction of self and identity (Po); Restoration of self-worth (Po); Meaning reconstruction (Po × S) — clustered as identity reconstruction	

Code Types:

- **(O) Open Code** — Direct, descriptive coding of participant statements or events
- **(P) Process Code** — Captures actions, patterns, or ongoing relational dynamics (e.g., restriction, negotiation)
- **(T) Thematic Code** — Higher-level conceptual grouping emerging from repeated patterns
- **(S) Socio-cultural Code** — Reflects cultural norms, expectations, or structural influences (e.g., "adjustment," respectability)
- **(Po) Positional Code** — Captures how participants position themselves, others, or their identities within narratives

Code Interactions:

- **× (interaction)** indicates that a code reflects multiple analytic dimensions simultaneously
  - Example: *(Po × S)* = interaction between positional meaning-making and socio-cultural norms
  - Example: *(P × S)* = relational process shaped by socio-cultural constraints

File S10: Results supplement

S10.1 Full Thematic Map

- **3.1 Experience in the natal family**
    - 3.1.1 Adversities in childhood
    - 3.1.2. Learning to be a woman from girlhood: Normalization of gendered norms during early socialization
    - 3.1.3 Raising the perfect future daughter-in-law, rather than a daughter—the tyranny of "adjustment."
- **3.2 Experience within marriage and/or relationships:Relational control within extended family structures**
    - 3.2.1 A self-fulfilling prophecy of a fundamentally different world in the marital home
    - 3.2.2. Work and life: A double edged sword
    - 3.2.3 A world of hurt: Experience of several typologies of violence from romantic partners and husbands and their families
        - *Physical Violence*
        - *Sexual violence, coercion, deprivation and humiliation*
        - *Psychological abuse and violence:*
- **3.3 Coercive and unpredictable relational contexts resembling prolonged captivity**
    - 3.3.1 Threats of harm
    - 3.3.2 Inconsistent enforcement of trivial demands and petty rules
    - 3.3.3 Emotional manipulation and unpredictable violent outbursts
- **3.4 Psychological and physiological alterations in survivors are loosely aligned with Herman's prediction of sequelae in survivors of prolonged victimization**
    - 3.4.1 Somatization (Physical Manifestations of Trauma):
    - 3.4.2 Alterations in Affect and Impulses
        - *Persistent Dysphoria*
        - *Anger and emotional lability*
        - *Affect dysregulation, impulsivity:*

        - *Apathy and Numbness*
        - *Hopelessness and Suicidal ideation:*
    - 3.4.3 Alterations in Consciousness
    - 3.4.4 Alterations in Self-Perception and disorganization in Personality:
        - *Shame and Guilt*
        - *Seeing self as damaged and fundamentally altered*
    - 3.4.5 Alterations in relationship with the perpetrator
    - 3.4.6 Alterations in Relationships with Others:
        - *Social Withdrawal and isolating oneself*
        - *Mistrust and difficulty in forming new relationships*
        - *Altered expectations from relationships*
        - *Revictimization*
    - 3.4.7 Alterations in Beliefs and Meaning-making systems
- 3.5 Response, Negotiation, Resilience and rediscovery
    - 3.5.1 Initial Response: Subjugation and breakdown
    - 3.5.2 Negotiating difficulty, resistance and reclaiming lives
        - *The Inescapability of the Marriage:*
        - *Separation as a Compromise*
        - *The Communal Spectacle and the construction of an ideal victim*
        - *Children as a Catalyst*
    - 3.5.3 Rediscovery and Repair

S10.2 Supplementary tables from results section nested under themes, level one and level two subthemes

- **3.1 Experience in the natal family**
  - 3.1.1 Adversities in childhood

Participant	Quote
**Ray (29)**	*"My dad was the first one to abuse me psychologically, physically, in many ways since I was a small child". "He used to hit me and my mother all the time in front of people, and demean us, always shout and criticise and never say anything nice".*
**Damini (30)**	*Damini's mother, following the trauma of her husband's death, directed emotional abuse toward Damini: "My father had his demise when I was 17. And then my mother went into serious depression. And my mother stopped cooking. She stopped speaking. She had a very tumultuous relationship with me. She started saying very nonsensical things. She started behaving very badly towards me. She started saying things like "Your father died. Why did you not die? I wish you had also died." I was just a college-going student at that time. And then we had major, major financial problems."* Table 2: *"My father died when I was 17, and my mother went into severe depression—she stopped speaking, stopped functioning, and turned hostile towards me, even saying I should have died instead. I was still in college, and we were suddenly facing major financial problems."*

Table S4. *Illustrative excerpts from participants' narratives capturing early experiences of childhood adversity within natal family environments.*

  - 3.1.2 Raising the perfect future daughter-in-law, rather than a daughter—the tyranny of "adjustment."

Participant	Verbatim Quote
**Rhea** (59)	*"I was very influenced by Florence Nightingale when I was a kid and I wanted to become a nurse and that was objected to by the family. Because in our times, you know, nurses were considered to be very lower middle class or family so whatever it is, it was not considered to be a very good profession for girls. I was denied and my next option obviously was a schoolteacher. I was before I got married and I am still a schoolteacher. [Father was] running a business and in our days, daughters coming into business was not, especially in a, in other families they do it but in a middle-class family to which I belong, they did not believe in daughters going into the business.*

	Abridged to:<br>*"I wanted to become a nurse, inspired by Florence Nightingale, but my family objected—it wasn't considered a respectable profession for girls in our family. I was steered into teaching instead, and daughters from middle class homes were not encouraged to enter the family business."*<br><br>*Table 3.*<br>*We were brought up that way only that when girls get married, you have to adjust yourself with your in laws and you have to set yourself in the ways of the family and accept whatever they say"*
**Priya** (50)	*"Ambition…not really. I wasn't much ambitious, I would say. You know normal typical Indian - if I'd get a job, fine. Otherwise, get married and get settled"*

Table S5. *Illustrative excerpts on gendered shaping of aspirations and adjustment expectations*

3.2 Experience within marriage and/or relationships: Relational control within extended family structures

3.2.1 A self-fulfilling prophecy of a fundamentally different world in the marital home

Participant	Verbatim Quote
**Rhea** (59)	*"I started my job just the day I had given my last exam in my graduation and then I started doing my master's and I started my job. And I was quite a known teacher in my locality and I had a huge batch of students who were really close to me because I was very young and very close to my students. And suddenly what happened is I got married, it was settled. And soon after my marriage, everything changed. Before I was married, I used to sing, I was a Hindustani (Indian) classical singer and I was very much into it and presently, my voice doesn't say that I was a singer but in that time, I was. I was a very happy go lucky girl at that time then I got married into a very conservative family where I was told that I should not move out and work. My career stopped there and I was not allowed to work. For fifteen years, I was a total housewife."*<br><br>*Table 4.*<br>*"I had just started my career—teaching, doing my master's, even singing Hindustani classical music—and I was very happy then. But after my arranged marriage into a conservative family, everything changed. I was not allowed to work, and my career stopped. For fifteen years, I remained a housewife."*

Table S6. *Illustrative excerpt on fundamentally different marital home and life*

3.2.3 A world of hurt: Experience of several typologies of violence from romantic partners and husbands and their families

- *Physical Violence*
- *Sexual violence, coercion, deprivation and humiliation*
- *Psychological abuse and violence*

Participant	Type of Violence / Relational Experience	Quote / Excerpt
**Ray**	Physical violence	Experienced repeated physical assaults across multiple relationships, including being pushed in front of a car and being pinned and choked by a partner.
**Renu**	Physical violence	Reported being beaten multiple times within the first two years of marriage, alongside ongoing emotional abuse.
**Maya**	Physical violence (linked to alcohol use)	Described being slapped, pushed, and beaten by her husband during episodes of drinking; violence also extended to her son.
**Renu**	Sexual coercion	“I don’t know how to put it… multiple times… I was not ready… I used to just lock myself in the room to save myself.”
**Damini**	Sexual coercion / violation	Reported being forced into unwanted physical contact and sexual situations, including being intoxicated without consent.
**Ray**	Sexual humiliation and coercion	“He would have sex and then say he has no feelings… he pities me… it made the whole thing horrible.”
**Priya**	Sexual coercion / lack of agency	Acknowledged non-consensual sexual acts within marriage and having no control over contraceptive decisions.
**Rhea**	Sexual coercion (guarded disclosure)	Confirmed experiences of coercion but responded tersely, reflecting resignation and discomfort in elaboration.
**Priya**	Emotional / psychological abuse	Experienced sustained verbal humiliation, exclusion, and neglect; abuse often occurred in front of relatives with no intervention from in-laws.

**Renu**	Emotional abuse and social exclusion	Reported being humiliated daily and discouraged from interacting with guests, expected to remain in the background within the household.
**Ray**	Emotional abuse across relationships	Described repeated patterns of manipulation, abandonment, threats, ridicule, and withholding of affection across multiple partners.
**Damini**	Emotional abuse in relationships	Reported repeated emotionally abusive dynamics across past relationships, including coercion and manipulation.
**Maya**	Emotional abuse and humiliation	Husband insulted her regularly, especially when intoxicated, and humiliated her and her relatives publicly.
**Meera**	Emotional abuse within extended family	Endured prolonged verbal abuse from husband and mother-in-law, alongside social ostracism and chronic distress.
**Rhea**	Emotional abuse and in-law hostility	Reported humiliation from husband and in-laws, including derogatory comments about her widowed mother.
**Priya**	Financial abuse	Restricted access to money; depended on secret financial support from her father for personal expenses.
**Maya**	Financial neglect / economic abuse	Husband spent earnings on alcohol and other women, refused financial responsibility for children, creating chronic insecurity.
**Renu**	Economic control	Forced to resign from job after marriage, losing financial independence and professional continuity.
**Rhea**	Work restriction / financial control	Prevented from working for years after marriage, resulting in complete economic dependence.
**Meera**	Conditional financial autonomy	Allowed to work because her income was needed, but remained burdened with full domestic and caregiving responsibilities.
**Priya**	Lack of emotional bond	Reported absence of companionship, shared activities, or emotional care within marriage.

**Rhea**	Lack of emotional intimacy	Described early marriage as emotionally distant, with no mutual understanding or communication.
**Maya**	Relational breakdown	Husband's neglect, infidelity, and abuse resulted in long-term separation and absence of emotional connection.
**Renu**	Absence of relational attachment	Expressed consistent anger and contempt toward husband, with no indication of emotional closeness.
**Rhea**	Exclusion from decision-making	Reported that major decisions in early marriage were made by husband and his family.
**Priya**	Social exclusion	Excluded from husband's social circle; not included in outings or shared activities.

Table S7: *Illustrative excerpts and paraphrased accounts across participants highlighting multiple, co-occurring forms of violence and relational control—including physical, sexual, emotional, and financial abuse, as well as exclusion from decision-making, social life, and emotional intimacy—within intimate relationships and marital family structures.*

Participant	Quote
**Rhea** (59)	*"I had offered her a cake, we had gone out with my in-laws and mother. And my mother had the cake just like that. Immediately, I got a comment from my in-laws that oh my god she's a widow and she's having a cake. She was hurt but she never answered back, she never cried in front of them. I cried but she told me to learn to ignore it. I remember the day my mother had worn a coloured blouse with white saree. She loved to dress up and had a beautiful blouse, not like that present designer blouse but she was very fashionable. She wore a coloured blouse with a white saree and they commented "oh, a widow wearing a coloured blouse" . After my father's death, it was a nightmare for me. It was so difficult for me to accept all this. She used to come to my house only for a day or for lunch, and the way they used to give her the food, in those days, widows were treated like that only."*<br><br>*Table 5:*<br><br>*"I had offered her a cake… and immediately I got a comment… she's a widow and she's having cake*… When she wore a coloured blouse, they said, 'a widow wearing a coloured blouse*'… The way they treated her… it was a nightmare for me."*<br><br>**Traditionally, among Indian Hindus, widows are prohibited from wearing any color other than white, as well as from eating any kind of non-vegetarian food, including eggs, which are used in baking cakes. They are given bland and vegetarian food only, prohibited and ostracized from social celebrations like marriages , or festive occasions*

Table S7A: *Cultural regulation of women's dignity and respectability*

Theme 3.3: Coercive and unpredictable relational contexts resembling prolonged captivity

Participant	Strategy of Control (Captivity Mechanism)	Illustrative Excerpt / Paraphrased Evidence
**Ray**	Threats and intimidation	Experienced repeated threats and escalating aggression across relationships, creating persistent fear and vigilance.
**Damini**	Threats extending to family	Partners threatened harm and legal consequences for her family, producing prolonged fear and compliance.
**Maya**	Fear through violence and unpredictability	Husband's alcohol-fueled aggression created a constant state of alertness for her and concern for her children's safety.
**Meera**	Threat through unpredictability of in-law	Lived with a volatile and at times dangerous mother-in-law, requiring constant management and caution.
**Ray**	Arbitrary and shifting rules	Partner imposed inconsistent and unclear rules governing communication, with unpredictable enforcement.
**Renu**	Punishment for minor deviations	Small domestic lapses escalated into severe confrontations, including threats of eviction and forced expulsion from the home.
**Ray**	Walking on eggshells (anticipatory compliance)	Adjusted behavior constantly to avoid triggering anger, reflecting internalized vigilance.
**Ray**	Emotional degradation targeting identity	Partner used personal vulnerabilities (e.g., health-related traits) to humiliate and destabilize her sense of self.
**Damini**	Systematic humiliation and manipulation	Partners ridiculed her appearance, language, and background while using deception and threats to maintain control.

**Renu**	Public humiliation and shaming	Subjected to public reprimand by father-in-law, reinforcing control through social exposure and shame.
**Priya**	Escalating verbal abuse and humiliation	Experienced increasing hostility and humiliation within marital home, often in front of relatives who did not intervene.
**Maya**	Intimidation through destruction and aggression	Husband's violent outbursts included breaking objects and creating a hostile domestic environment.
**Priya**	Intimidation and aggression	Partner engaged in shouting, breaking household items, and creating fear through aggressive displays.
**Renu**	Isolation through control of communication	Access to phones and external communication repeatedly disrupted, limiting contact with support systems.
**Maya**	Social isolation enforced by partner behavior	Could not maintain social relationships due to husband's abusive conduct toward guests and visitors.
**Multiple participants**	Emotional manipulation and dependency creation	Gaslighting, threats of self-harm, and emotional instability used to foster dependence and prevent exit from relationships.
**Multiple participants**	Convergence of control strategies	Combined use of fear, unpredictability, isolation, and humiliation created sustained environments of constraint resembling captivity.

Table S8. *Convergence of coercive control strategies across participant narratives, illustrating how threats, unpredictability, emotional manipulation, isolation, and public humiliation operate in combination to produce sustained conditions of relational control resembling prolonged captivity.*

Participant	Quote
**Ray**	*"Well, everything is a rule. He keeps changing the rules. So even if I follow all his rules, he'll make up new rules after it's already done. Then he'll say – 'Oh, you are not supposed to do this. Why did you do that?' It's impossible to say. I don't think even he knows what the rules are. Because it's a game that he keeps. He loves changing every second. One day he'll be like – 'I love you so much', this - that and the next day – actually not even the next day, like in the same breath he will be like 'I don't care about you and you're nobody'." ; ""He just makes up problems even when there is none". "He constantly shifts, he's constantly changing. I can't figure him out". He also engages in extreme gaslighting: "He's like always calculating... So he's always gaslighting me"."*<br><br>*Table 6:*<br><br>*"Everything is a rule—and he keeps changing them. Even if I follow all his rules, he creates new ones after the fact and blames me. It's impossible to keep up. One moment he says he loves me, and the next, in the same breath, says I'm nobody."*

Table S9. *Capricious rule enforcement and unpredictability as mechanisms of control*

Participant	*Quote*
**Ray (29)**	*"He will say very, very hurtful things, very abusive things. Very loathsome things, very disgusting things. Very violent things…. He is always insulting me. He loses his mind, like his temper. When he loses it, he is a monster, like a complete monster. I think even worse than my dad, which I never thought was possible. He makes fun of my problems. He makes fun of everything I believe in. He makes fun of my disease even. He has called me an autistic retarded bitch. 'I can't deal with you anymore. You and your autism can go to hell' ".*<br><br>*Table 7*<br>*"He will say very, very hurtful things, very abusive things. Very loathsome things, very disgusting things. Very violent things…. He is always insulting me. He makes fun of my problems. He makes fun of everything I believe in. He makes fun of my disease even. He has called me an autistic retarded bitch. 'I can't deal with you anymore. You and your autism can go to hell' ".*
**Damini (31)**	*"Yeah, I was told my English is not good. I was so you know for simple things like pronouncing the word sexuality right you know I was told…I am pronouncing it wrong then if I pronounce this word like this in front of a public gathering I would be laughed at and and a a lot of and and throughout of this time uh somebody had once told me I look like a "bhains" (buffalo) you know, ? That my hands are not good, my feet are not beautiful and all sorts of things. All sorts of things. So, yes, they've tried to make me feel less confident. "Who will marry you?" One partner said, "You know, you don't have money. Your mother is so unwell. I'm the only one who can be with you. There's not going to be any man in this world trying to get married to a girl like you. So the only person whom you can come for refuge is me." I was literally told this on my face many times. So essentially it's like saying that you are isolated and you need me . And I'm the only one for you. And I believed that for some time*<br><br>*Table 7*<br><br>*"I was told my English is not good... if I pronounce this word like this in front of a public gathering I would be laughed at. Somebody had once told me I look like a bhains (buffalo). That my hands are not good, my feet are not beautiful "*

Table S10. *Humiliation and identity-based degradation as strategies of emotional manipulation and control*

Participant	Quote
Renu	*The individual who I was married to, he,.. he used to create such a ruckus. I mean he was… he used to go crazy. He has, or I don't know what issues he had. He has a temper. He is so angry. If he is angry he would do anything. …..One of the situations where I felt threatened was…. by 8:00 lunch and breakfast has to be ready usually because working family members go out for work , so I was busy in the kitchen, ..suddenly this idiot (the husband) comes and he pulls me with his hand and drags me out of the house, I don't know why that was…at but that moment he says that ' if you behave like this again I'm going to throw you out from my house'. I didn't realize what I had done, then later I found out that I forgot to set an alarm for that idiot at 7:30 am because I got up at 4:00 am. He has to go out at 8 am and I have to set an alarm for him at 7:30 am. That night I really forgot. I forgot and… he... he got late and missed his bus for the company …. Later I felt bad for that. The threat that "if you again do it I throw you out of the house" happened a couple of times, and after that he just threw the food. On other occasions he just throws off the cooked food, and like he is going gaga and he says 'you are doing too much- I'm going to leave you' ".*<br>*Table 8:*<br>*"He would go into a rage over small things. Once, when I forgot to set his alarm after waking up at 4 am to cook, he dragged me out of the house and threatened to throw me out. This happened multiple times—over minor mistakes, he would threaten eviction or throw away the food I had cooked."*
Ray	*He loses his mind, like his temper. When he loses it, he is a monster, like a complete monster. I think even worse than my dad, which I never thought was possible... He refuses to talk once he enters into this rage, which is like at random all the time, like anything can put him into a rage. Even if I follow all his rules – when I'm not supposed to call, how many seconds extra have I called — blah, blah, blah, all of that I have to follow. And even if I follow them, he will still find something to get angry at, abuse me, threaten me, block me and all sorts of stuff"*<br><br>*Table 8*<br>*"When he loses his temper, he becomes like a monster… anything can trigger it. Even if I follow all his rules—when to call, how long to speak—he still finds something to get angry about, abuses me, threatens me, or cuts me off."*

Table S11. *Unpredictable and disproportionately violent outbursts of anger as strategies to foster uncertainty and constant fear*

Participant	Excerpt
Renu	*So one of the things…. one of the things….when my phone used to ring he used to get angry…And I used…I used to take a few calls… and then I used to just come out of the room and either be in the kitchen because there was a helper all the time in the kitchen that was a safer place because if outsider is in the home no one was to dare to come to me. So yeah a few escaping things took place but still. However one fine day he broke my phone out of anger when I was trying to talk to my parent…"I was taken off the communication… like, I have my own phone which- okay I purchased five to six mobiles in those two years just because my phone used to get broken off (by husband)….just because I'm online I'm talking or chatting or I was on phone talking to people and uh they made sure that I'm not in touch with the outside world.*<br>Table 9<br>*"Whenever my phone rang, he would get angry. I tried to take calls in the kitchen where others were present because it felt safer. But he often broke my phone when I spoke to my parents. Over two years, I went through several phones because they kept getting destroyed—just to make sure I stayed cut off from the outside world."*

Table S12. *Isolation through control of communication and enforced disconnection from support systems*

Participant	Quote
**Renu**	*Um out of all this I just tried and somehow managed to come out and again this this try was to come out and make a call to my family saying that look this happening but that was unsuccessful attempt to me coming out but **uh what I did was Uh I just I… I ran down the staircase and I was just standing there. I didn't realize uh I was in my you know in my night wear like you know the long night suit. I didn't realize that and I was not wearing chappals (flipflops). I just ran down with a couple of coins of money like few coins so that I can [make a phone call from a booth]. My only intention was to just come out and talk. My bad time was when my father-in-law was just coming back home and that was the moment he just shouted in public saying that uh you look ... .how do I put that word in English? Okay. You look like an um a lady uh who just got out of bed, or something like an..illicit* lady ."*<br><br>**Morally loose woman who lets the world see her in her nightwear, out in public inappropriately or immodestly dressed*<br><br>*Table 10:*<br>*"He dragged me outside the house… and threatened to throw me out… I ran out just to make a phone call… and my father-in-law shouted at me in front of neighbours… saying I was dressed inappropriately… like a loose woman."*
**Ray**	*"He sees it right through me and maybe that's why he hurts me so efficiently. I used to think he doesn't have control over himself... but recently he admitted that he knows exactly what he's doing. He said I know how efficiently I can hurt you and I will keep doing it. He also says he will step up his efforts now to utterly destroy me emotionally"*<br><br>*Table 10:*<br>*"He told me he knows exactly how to hurt me—and that he will keep doing it, even step it up, to destroy me emotionally."*

Table S13. *Intentional gaslighting and public humiliation and control as mechanisms of compliance*

Theme 3.4 Psychological and physiological alterations in survivors are loosely aligned with Herman's prediction of sequelae in survivors of prolonged victimization

Subtheme	Microtheme	Participant	Excerpt
**3.4.1 Somatization**	Physical manifestations of trauma	Priya	"I started losing weight… my appetite was affected after continuous fights… I remember taking sleeping pills at that time."
		Ray	"I have endometriosis… adenomyosis… jaw pain, back pain, fatigue… difficulty sleeping."
**3.4.2 Alterations in Affect and Impulses**	Persistent dysphoria	Ray	"I cry a lot… very anxious… racing thoughts… flashbacks… I think I'm on high alert… sensitive to everything."
	Anger and emotional lability	Ray	"I have always had that feeling… that I might lose control… and if someone tells me to calm down it gets worse."
	Affect dysregulation, impulsivity	Renu	"I don't want to cry but unknowingly tears just come out… things get out of control."
	Apathy and numbness	Ray	"It reaches a point where I just have to be numb… otherwise it's impossible to feel that much."
	Hopelessness and suicidal ideation	Damini	"I was planning my death."
	Inhibited affect	Maya	"I have nowhere to go, no one to tell."
**3.4.3 Alterations in Consciousness**	Dissociation	Ray	"I used to feel like I'm watching myself… like an out-of-body experience."

	Memory disturbance	Maya	“I don’t remember.”
**3.4.4 Alterations in Self-Perception and Personality**	Shame and guilt	Priya	“He did make me feel guilty… I blamed myself… maybe I should not have said this.”
	Seeing self as damaged	Renu	“They realized I was not myself.”
**3.4.5 Alterations in relationship with perpetrator**	Trauma bonding / minimization	Ray	“When he is kind… it feels worth everything… all the pain melts away.”
**3.4.6 Alterations in Relationships with Others**	Social withdrawal	Ray	“I don’t have anyone… I stay by myself.”
	Mistrust	Renu	“Very, very difficult to trust people.”
	Altered expectations	Meera	“I don’t expect anything from my relationship anymore.”
	Revictimization	Damini	“All of them were abusive… I saw the red flags but ignored them.”
**3.4.7 Alterations in Beliefs and Meaning-making**	Hopelessness / loss of meaning	Ray	“It just seems endlessly bad… I don’t see light at the end of the tunnel.”

Table S14. *Selected excerpts illustrating psychological and somatic alterations across Theme 3.4, mapped to subthemes and microthemes; entries are illustrative rather than exhaustive and reflect key patterns identified in the analytic framework.*

Theme 3.5 Response, Negotiation, Resilience and rediscovery

Subtheme	Microtheme	Participant	Excerpt
**3.5.1 Initial Response: Subjugation and breakdown**	**Early normalization and compliance**	Priya	"I was very young… I thought maybe I shouldn't have said this… I blamed myself."
	**Psychological breakdown**	Renu	"I didn't realize what I had done… I was confused… everything kept escalating."
**3.5.2 Negotiating difficulty, resistance and reclaiming lives**	**The Inescapability of the Marriage**	Maya	"In those days… you have to adjust… there was no question of leaving."
		Rhea	"We were brought up that way… once married, you accept whatever the family says."
	**Separation as a Compromise**	Renu	"I had to come back… there was no other option at that point."
		Priya	"I did leave… but there was guilt… maybe I could have stayed."
	**The Communal Spectacle and the construction of an ideal victim**	Renu	"They shouted in front of everyone… like I had done something wrong."
	**Children as a Catalyst**	Meera	"For my daughter… I had to think differently… I couldn't continue the same way."
**3.5.3 Rediscovery and Adaptive Repair**	Re-engagement with self and work	Renu	"I tried to get back to studies… it was difficult but I continued."
	Seeking support / therapy	Damini	"I finally went to therapy… I couldn't handle it anymore."

	Partial recovery / recalibration	Meera	"I don't expect anything emotionally now… I manage on my own."
	Ongoing struggle with identity	Ray	"I don't know who I am anymore… I'm still trying to figure it out."

Table S15. *Selected excerpts illustrating participants' responses, negotiation processes, and attempts at adaptive repair across Theme 3.5; entries are illustrative and reflect key trajectories rather than exhaustive coverage.*

Participant	Excerpt
**Rhea**	*"Keep quiet and never protest. If you protest, there's no way out to resolve or calm it (conflict) down. The only way was keep your mouth shut, listen to it. I couldn't fight back, I couldn't say anything. I'm not, like I, I still […] I keep quiet, I can't say anything."*<br><br>*Table 13*<br>*"Keep quiet and never protest… If you protest, there's no way to calm it down. The only way was to stay silent. I couldn't fight back… I still keep quiet."*
**Maya**	*I never used to shout back a lot. Like I will say once or twice, I'm telling you about everyday. Like why are you doing these things. It's better if you go away from the house, that's all. And after that I didn't say much"*<br>*"Even if I'm angry I used to keep quiet so that I don't utter any harsh words." Maya when asked about how she dealt with the emotional and verbal abuse perpetrated by her mother-in-law"*<br><br>*"See, actually I told you I couldn't fight back, I couldn't say anything. I'm not, like I, I still couldn't, I keep quiet. Like even if, my sons also, if they say (speaks Bengali), I keep quiet, I can't say anything."*<br>*Table 13*<br>*"Even if I'm angry, I used to keep quiet so that I don't say anything harsh…never reply back"..*
**Priya**	*"I used to stay quiet to not trigger any more of these problems. I would say, "I used to suffer a lot within myself."*<br>*Priya when asked about conflict resolution with her partner "So how would you respond in the moment when it happened - when he was shouting at you?*<br>*Well, I used to keep quiet and just went back to my room or something like that. (inaudible). And did you feel scared wen he used to shout at you?*<br>*Not really scared. But yes, I used to feel very bad. Not scared, I wouldn't say scared.*

	Did you take any steps to control this kind of behaviour of your partner like talk to him or?<br>*No no*<br>*How do you make amends after the fight or would you just ignore the fact that the fight had happened?*<br>*Yes, I would ignore it. Yes, I ignored it always.*<br>*Table 13*<br>*"I used to stay quiet to not trigger any more of these problems. Kept quiet, went to my room, Tried to ignore all of it- but I used to suffer a lot within myself."*
**Meera**	*When her husband shouted at her Meera states: "I kept quiet. I didn't know what to reply". To resolve conflicts with her in-laws, Meera said: "I tried to avoid everything". She often "used to stop talking I never used to talk to them".*<br>*Table 13*<br>*"I kept quiet. I didn't know what to reply. Just avoid"*

Table S16: *Silence and non-confrontation as enforced responses within conditions of subjugation*

Participant	Excerpt
**Rhea**	Despite their initial subjugation and suffering, none of the participants were passive victims, but active agentic actors in their own lives. This is best exemplified in Rhea's words who spent 15 years of her marital life as a subservient daughter-in-law.<br>*"After 16 years, I realized that I can come out of it. I took that decision that every woman should be independent, and should have an identity. That I got from my mother."*<br>*"Then one fine day I took the decision that enough is enough and I should move out. Children are now old enough, they can take care of themselves, they can feed themselves and go to school. And it was my luck that the day I thought of it, the very next day I got an interview. I applied and I got an interview and that very week, I got the job."*<br>She remembers having to fight everyone at her marital home for her freedom to earn again, but she pushed past it.<br>*"That was a start, though it was not accepted by all in the family at that time, things were not in my favour but that was the push I got from my inner feelings and heart and this way, I did not listen to anyone. I forgot all the words they had told me like "girls do not work". Of course, after that, everyone accepted. Initially, it was not accepted by anyone, not even my husband."*<br>However with time, and her sustained effort, her husband and her family's stance changed and currently her daughters-in-law are also employed according to their wishes.<br>*"But later on, now everyone believes that yes, even women should have an identity. It's not for the family finances but for their own fate, for their own identity. For their own independence, every girl should work. So that was my achievement or that is my achievement."*<br>*Table 14*<br>*"After 16 years, I realized I could come out of it… every woman should have an identity."*<br>*"One day I decided enough is enough… the next day I got an interview, and that week I got the job."*<br>*"No one accepted it at first—not even my husband—but I did not listen. Later, everyone accepted… now even the daughters-in-law work."*

Table S17: *Reclaiming work and identity as an act of resistance within constrained marital environments*

Participant	Excerpt
**Rhea**	Marriage is often characterized as a sacred and indissoluble institution, particularly for the middle class, whose identity reinforces conformity and tradition. Rhea's words reflect this, even as she was being subjected to considerable psychological abuse<br>*"Thinking of divorce, thinking of separation was also, we couldn't dream of it, it was not easy."* (Rhea, 59)<br>Rhea had mentioned that in the lowest phases of her marriage she had wondered what would it be like to separate and have a life of her own, but when we probed deeper, trying to know if she ever discussed this with anyone, or ever try to take any action towards separation, she indicated that she had never actually done any of that. When we asked her what prevented her, she said,<br>*"It was a social taboo which we thought "my god, why" "* (Rhea, 59)<br><br>Her reluctance to separate can be traced back to her husband's family being involved in providing for her mother (who was widowed right after Rhea's marriage) and her younger sisters for many years. Ironically, Rhea stated that eventually her son got financially settled and had a decent income as a chartered accountant, whose income was the mainstay for her family's provision yet she failed indebted and "dependent" on her marital family's largesse , and their reminders about it<br><br>*"But I heard from my in-laws also. Though my son is a very established man, a chartered accountant, he was working in a very good company at that time. It was nothing like that [financial burden] but just you know, that feeling comes into the mind of my in-laws that "oh my god, now what will happen? my daughter in law is not working and her mother, her sister. Who will take care of them now?" Because women at that time had to be dependent. My mother is also dependent, my sister is also dependent, who will take care of them . Many other issues also - going out, meeting someone, talking to someone, those were also issues. These are the things I had to face, I told you, everything I used to accept. I used to never protest the things told to me. After 16 years, I realized that I can come out of it."*<br><br>*"Only during our times, girls who used to think that had a very strong financial background or a political background or a very strong manpower also, that is also required - they have brothers and all. So you don't have a brother means you have no one on your parents side. I felt that I don't have a brother, and they (in-laws) got their son married to me and now my father passed away, what will happen to my mother. Now the daughters only have to take care of her. I'm not working, I'm not earning so the whole responsibility comes on the front so that mentality was also there."*

	After the ritual of kanyadaan (where a father donates the ownership of the bride to her husband) during a traditional Hindu marriage, a woman can only think about challenging that ownership through a divorce if she has a strong masculine backing from her natal family, either through her father or brother, to ensure financial stability, residence, and societal safety net. For Rhea, not having brothers and losing her father early, along with her family's financial background, made the concept of divorcing her husband an impossible pursuit, unconsciously forcing her to stay in a psychologically abusive marriage.<br><br>*Table 15*<br><br>*"In our time, only girls with strong financial or family backing could think of leaving. I had no brother, my father had passed away, and I wasn't earning—so all responsibility was on me. That also shaped how I stayed."*
Priya	*Priya explicitly stated her "fear of the society" was the main reason she stayed silent and delayed separating despite a loveless marriage in which she was subjected to psychological abuse repeatedly.*<br>*"Afraid, yeah kind of. Because I had such an idea that this isn't gonna work. But I was afraid of society. That was the main thing which was in my mind." (Priya,50)*<br><br>*Table 15*<br>*"I knew this [her marriage] wouldn't work… but I was afraid of society."*
**Meera**	*"It is not a solution to leave marriage- you have to use your brain- I used tact."*<br>*Table 15*<br>*"That is not a solution to walk out of the marriage."*

Table S18: *Social stigma and moral expectations as constraints on exiting marriage.*

Participant	Excerpt
**Meera**	*While talking about how her mother in law turned their neighbors and community against Meera by malingering continuously " I never used to understand. And till her last breath also, she did many.. of..uh.the things that... Socially I was very much affected - but I tried to avoid everything. That was my mental..uh..that brought my mental trauma. Even, I'm a heart patient [because of the] mental trauma I had to suffer"*<br><br>*On her husband'ss dissatisfaction with her because she moved her mother-in law to an eldercare for proper management of psychiatric symptoms*<br>*"Even till now he is very dissatisfied because I kept her at, no, that I had to keep her in an old age home."*<br><br>*Table 16*<br><br>*"Socially I was very much affected… that brought my mental trauma."*<br><br>*"Even now, he is still dissatisfied that I kept her in an old age home."*

Table S19: *Community-level stigma and reputational harm as extensions of relational conflict , Moral scrutiny and social judgment in enforcing normative expectations of caregiving and sacrifice*

Participant	Excerpt
**Maya**	*"(About abuse) my elder son said (to her husband), 'Let us stay separate and you stay separate, and let us stay in peace."*
**Meera**	*Meera talks about her daughter as a source of encouragement and support to move ahead in life: "I was very much traumatized And it was u those uh those days that my uh daughter came in my uh and uh and she was beside me and she said move on. Don't think about the family. Yeah. Move on with your friends. Where are your childhood friends? I said I don't know. That time I did not know where where are they. I did not have any clue because I had to shift my home. account. So after my marriage, I did not have anybody any of my uh childhood friends except my sisters."*<br><br>*Following this encouragement from her daughter, Meera explains how she was able to reconnect with her social network:*<br><br>*"And then uh at that time because of Facebook I could gather my uh childhood friends, my colleges and everything. Yes. And then after that again my uh social life broadened."*<br><br>*The MIL's condition and abusive beliefs posed a threat to Meera's daughter's health and well-being:*<br>*• Effect on child: The MIL's behavior "had a effect on my child also".*<br>*• Daughter's suffering: Meera recounted that because of the MIL's "abusive belief my daughter had to suffer and because she used to suffer she she used to uh she used to become ill and she had to be hospitalized many a time during her childhood". When Meera's husband was actively resisting her attempt to place her mother-in-law under psychiatric eldercare, Meera was "on the verge of leaving him [her husband] also," suggesting that her daughter's flourishing factored into her decision-making process about her marriage and moving forward. In her mind"*<br><br>*Table 17*<br>*"I was very traumatized… my daughter told me, 'Move on. Don't think about the family. Find your friends.'"*<br><br>*"Through Facebook I found my old friends again… my social life opened up."*<br><br>*"Only time I considered leaving marriage was if I could not place my mother-in-law in an eldercare, for my daughters' sake."*

Table S20. *Children as emotional anchors and catalysts for change, enabling action and social reconnection*

Participant	Excerpt
**Renu**	*"I needed the strength to face the system… to raise my voice and put it out there."*
**Damini**	*"I think now that I'm you know I'm with somebody I'm married that has also got a lot of change the way my spouse is has also changed my outlook towards life<br><br>right now I'm less I'm more confident of uh you know the other person. I don't think I'm I don't feel that vulnerable. "In her current marriage, she took action against her regressive in-laws by "putting her foot down" and shouting, which "changed the game completely"- "They could never think I would shout back"<br>Table 18<br>"Now I speak back… they don't talk to me the same way anymore."*

Table S21. *Reclaiming voice and agency as part of adaptive repair*

Table No.	Section / Theme Location	SPECTRA Domain	Central Mechanism Illustrated	Analytic Importance / Why the Excerpt Matters
**2**	3.1.1 Childhood adversity	Developmental Psychic Embedding	Early exposure to violence, fear, parentification, and loss within natal family	Establishes developmental substrate for later normalization of harm and uneven power
**3**	3.1.2 Girlhood socialization	Developmental Psychic Embedding	Restriction of mobility, peer contact, and community surveillance during early gender socialization	Demonstrates how control becomes internalized as normative protection and anticipatory compliance
**4**	3.2.1 Marital rupture	Relational Captivity	Abrupt transition into highly restrictive marital environment	Shows marriage as developmental rupture and first sustained encounter with coercive adult relational control
**5**	3.2.3 Intergenerational control	Relational Captivity	Cultural regulation of widowhood, dignity, and respectability	Demonstrates extension of patriarchal control across generations and beyond the immediate survivor
**6**	3.3.2 Arbitrary rules	Relational Captivity	Capricious rule enforcement and shifting demands	Illustrates how unpredictability fosters chronic vigilance and self-monitoring
**7**	3.3.3 Emotional manipulation	Relational Captivity	Humiliation, appearance-based	Shows erosion of self-worth and increased

			ridicule, identity degradation	psychological dependence
**8**	3.3.3 Violent outbursts	Relational Captivity	Disproportionate anger and unpredictable rage	Demonstrates fear conditioning and constant anticipation of triggers
**9**	3.3.3 Isolation	Relational Captivity	Communication disruption and enforced disconnection from support	Illustrates systematic social isolation as a captivity mechanism
**10**	3.3.3 Public humiliation	Relational Captivity	Gaslighting, reputational shame, public degradation	Shows how coercive control extends into community surveillance and shame regulation
**13**	3.5.1 (if present in current file, verify numbering)	Negotiation and Adaptive Repair	Early subjugation / endurance response	Demonstrates constrained survival strategies under continued relational threat
**14**	3.5.2	Negotiation and Adaptive Repair	Delayed resistance and recalibrated negotiation	Shows shifts in agency thresholds under prolonged trauma
**15**	3.5.2 Social legitimacy	Negotiation and Adaptive Repair	Fear of stigma and legitimacy as victim	Illustrates sociocultural mediation of response decisions
**16**	3.5.2.3 Community stigma	Negotiation and Adaptive Repair	Moral scrutiny and reputational judgment	Shows how caregiving norms and social legitimacy regulate women's action
**17**	3.5.2.4 Children as catalyst	Negotiation and Adaptive Repair	Children as emotional anchors and catalysts for change	Demonstrates relationally mediated

				thresholds for action and exit
**18**	3.5.3 Adaptive repair	Negotiation and Adaptive Repair	Reclaiming voice, speech, and self-assertion	Illustrates emergent agency and post-trauma relational reorganization

Table S22. *Analytic mediation matrix for boxed narrative excerpts in the main Results section. For each excerpt, the corresponding SPECTRA domain, central relational or psychological mechanism, and its analytic contribution to theory-building are explicitly identified to enhance interpretive transparency and respond to reviewer concerns regarding direct post-excerpt mediation.*
